# Ubiquitination of DDX21 by HERC2 induces a dormancy-like phenotype via the NUCKS1-p21/p27 axis to promote radio-resistance in colorectal cancer cells

**DOI:** 10.1038/s41419-026-08811-0

**Published:** 2026-05-03

**Authors:** Yuqi Xiao, Mingyuan He, Han Yao, Yimeng Song, Yu Hu, Xinglong Liu, Yang Bai, Jianghong Zhang, Chunlin Shao, Yan Pan

**Affiliations:** 1 Institute of Radiation Medicine, Shanghai Medical College, Fudan University, Shanghai, China; 2 Department of Radiation Oncology, China-Japan Union Hospital of Jilin University, Changchun, China; 3 Ministry of Education and Training of Children’s Hospital Affiliated to Fudan University, Shanghai, China; 4 Department of Pathology, China-Japan Union Hospital of Jilin University, Changchun, China; 5 Department of Radiation Oncology, Shanghai Proton and Heavy Ion Center, Fudan University Cancer Hospital, Shanghai, China

**Keywords:** Colorectal cancer, Radiotherapy, Mechanisms of disease

## Abstract

Cellular dormancy in colorectal cancer (CRC) significantly contributes to therapeutic resistance, tumor recurrence, and metastasis, resulting in poor prognosis. However, the underlying molecular mechanisms remain poorly understood. Here, we used spheroid culture combined with serum deprivation to enrich and identify dormancy-like CRC cells in vitro and characterized their dormancy-like phenotype by G0/G1 phase arrest, suppressed proliferation and radio-resistance. Compared to proliferative cells, dormancy-like CRC cells maintained similar tumor formation capacity but displayed higher PD-L1 expression level and enhanced migratory ability, indicating greater aggressiveness. Proteomics analysis revealed DDX21 was significantly downregulated in dormancy-like CRC cells, and analysis of clinical data showed an inverse correlation between DDX21 and PD-L1 in CRC patients. This is consistent with the findings that knocking down DDX21 markedly increased PD-L1 levels, suggesting a role for DDX21 in immune evasion. Importantly, overexpressing DDX21 reversed the radio-resistance of dormancy-like CRC cells. Mechanistically, DDX21 downregulation induced a dormancy-like phenotype via p38MAPK activation and AKT suppression to inhibit cellular growth. Furthermore, DDX21 bound to the *NUCKS1* promoter, and its downregulation reduced NUCKS1 transcription, leading to elevated p27/p21 levels, which reinforced G0/G1 arrest. We also identified HERC2 as the E3 ligase mediating DDX21 degradation via K48/K63-linked polyubiquitination, dependent on the DDX21 helicase core domain. In conclusion, our findings establish DDX21 as a crucial regulator of dormancy-like phenotype in colorectal cancer cells and highlight its potential as a biomarker for dormancy-like tumor populations, radioresistance and poor clinical outcomes in CRC.

## Introduction

Colorectal cancer (CRC) is one of the most prevalent cancers worldwide, ranking third in incidence and second in mortality among all cancers [1]. Although recent advancements in oncological treatment have generated significant improvement in CRC patients, many patients undergoing adjuvant therapy ultimately succumb to a relapse of systemic disease [2]. Current treatment regimens, such as chemotherapy and radiotherapy, target the proliferating CRC cells, based on the theory that the proliferating cancer cells are highly sensitive to therapy. However, tumor heterogeneity means not all cells in the tumor are equally sensitive to these treatments. A direct cause of therapy resistance and relapse is the presence of dormant tumor cells. Tumor cell dormancy refers to a dynamic and reversible state in which disseminated cancer cells persist in a quiescent, non-proliferative state for extended periods, evading conventional therapies and driving late relapse [3]. This phenomenon is distinct from generic growth arrest (such as transient cell cycle arrest induced by acute stress or DNA damage) and senescent in several key aspects [4, 5]: (a) true dormant cells undergo reversible G0/G1 phase arrest with sustained p27 upregulation. (b) They possess long-term survival capacity and stress tolerance. (c) They possess high epigenetic plasticity, enabling adaptation to microenvironmental cues. (d) They can undergo immune evasion through downregulation of antigen presentation machinery. Therefore, a better understanding of the molecular mechanisms governing tumor dormancy may provide promising strategies to target dormant cancer cells, enabling more effective monitoring and control of these cells to prevent disease recurrence.

Dormancy characteristics have been recognized in diverse cancer types, including breast cancer [6]. head and neck squamous cell carcinoma, lung cancer, prostate cancer [7], and colorectal cancer [8]. Tumor cell dormancy involves a sophisticated regulatory network that integrates cell cycle control [9]. signal transduction pathways [10]. tumor microenvironment interactions [11]. and epigenetic regulation. Current research has identified several signaling pathways involved in dormancy regulation, particularly the PI3K/Akt/mTOR, MAPK/ERK, Wnt/β-catenin, and Notch pathways [12]. Among these, the MAPK/ERK signaling pathway is well characterized, with a p-ERK^low^: p-p38^high^ signaling ratio being widely used to characterize dormant cells [13]. Meanwhile, dormant tumor cells exhibit chemoresistance and maintain a reversible drug-tolerant state, allowing for their proliferative recovery and clonal expansion when the drug is withdrawn [14]. In this regard, recent clinical trials have targeted dormant tumor cells to prevent disease recurrence. For instance, an ongoing clinical trial (NCT03572387) is investigating the effectiveness of 5-azacytidine combined with all-trans retinoic acid in maintaining disseminated prostate cancer cells in a permanent dormant state - an approach that has also been confirmed to be effective in head and neck squamous cell carcinoma (HNSCC) and breast cancer cells at the fundamental research level [15]. Meanwhile, the ongoing NCT04841148 trial is evaluating the combination of Avelumab or Hydroxychloroquine with Palbociclib to eradicate dormant tumor cells and mitigate bone marrow metastases in breast cancer patients. Despite these advances, clinical trials and studies on dormant tumor cells are still hampered by suboptimal detection methods and inadequate, unreliable biomarkers. Moreover, the relationship between radiation and CRC dormancy, as well as the cellular mechanisms governing dormancy regulation, is poorly understood. Further investigation is warranted to elucidate these crucial aspects of CRC biology.

DDX21, a DEAD-box RNA helicase, is recognized as a multi-functional oncoprotein that drives tumor progression in various cancers, including colorectal cancer. Previous studies have established that DDX21 promotes cancer cell proliferation and transcriptional activity by regulating rRNA processing and facilitating ribosome biogenesis [16, 17]. Its interaction with chromatin modifiers such as WDR5 further underscores its role in amplifying transcriptional programs that sustain tumor growth [18]. Additionally, Chip-seq analysis reveals that the regions bound by DDX21 are enriched with transcription factor motifs recognized in cell growth and proliferation, such as E2F, STAT1, NRF1 and ETS [16]. These findings suggest that DDX21 plays significant roles in cancer cell biology. Parallel to this, HERC2, a large E3 ubiquitin ligase, is a well-established guardian of genome stability. It plays a critical role in the DNA damage response, primarily through its regulation of p53 stability and its involvement in the repair of DNA double-strand breaks [19, 20]. However, despite the clear roles of DDX21 in proliferation and of HERC2 in DNA damage response, their functions in the context of tumor dormancy, a state of therapeutic resistance and long-term recurrence risk, remain completely unknown. It is also unknown whether a functional relationship exists between HERC2 and DDX21.

Given that true tumor dormancy cannot be fully recapitulated in vitro, we employed spheroid culture combined with serum deprivation to establish a “dormancy-like state” in colorectal cancer cells. Using this model, we demonstrate that dormancy-like CRC cells exhibit significantly enhanced radio-resistance and PD-L1 expression compared to their proliferative counterparts. We also identified a previously uncharacterized axis, mediated by DDX21 degradation and subsequent downregulation of NUCKS1, that governs the dormancy-like phenotype of CRC cells. In addition to its established role in promoting proliferation, we find DDX21 downregulation also serves as an adaptive mechanism that drives CRC cells into a dormancy-like state, thereby contributing to therapy resistance. Furthermore, we identify HERC2 as the upstream regulator that targets DDX21 for proteasomal degradation via K48- and K63- linked polyubiquitination. This reduction in DDX21 leads to diminished CyclinD1 and NUCKS1 expression, leading to the accumulation of p21 and p27, G0/G1 cell cycle arrest, and entry into dormancy. Our findings thus reveal a novel, context-specific function of DDX21 and establish the DDX21-NUCKS1-p21/p27 axis as a crucial mechanism underlying CRC cell dormancy, presenting a new conceptual framework for understanding and monitoring minimal residual disease.

## Materials and methods

### Cell culture and irradiation

Human CRC cell lines DLD-1 and HCT116 were obtained from the Shanghai Cell Bank (Shanghai, China). All cells were cultured in RMPI-1640 medium (Gibco, Grand Island, USA) supplemented with 10% fetal bovine serum (FBS) (Gibco), 100 U/ml penicillin and 100 μg/ml streptomycin (P/S) (Gibco) in a humidified atmosphere of 5% O_2_ at 37 °C. All cell lines authenticated by Short Tandem Repeat (STR) assay were mycoplasma-free. To generate dormancy-like cells from the cancer cell lines, the dissociated cells were cultured in ultra-low attachment dishes (Corning, New York, USA) using serum-free DMEM/F12 medium (Gibco) supplemented with bFGF (20 ng/ml) (PeproTech, New Jersey, USA), EGF (40 ng/ml) (Gibco), N2 (1:100) (Gibco), and P/S for 3–14 days. The dormancy phenotype was demonstrated to be reversible upon re-stimulation with serum.

For irradiation, adherent or spheroid cells were exposed to γ-ray or X-ray at room temperature, followed by other indicated experiments. γ-rays was performed with a dose rate of 0.73 Gy/min using a 137Cs γ-irradiator Gammacell-40 (Nordion Inc., Toronto, Canada). X-ray was performed at a dose rate of 2 Gy/min (X-RAD 320, PXI Inc., North Branford, USA).

### Patient samples

Ten CRC specimens were collected from patients who underwent radical resection of colorectal cancer or endoscopic biopsy before radiotherapy at the China-Japan Union Hospital of Jilin University. The detailed clinical information of patients was shown in Table S1. Tumor progression and clinical outcomes in these CRC patients were evaluated through computed tomography (CT) imaging both before and after radiotherapy. All clinical tissue-related investigations were conducted in line with international ethical standards as stipulated in the Declaration of Helsinki of the World Medical Association. Informed consents were obtained from all subjects. For the experiments using human participants or data, prior approval was obtained from the China-Japan Union Hospital of Jilin University (Jilin, China) (2022-KYYS-048).

### Primary CD8 + T cells isolation and activation

Peripheral blood mononuclear cells (PBMCs) were isolated from healthy donors by density gradient centrifugation using Human Lymphocyte Separation Medium (Solarbio, Beijing, China) according to the manufacturer’s instructions. The study protocol was approved by the Ethical Committee of Fudan University, and informed consent was obtained from all subjects. CD8 + T cells were then purified by negative selection using CD8-negative microbeads (BioLegend, San Diego, CA, USA) and magnetic separation. Purified CD8 + T cells were cultured in RPMI 1640 medium supplemented with 10% fetal bovine serum (FBS), 100 U/mL penicillin, 100 μg/mL streptomycin, and 50 μM 2-mercaptoethanol. For activation, cells were stimulated for 48 h with soluble anti-CD3 (5 μg/mL) and anti-CD28 (5 μg/mL) (both from BioLegend) in the presence of 100 U/mL recombinant human IL-2 (ABclonal, Wuhan, China). Activated CD8 + T cells were then used for subsequent co-culture experiments.

### PKH26 labeling

HCT116 and DLD-1 cells were stained with the permanent cell membrane dye PKH26 according to the manufacturer’s instructions (Sigma, Livonia, Michigan, USA). PKH26 staining was analyzed using a CytoFLEX™ LX flow cytometer (Beckman Colter, Inc., Indianapolis, USA). Only when the percentage of PKH26-stained cells reached ≥98% were the cell cultures utilized for subsequent experiments.

### Cell viability assay

Cell proliferation was measured using the Cell Counting Kit-8 (CCK8, DOJINDO, Kumamoto, Japan) or the Cell Counting-Lite 3D Luminescent Cell Viability Assay (Vazyme, Nanjing, China) according to the manufacturer’s instructions. Briefly, after different treatments, 2000 cells from proliferative or dormancy-like cultures were seeded, and cell viability was measured at the indicated time points within the following 1–7 days. The cell viability assay was repeated three times with 5–8 replicates for each treatment.

### Colony formation assay

To evaluate the long-term survival and self-renewal capacity of radiation-induced dormancy-like CRC cells, a colony formation assay was performed. Proliferative HCT116 and DLD-1 cells stably expressing the fusion protein EGFP-p27K⁻ were subjected to flow cytometric sorting five days post-irradiation. The sorted radiation-induced dormancy-like populations were seeded into six-well plates and cultured for 1–2 weeks to assess clonal outgrowth. Approximately two weeks after irradiation, cell colonies were fixed, stained, photographed, and counted. The assay was performed in at least three independent experiments.

### Xenograft model establishment

Male BALB/c nu/nu mice (4–5 weeks old) were obtained from Shanghai SLAC Laboratory Animal Co., Ltd (Shanghai, China). No statistical method was used to formally predetermine the sample size for animal experiments. However, the sample size (*n* = 5–8 mice per group) was chosen based on prior experience and is consistent with widely accepted standards for xenograft studies. For the tumorigenesis assay, nude mice were randomized and subcutaneously implanted with 1 × 10^6^ proliferative or dormancy-like HCT116 and DLD-1 cells into their right flank (*n* = 5/group). After two weeks, the tumor size was measured by caliper every other day for 14 days before tumor excision and photographing. The tumor volume was calculated with the formula: tumor volume (mm^3^) = π/6×length×width^2^.

For xenograft tumor irradiation, only proliferative HCT116 and DLD-1 cells (1 × 10^6^ cells/mouse) were subcutaneously injected and monitored (*n* = 6 or 8/group). When the tumor size reached approximately 150 mm^3^, the tumors were locally irradiated with 24 Gy of X-ray at a dose rate of 2 Gy/min (X-RAD 320). Tumor growth was monitored longitudinally until sacrifice at specified time points, followed by tumor excision and immunohistochemical processing. Tumor measurements and endpoint analyses were performed by investigators blinded to the group allocation. All animal experiments were performed twice and approved by the Ethical Committee of Fudan University (2023-FSYX-05JZS). All procedures were conducted in accordance with the committee’s guidelines and regulations.

### Drug treatment

To inhibit the catalytic activity of p38MAPK, HCT116 and DLD-1 cells were incubated in the medium containing 10 μM SB203580 or SB202190 (MedChemExpress, Shanghai, China) for 2 h before harvesting. To inhibit the activity of p-AKT, the cells were treated with 3 μM WAY-600 (TargetMol, Shanghai, China) or 10 μM MK-2206 (TargetMol) for 2 h. To activate the catalytic activity of p38MAPK, HCT116 and DLD-1 cells were incubated in the medium containing 10 nM Anisomycin (TargetMol) for 24 h before harvesting. To activate p-AKT, HCT116 and DLD-1 cells were incubated in the medium containing 10 μM SC79 (TargetMol) for 24 h before harvesting. To test the protein half-life, 200 μg/ml Cyclohexamide (CHX) (MedChemExpress) was used for different treatment times to block new protein synthesis. To inhibit the activity of the proteasome, cells were treated with 10 μM MG132 (MedChemExpress) for 8 h, with 0.1% DMSO used as a control.

### Cell cycle analysis

After the indicated treatments, the proliferative or dormancy-like cells were digested into single cells, collected and fixed in 70% ethanol and stored at –20 °C for 24 h. Then the cells were washed with PBS and stained with a cell cycle reagent (Becton, Dickinson and Company, New Jersey, USA). The cell cycle distribution was detected by the flow cytometry mentioned above and analyzed with FlowJo software (version 10.8.1). Debris was excluded from the analysis to accurately quantify the percentages of cells in G0/G1, S, and G2/M phases. A minimum of 10,000 viable cell events were recorded per sample. Representative histograms show the cell cycle distribution of the gated viable cell population.

### In vitro migration assay

The cell migration assay was carried out using a 24-well Transwell® chamber (Corning) equipped with an 8 μm pore polycarbonate membrane, as previously described [21]. Regarding dormancy-like cells, HCT116 and DLD-1 cells were initially cultured as spheroids for 7 days, then dissociated into single cells and seeded into the upper inserts. For each well, the migrated cells on the bottom surface of the transwell chamber were photographed in at least 5 different fields, and the results were presented as the average number of cells per field.

### Western blot assay

Whole cell protein extracts were analyzed by western blot assay as described before [22]. The membranes were incubated with primary antibodies (Table S2) at 4 °C overnight, and then incubated with secondary antibodies for 1.5 h at room temperature. The proteins were detected by the enhanced chemiluminescence system (Millipore, Merck, Darmstadt, Germany) and their band images were recorded by the Bio-Rad ChemiDoc XRS system and analyzed using the Quantity One software (Bio-Rad Laboratories, Hercules, CA, USA).

### Quantitative real-time PCR

Total cellular RNA was isolated using the TRIzol™ Reagent (Thermo Fisher Scientific, Massachusetts, USA), then cDNA was obtained from 0.5 μg RNA for each sample using the FastKing RT Kit (TIANGEN) according to the manufacture’s instructions. Q-PCR was performed with a CFX Opus 96 real-time PCR system (Bio-Rad Laboratories) using the SuperReal PreMix Plus (SYBR Green) (TIANGEN). The PCR amplification procedure consisted of 40 cycles with 1 min of pre-denaturation and 15 s of denaturation at 95 °C, and 1 min of annealing/extension at 60 °C. The gene-specific primers for *DDX21*, *CDKN1A (p21)*, *NUCKS1, HERC2* and *ACTB* are shown in the Table S3.

### Co-immunoprecipitation (Co-IP) assay

CRC cells were lysed in a lysis buffer containing protease inhibitors (Beyotime Biotechnology, Shanghai, China). The whole cell lysates were collected and incubated with 5 µg of anti-DDX21 antibody (Santa Cruz), or 5 µg of anti-HERC2 antibody (Santa Cruz), or 5 µg of anti-IgG antibody (Beyotime) overnight at 4 °C. The next day, they were incubated with Dynabeads Protein G beads (Invitrogen, California, USA) for 2 h at 4°C. After washing four times with immunoprecipitation lysis buffer, the beads were resuspended in 2× SDS loading buffer (Beyotime) and boiled at 95°C for 5 min. The eluted samples were then subjected to Western blot analysis.

### Immunohistochemistry and immunofluorescence staining

For Paraffin-embedded tissue sections of CRC xenografts, endogenous peroxidase activity was quenched first, and then the sections were stained with the Ki67 kit (Becton), bound with a primary antibody at a dilution of 1:100 at 4°C overnight and an anti-mouse secondary antibody (1 h at room temperature), and visualization was achieved using DAB chromogen. IHC score measurements and statistical analyses were performed by investigators blinded to the group allocation.

Immunofluorescence assays of Ki67 and DDX21 were carried out as previously described [23]. Cells were dispersed with Accutase (Thermo), fixed with 4% paraformaldehyde for 15 min, and permeabilized with the enhanced immunostaining permeabilization buffer (Beyotime) for 5 min at room temperature. The samples were incubated with anti-Ki67 (ProteinTech) or anti-DDX21 (Santa Cruz) at a dilution of 1:200 at 4 °C overnight, and subsequently incubated with secondary antibody of anti-rabbit IgG Alexa Fluor^®^488 or anti-mouse IgG Alexa Fluor^®^488 (Cell Signaling Technology) at 1:1000 in the dark for 2 h. The cell nuclei were counterstained with DAPI (5 µg/ml) (Beyotime) for 5 min. The tissue and cell images of at least 5 fields were randomly captured using a Leica DMi8 inverted microscope (Leica Microsystems GmbH, Wetzlar, Germany) or a high-content imaging system (ImageXpress Micro 4) (Molecular Devices LLC, San Jose, CA, USA).

### RNA interference

Small interfering RNA (siRNA) duplexes targeting *DDX21*, *p21*, and *NUCKS1* were obtained from GenePharma (Suzhou, China). The negative control RNA duplex (NC) for siRNAs was designed to be nonhomologous to any human genome sequence. Cells were transfected with 40 nM siRNA using Lipofectamine™ RNAiMAX (Thermo Fisher Scientific) for 24 h according to the manufacturer’s instructions. The efficiency of siRNA knockdown was verified using a western blot assay 48 h after transfection. The sequences of the small interfering RNA are presented in the Table S4.

### Plasmid and transfection

Using the coding sequence (CDS) of *DDX21* as a template, a series of DDX21-truncated fragments corresponding to amino acid residues 1-180, 180-573 and 573-783 were amplified by PCR and cloned into a Flag-tagged destination vector containing a CMV promoter and a puromycin resistance marker. Full-length human DDX21 and NUCKS1 cDNAs were cloned into the pcDNA3.1(+) vector (Invitrogen) for overexpression studies. The HA-ubiquitin wild-type and mutant (K48R, K63R) plasmids were generated by inserting the corresponding full-length human ubiquitin sequences into the HA-pcDNA3.1(+) vector. All plasmids were obtained from QEgene Biotechnology (Shanghai, China) and verified by DNA sequencing. Transfections were performed using Hieff Trans Liposomal Transfection Reagent (Yeasen Biotechnology, Shanghai, China) according to the manufacturer’s protocols. The plasmid maps of DDX21 and NUCKS1 overexpression are presented in Supplementary Fig. S7.

### Dual-luciferase reporter assay

The NUCKS1 promoter sequence was cloned into the pGLS-basic vector to generate the firefly luciferase reporter plasmid. A Renilla luciferase plasmid was used as an internal control. Both plasmids were constructed by HANBIO (Shanghai, China). For the assay, 293 T cells were seeded in 24-well plates and co-transfected with the reporter and control plasmids. Twenty-four h post-transfection, cells were transfected with either a non-targeting control siRNA (si-NC) or a DDX21-targeting siRNA (si-DDX21). After an additional 48 h, firefly and Renilla luciferase activities were assessed using the Dual-Lumi^TM^ Luciferase Reporter Gene Assay Kit (Beyotime) according to the manufacturer’s instructions. Briefly, cells were lysed in the reporter cell lysate buffer, and 100 µL of each lysate was transferred to a 96-well plate. Firefly luciferase activity was measured first by injecting 100 µL of the detection reagent and recording the relative light units (RLU). Subsequently, 100 µL of the Renilla luciferase assay working solution was added to the same well to measure Renilla luciferase activity. The firefly luciferase RLU values were normalized to the Renilla luciferase RLU values for each sample. The normalized luciferase activity in the si-DDX21 group was compared to that of the si-NC group to determine the relative effect of DDX21 knockdown on NUCKS1 promoter activity.

### Transfection of lentivirus

We designed and transduced a probe, using a fusion protein between the fluorescent protein EGFP and a p27K^−^ mutant lacking CDK inhibitory activity (EGFP-p27K^−^) into HCT116 and DLD-1 cells. The cells expressing EGFP-p27K^−^ were in the G0 and early G1 phases. The EGFP-p27K gene sequence was inserted into the pCDH-CMV-MCS-EF1 lentiviral vector (Ruinan Biotechnology, China). To obtain stably transfected CRC cells, HCT116 and DLD-1 cells were infected with the above lentivirus and screened with 4 µg/ml puromycin (Beyotime). The efficiency of EGFP-p27K^-^ in CRC cells was monitored by immunofluorescence assay.

### Quantitative proteomic analysis of Tandem Mass Tag (TMT) and bioinformatics analysis

The protein profiles of proliferative and dormancy-like HCT116 cells were assessed by Shanghai Genechem Co., Ltd. using liquid chromatography in combination with tandem mass spectroscopy (LC-MS/MS). Gene Ontology (GO) pathway analysis of the identified proteins was conducted using Blast2GO [24]. For transcriptomic analysis, tumor dormancy-related datasets GSE48995, GSE193500 and GSE64262 were downloaded from the Gene Expression Omnibus (GEO) database and analyzed with the online tool GEO2R, with RNA-seq data normalized using the DESeq2 method. An online Venn diagram tool (http://www.bioinformatics.com.cn/static/others/jvenn/example.html) [25] was used to identify overlapping genes.

Gene Set Enrichment Analysis (GSEA) was performed using GSEA software (v4.1.0, https://www.gsea-msigdb.org, accessed Feb 24, 2022), and nominal *P* values were calculated by 1,000 phenotype permutations. Multiple hypothesis testing was controlled by estimating the False Discovery Rate (FDR) using the Benjamini-Hochberg method. Gene sets with FDR < 0.25 were considered statistically significant, as recommended by the GSEA developers.

DDX21 expression levels in normal, primary tumor, and metastatic colorectal cancer tissues were analyzed using the TNMplot database (https://tnmplot.com/analysis/, accessed March 6, 2026). This web-based tool integrates transcriptomic data from The Cancer Genome Atlas (TCGA), Genotype-Tissue Expression (GTEx), and Gene Expression Omnibus (GEO) repositories, enabling simultaneous comparison across tissue types. Statistical significance was determined using the platform’s built-in analysis tools, with *P* < 0.05 considered statistically significant.

The Gene Expression Profiling Interactive Analysis (GEPIA2) database (http://gepia.cancer-pku.cn/index.html, accessed Dec 1st, 2022), an interactive web portal for gene expression analysis based on TCGA and GTEx data, including 9736 tumors and 8587 normal samples, was utilized to generate the overall survival curve based on the gene expression with the Kaplan–Meier method and Log-Rank test in colorectal cancer.

Publicly available datasets from the Genomic Data Commons (GDC) (https://portal.gdc.cancer.gov/, accessed 20 Dec, 2024) were used to investigate the correlation between DDX21 and PD-L1 (CD274) expression in colorectal cancer.

### Ubiquitination assay

CRC cells were induced into a dormancy-like state for 48 h, then MG132 (MedChemExpress) was added to the medium at a final concentration of 10 µM and incubated for an additional 8 h. To identify ubiquitinated DDX21, proteins in the cell lysate were immunoprecipitated using an anti-DDX21 antibody (Santa Cruz) followed by detection with an anti-Ubi antibody (Cell Signaling Technology) via western blot assay.

### Molecular Docking analysis

The three-dimensional structures of DDX21 (PDB ID: 6L5O) and HERC2 (PDB ID: 3KCI) were obtained from the Protein Data Bank (PDB) (https://www.rcsb.org). Molecular docking simulations were performed using the ZDOCK module [26]. (v3.0.2, https://zdock.wenglab.org, accessed 20 January 2025) to investigate potential interaction modes between DDX21 and HERC2, with an angular step size of 4 Å. The resulting docking configurations were visualized and analyzed with the PyMOL Molecular Graphics System (version 2.4).

### Statistical analysis

All cellular experiments, except for TMT proteomics, were performed with at least three independent biological replicates, with the exact sample size (n) for each group indicated in the figure legends. Data are presented as mean ± standard deviation (SD). Comparisons between two groups were analyzed using a two-tailed Student’s t test. For multiple group comparisons, one-way analysis of variance (ANOVA) was used. All statistical tests were two-sided unless otherwise specified. Western blot band intensities were quantified using ImageJ software. Immunofluorescence and immunohistochemistry images were analyzed for the percentage of positive cells from at least five randomly selected fields per sample using Image J. Patient overall survival was analyzed using the Kaplan–Meier method, and differences between groups were assessed by the log-rank test. All statistical analyses and graphing were performed using GraphPad Prism 9 (GraphPad Software, USA). *P* value < 0.05 was considered statistically significant (*P < 0.05, **P < 0.01, ***P < 0.001).

## Results

### Dormancy-like phenotype endows colorectal cancer cells with radio-resistance and increases PD-L1 expression

Dormancy-like cells can be identified as label-retaining cells by PKH26 dye staining due to their sustained dye retention over time [27]. Using this method, we enriched dormancy-like cells from HCT116 and DLD-1 colon cancer spheroids. It was found that a high proportion of PKH26-positive cells could be maintained in dormant cultures for up to two weeks. In contrast, PKH26 signals were nearly undetectable within three days in regularly cultured proliferative CRC cells (Fig. S1A, B). Moreover, the growth rate of two dormancy-like CRC cell lines was significantly reduced compared to their proliferative counterparts (Fig. S1C). Analysis of cell cycle distribution showed a substantial increase in the G0/G1 population in dormancy-like cells, rising from 60.9% to 89.6% in HCT116 cells and from 39.6% to 62.4% in DLD-1 cells, relative to their proliferative counterparts (Fig. S1D). The induced dormancy-like state was reversible, as re-stimulation with serum and re-plating the cells in standard medium restored proliferative capacity (Data not shown).

**Fig. 1:**
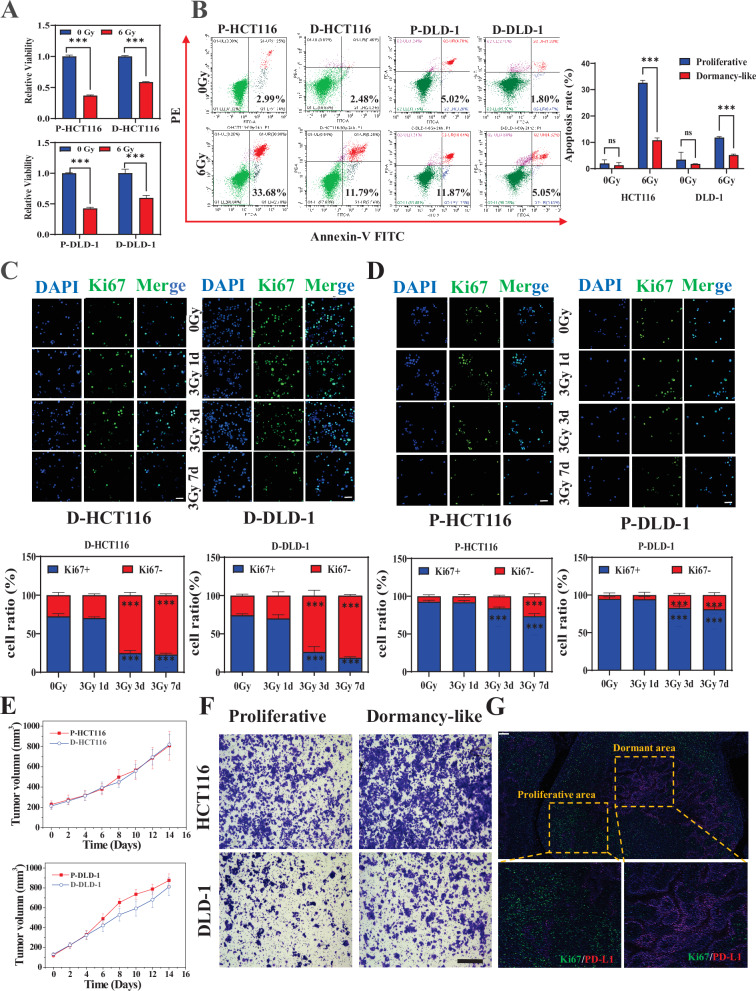
Dormancy-like state confers radio-resistance to colorectal cancer cells and increases PD-L1 expression. **A** Relative cell viabilities of the proliferative and dormancy-like CRC cells after 6 Gy irradiation. **B** Apoptosis rates of the proliferative and dormancy-like CRC cells before and after 6 Gy irradiation. **C** Representative immunofluorescence images of Ki67 staining of the dormancy-like HCT116 and DLD-1 cells digested from the cell spheroid and cultured 1-7 days after 3 Gy irradiation. Column plots show the percentage of Ki67-positive or negative cells at the indicated time points. Scale bar, 50 μm. The *P* values indicate the statistical significance relative to the cell ratios of the 0 Gy group. **D** Representative immunofluorescence staining of Ki67 of the proliferative HCT116 and DLD-1 cells at the indicated time points after 3 Gy irradiation. Column plots show the percentage of Ki67-positive or Ki67-negative cells. Scale bar, 50 μm. **E** The growth curves of xenograft tumor of HCT116 (top) and DLD-1 (bottom) (D/P indicates the cells at dormancy-like (D) or proliferative (P) state when implanted into mice), (*n* = 5 for each group). **F** Representative images of the migration assay of the dormancy-like CRC cells compared with their proliferative counterparts. Scale bar, 500 μm. **G** Representative immunofluorescence images of Ki67 staining and PD-L1 staining of the CT26 tumor. Scale bar, 200 μm. Data are presented as mean ± SD from three biologically independent experiments (*n* = 3). Statistical significance was determined using a two-tailed unpaired Student’s t test or one-way ANOVA with Tukey’s post hoc test. *P < 0.05, **P < 0.01, *** P < 0.001.

Dormant cancer cells have been reported to be resistant to chemotherapy [14]. This has prompted us to investigate whether these cells are also resistant to radiotherapy. As shown in Fig. 1A, B, after X-ray radiation, dormancy-like HCT116 and DLD-1 cells had significantly higher viability and lower apoptosis rates compared to their proliferative counterparts, indicating enhanced radio-resistance in dormancy-like CRC cells. Ki67, a well-established marker of proliferation, had an inverse correlation with cellular dormancy. On day 3 and day 7 after radiation, the proportion of Ki67-negative cells in the dormancy-like populations increased 2- to 3-fold relative to non-irradiated controls (Fig. 1C). A similar increase in Ki67-negative cells was also observed in the proliferative HCT116 and DLD-1 populations (Fig. 1D). In addition, we labeled proliferative HCT116 and DLD-1 cells with PKH26 before radiation, and found that the irradiated cells exhibited a significantly higher proportion of PKH26-positive cells compared to non-irradiated controls (Fig. S2A), indicating that irradiation increases the non-proliferating cell population. Irradiation can induce transient cell cycle arrest [28]. which shares certain features with dormancy. To directly distinguish radiation-induced dormancy from transient arrest (predominantly manifests as G2/M phase arrest), the fusion protein EGFP-p27K^-^ was stably transfected into HCT116 and DLD-1 cells, thereby labeling dormancy-like cells exclusively during the G0 phase with green fluorescence [29]. The results revealed a progressive increase in the dormancy-like cell population beginning at day 1 after irradiation, with statistically significant differences observed by day 3 (Fig. S2B), demonstrating that the majority of irradiation-induced non-proliferating cells are G0/G1-arrested, dormancy-like cells. To functionally validate these findings, radiation-induced dormancy-like CRC cells were harvested and sorted by flow cytometric sorting 5 days post-irradiation (Fig. S2C) and then subjected to a colony formation assay. It is shown that these cells retained the ability to form colonies even after extended culture (Fig. S2D). These results confirm that the radiation-induced dormancy-like phenotype observed in this study represents a stable, non-proliferative state distinct from transient cell cycle arrest. Furthermore, although local irradiation with 24 Gy significantly inhibited the growth of HCT116 and DLD-1 xenografts (Fig. S1E), the number of dormancy-like (Ki67-negative) CRC cells in tumor tissues gradually increased from day 1 to day 7 post-irradiation (Fig. S1F). Collectively, these results demonstrate that dormancy-like CRC cells are radioresistant and that radiation promotes the transition of CRC cells into a dormancy-like state, both in vitro and in vivo.

Previous studies have demonstrated that dormant tumor cells have the capabilities of tumor-initiating and metastasis-initiating [30]. To assess the tumor formation ability of dormancy-like CRC cells, we employed a xenograft mouse model. Equal numbers of dormancy-like or proliferative cells (1×10^6^ cells per mouse) were subcutaneously implanted into the right flank of mice. All mice developed tumors, and no significant difference in tumor growth was observed between the two groups (Fig. 1E). Although both groups exhibited similar tumorigenic capacity in this acute engraftment model, dormancy-like CRC cells displayed a significantly enhanced migratory potential upon re-cultivation under standard conditions (Figs. 1F and S1G), suggesting that a prior dormancy-like state may prime cells for a more aggressive phenotype upon reactivation. Additionally, immunofluorescence analysis revealed a marked upregulation of PD-L1 expression in the tumor dormant area compared to the proliferative area (Fig. 1G). Taken together, these findings provide preliminary evidence that dormancy-like CRC cells may acquire malignant phenotypic features with potential implications for radio-resistance.

### DDX21 is identified as a candidate gene for regulating dormancy-like state, which may be associated with a poor prognosis in colorectal cancer

**Fig. 2:**
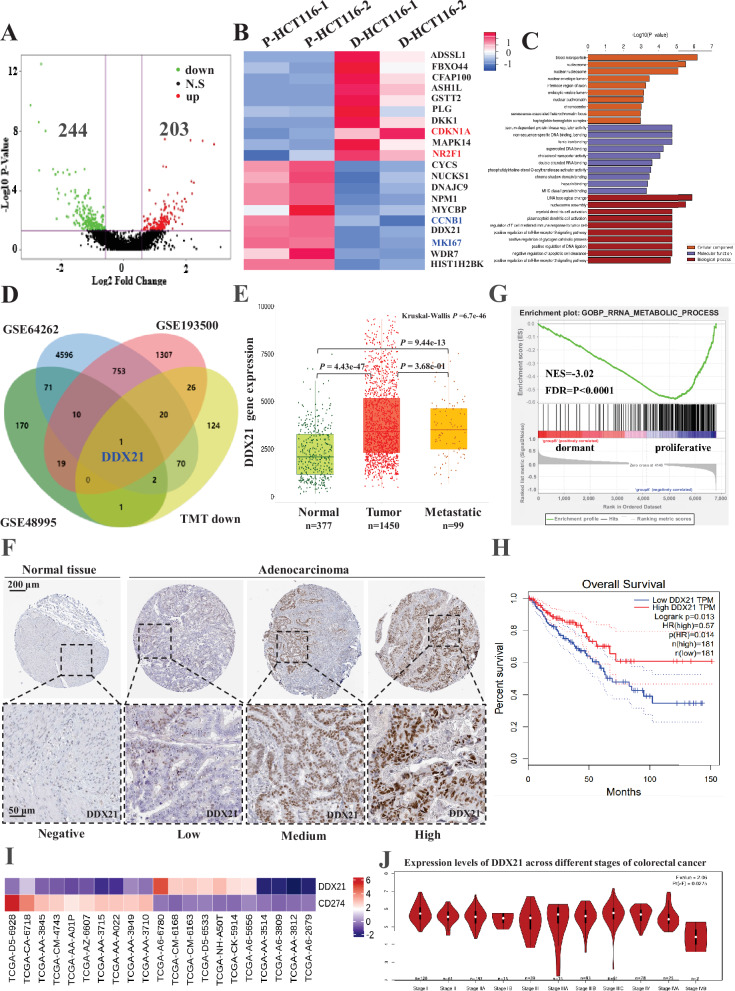
Identification of DDX21 as a dormancy-like modulation candidate gene. **A** Symmetric scatter diagram of differentially expressed genes between proliferative and dormancy-like HCT116 cells based on TMT data. *P* values were adjusted for multiple comparisons using the Benjamini-Hochberg method. Significantly changed genes (|log2FC | > 1, FDR < 0.05) are highlighted in red or green. **B** Heatmap of the DEGs in the proliferative and dormancy-like HCT116 cells. (D/P indicates the cells at dormancy-like or proliferative state). **C** GO pathway analysis of genes significantly up- or down-regulated in dormancy-like HCT116 cells. **D** Venn diagram of the overlap genes among the datasets from GSE48995, GSE46242, GSE193500 and the down-regulated DEGs in dormancy-like HCT116 TMT data. **E** DDX21 expression levels in normal colon tissues (*n* = 377), primary colorectal cancer (*n* = 1450), and metastatic lesions (*n* = 99). Data were obtained from the TNMplot database. Scatter plots show individual samples. Box plots show median, interquartile ranges (Q1–Q3), and range. Statistical significance was determined using the platform’s built-in analysis tools. ***P < 0.001 versus normal. **F** Representative immunohistochemical images of DDX21 staining in normal colorectal tissue and adenocarcinomas based on the Human Protein Atlas website. Scale bar (above), 200 μm. Scale bar (below), 50 μm. **G** GSEA analysis of the enrichment of rRNA metabolism process gene set in the down-regulated genes of dormancy-like HCT116 cells. **H** Kaplan–Meier curves of the survival of CRC patients based on the expression level of DDX21 according to TCGA and GEPIA datasets. **I** Heatmap of the expression of DDX21 and CD274 in colorectal cancer patients based on the Genomic Data Commons datasets. **J** Violin plot of the expression levels of DDX21 across different stages of colorectal cancer based on the GEPIA2 database (http://gepia2.cancer-pku.cn/). The violin shape represents the kernel density estimation of the expression distribution. The thick black bar indicates the interquartile range (IQR). The white dot indicates the median. *n* = 120 (Stage I), 61 (Stage II), 197 (Stage IIA), 15 (Stage IIB), 39 (Stage III), 15 (Stage IIIA), 93 (Stage IIIB), 61 (Stage IIIC), 78 (Stage IV), 25 (Stage IVA), 2 (Stage IVB) biologically independent tumor samples. Statistical significance was assessed using one-way ANOVA, with the F-statistic and exact *P* value (Pr(>F)) reported in the figure.

To identify candidate genes involved in the regulation of the dormancy-like state in CRC cells, we performed TMT-based quantitative proteomics analysis on protein extracts derived from dormancy-like and proliferative HCT116 cells. The analysis identified 447 differentially expressed proteins, with 203 upregulated and 244 downregulated, in dormancy-like cells compared to proliferative cells (Fig. 2A). The TMT results confirmed the upregulation of known dormancy markers, including NR2F1 [31]. and p21 (CDKN1A), in dormancy-like HCT116 cells. In contrast, proliferation markers such as Ki67 and cyclin B (CCNB1) were significantly downregulated (Fig. 2B), thereby validating the reliability and robustness of the TMT assay. Gene ontology (GO) analysis showed that the majority of differentially expressed genes (DEGs) were localized to the nucleus and predominantly associated with biological processes including DNA topology modulation, nucleosome assembly, bone marrow dendritic cell activation, tumor cell immune response, and DNA connection (Fig. 2C). To further explore these findings, tumor dormancy-related datasets (GSE48995, GSE46262 and GSE193500) were integrated with the downregulated DEGs identified from the TMT analysis. Venn diagram analysis indicated that DDX21 was the sole gene overlapping across these datasets (Fig. 2D).

DDX21 is widely recognized as a regulator of rRNA processing and is predominantly localized within the nucleus. Analyses of datasets from the TNMplot database revealed that DDX21 expression is elevated in both primary colorectal tumors and metastatic lesions compared to normal mucosal tissues (Fig. 2E). Consistently, data from the Human Protein Atlas showed higher DDX21 expression in CRC tumor tissues versus normal controls (Fig. 2F). Gene set enrichment analysis (GSEA) showed significant enrichment of rRNA metabolic process-related genes in the lower expressed dataset of dormancy-like HCT116 (Fig. 2G), which is consistent with the function of DDX21 and further confirmed our hypothesis that DDX21 may be a candidate gene regulating dormancy-like phenotypes. To further explore the correlation between the function of DDX21 and its clinical significance, we analyzed the overall survival of colorectal cancer patients using the TCGA datasets through the GEPIA web platform. Notably, low DDX21 expression was correlated with poor prognosis in colorectal cancer patients (Fig. 2H). According to the Genomic Data Commons (GDC) datasets, there was a negative correlation between DDX21 and CD274 (PD-L1) expression (Fig. 2I). In particular, DDX21 expression level significantly decreased in advanced tumor stage (Stage IVB) of colorectal cancer patients (Fig. 2J). Collectively, these results indicate that down-regulation of DDX21 may be a key event in inducing cancer cells into a dormancy-like state, and it may promote malignant progression and poor clinical outcomes in CRC, especially in advanced stages.

### Down-regulation of DDX21 induces CRC cells into a dormancy-like state

**Fig. 3:**
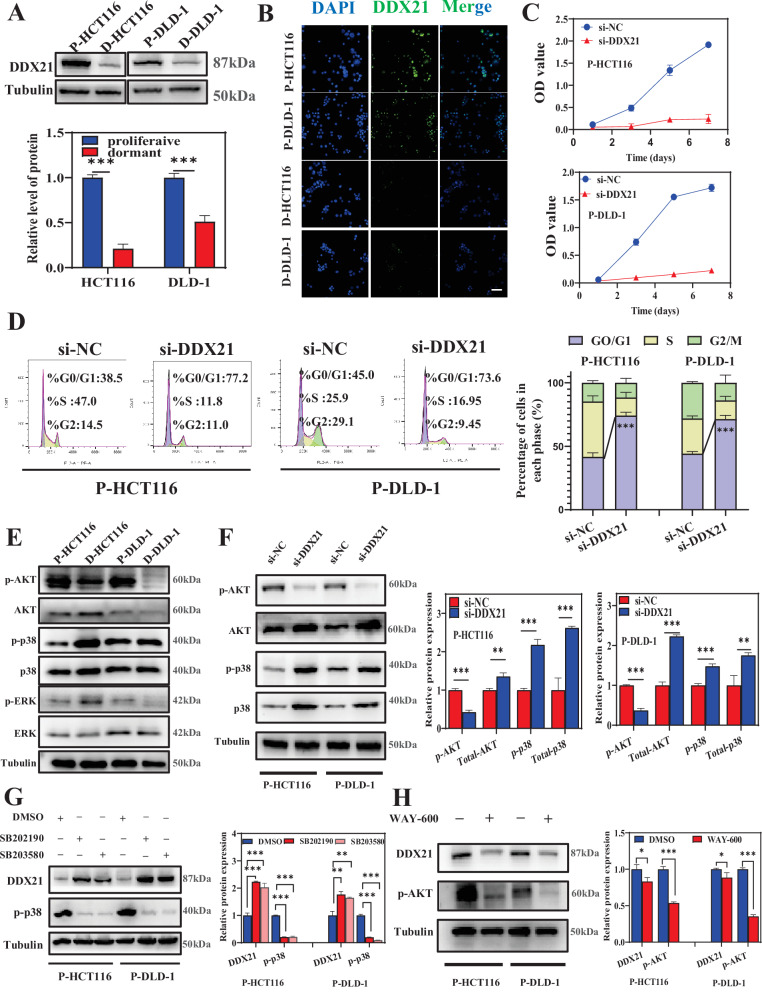
Low expression of DDX21 induces CRC cells into a dormancy-like state through the AKT/p38MAPK pathway. **A** Western blot assay of DDX21 protein in the proliferative or dormancy-like CRC cells (above) and its relative levels (below). **B** Representative immunofluorescence staining of DDX21 in the proliferative and dormancy-like CRC cells. Scale bar, 50 μm. **C** Cellular growth curves of the proliferative HCT116 (above) and DLD-1 (below) cells transfected with DDX21 siRNA. **D** Cell cycle analysis of the proliferative CRC cells with DDX21 siRNA transfection. *P* values indicate the statistical significance relative to the percentages of the G0/G1 phase. **E** Western blot assay of p-AKT, AKT, p-ERK, ERK, p-p38, and p38 proteins in the proliferative or dormancy-like CRC cells. **F** Western blot assay of p-AKT, total-AKT, p-p38, and total-p38 of the proliferative CRC cells transfected with si-DDX21. **G** Western blot assay of p-p38 and DDX21 of the proliferative CRC cells treated with SB202190 or SB203580. **H** Western blot assay of p-AKT and DDX21 of the CRC cells treated with WAP-600. D/P indicates the cells at dormancy-like or proliferative state. Data are presented as mean ± SD from three biologically independent experiments (*n* = 3). Statistical significance was determined using two-tailed unpaired Student’s *t* test. *P < 0.05, **P < 0.01, *** P < 0.001.

Given the above results suggesting a role for low DDX21 expression in promoting cancer cell dormancy and poor prognosis, we then assessed DDX21 protein levels in two colorectal cancer cell lines using Western blotting and immunofluorescence. The results confirmed that DDX21 was markedly downregulated in dormancy-like cells (Fig. 3A, B). To further investigate the function of DDX21 in CRC cell dormancy, we performed siRNA-mediated knockdown in proliferative HCT116 and DLD-1 cells (Fig. S3A). As expected, DDX21 depletion completely suppressed cell proliferation (Fig. 3C) without inducing apoptosis (Fig. S3B). Meanwhile, silencing DDX21 led to a significant down-regulation of cyclin D1, a key regulator of G1/S transition (Fig. S3C), resulting in a pronounced G0/G1 cell cycle arrest (Fig. 3D). To specifically label and track dormancy-like cells, we employed HCT116 and DLD-1 cells stably expressing the fusion protein EGFP-p27K^-^. Both immunofluorescence (Fig. S3D) and flow cytometry (Fig. S3E) demonstrated that DDX21 knockdown significantly increased the proportion of G0/G1-arrested (EGFP-positive) cells. Collectively, these findings supported the point that down-regulation of DDX21 could induce CRC cells into a dormancy-like state.

Previous studies have shown that increased p38MAPK activity is a characteristic of dormant cancer cells [32]. Consistent with this, we observed a significant increase in the ratio of phosphorylated p38 (p-p38)/phosphorylated ERK (p-ERK) in dormancy-like CRC cells, along with a marked reduction in AKT phosphorylation (Ser473) compared to proliferative CRC cells (Figs. 3E and S3F). Similarly, silencing DDX21 yielded the same results (Fig. 3F). Interestingly, although p-p38 was scarcely detected in the nuclear lysates of both proliferative and dormancy-like CRC cells, it was strongly expressed in the cytoplasm of dormancy-like cells (Fig. S3G). A possible reason for this could be the p38MAPK nuclear-to-cytoplasmic translocation under specific conditions [33]. Meanwhile, inhibition of p38 activity with SB202190 or SB203580 substantially increased DDX21 expression (Fig. 3G), while suppression of p-AKT with WAY-600 decreased DDX21 levels in both cell lines (Fig. 3H). Notably, we found that inhibition of p38MAPK could also reverse the upregulation of p27 in dormancy-like CRC cells (Fig. S3H), a cyclin-related protein associated with G0 phase arrest, and enhanced the sensitivity of two dormancy-like cell lines to 6 Gy of X-ray irradiation (Fig. S3I). Gain- and loss-of-function experiments further demonstrated that treatment with a p-p38 inhibitor (SB203580) or a p-AKT activator (SC79) partially reversed the dormancy-like phenotypes induced by DDX21 knockdown in proliferative CRC cells. This was validated by restored Ki67 positivity, reduced p27 expression, and reversal of G0/G1 phase arrest (Fig. S4A–D). Conversely, direct activation of p-p38 (using anisomycin) or inhibition of p-AKT (using MK-2206) in proliferative CRC cells recapitulated the dormancy-like phenotype observed upon DDX21 knockdown, as characterized by elevated p21 and p27 protein levels (Fig. S4E, F). Collectively, these findings demonstrate that DDX21 downregulation can induce a dormancy-like state in CRC cells via the classic p38MAPK/AKT signaling pathway, and further suggest a reciprocal regulatory relationship within this axis in maintaining the dormancy-like phenotype.

### DDX21 down-regulation promotes CRC cells into a dormancy-like state through the DDX21-NUCKS1-p27/p21 axis

**Fig. 4:**
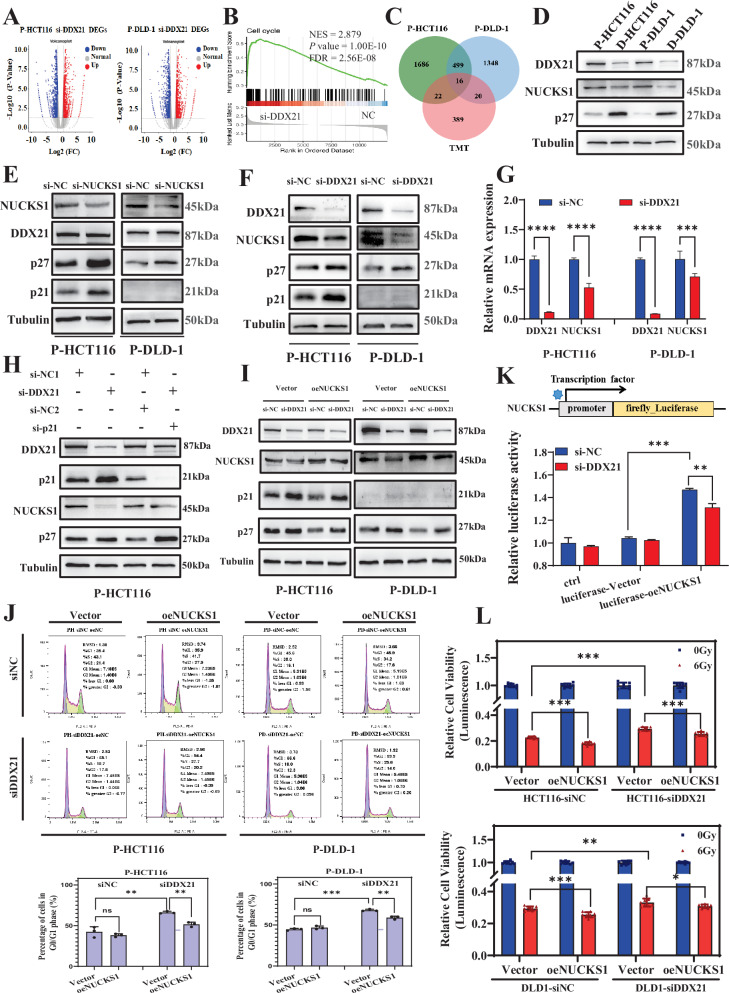
Low expression of DDX21 induces CRC cells into a dormancy-like state through the DDX21-NUCKS1-p27/p21 axis. **A** Symmetric scatter diagram of DEGs between si-NC and si-DDX21 in the proliferative HCT116 (left) and DLD-1 (right) cells based on RNA-seq data. *P* values were adjusted for multiple comparisons using the Benjamini-Hochberg method. Significantly changed genes (|log2FC|> 1, FDR < 0.05) are highlighted in red or blue. **B** GSEA analysis of the enrichment of the cell cycle gene set in the DDX21 knockdown HCT116 and DLD-1 cells. Analysis was performed on RNA-seq data. Normalized Enrichment Score (NES), nominal *P* value (*P*), and False Discovery Rate (FDR adjusted by Benjamini-Hochberg procedure) are shown. Gene sets with FDR < 0.25 were considered significantly enriched. **C** Venn diagram of DDX21-related dormant genes in the CRC cells sorted from the DEGs of the proliferative HCT116 cells with si-DDX21 (green), proliferative DLD-1 cells with si-DDX21 (blue) and dormant-related TMT data (pink). **D** Western blot assay of DDX21, NUCKS1, and p27 proteins in the proliferative and dormancy-like CRC cells. **E** Western blot assay of NUCKS1, DDX21, p21, and p27 proteins in si-NC and si-NUCKS1 CRC proliferative cells. **F** Western blot assay of NUCKS1, DDX21, p21, and p27 proteins in si-DDX21 CRC proliferative cells. **G** RT-qPCR assay of *DDX21* and *NUCKS1* mRNA expressions in si-DDX21 CRC proliferative cells. **H** Western blot assay of DDX21, NUCKS1, p21 and p27 proteins in proliferative HCT116 cells before and after knockdown DDX21 and p21. **I** Western blot assay of DDX21, NUCKS1 and dormant-related proteins in si-DDX21 CRC proliferative cells before and after overexpression of NUCKS1 or empty vector. **J** Cell cycle analysis of the proliferative CRC cells with DDX21 siRNA transfection before and after overexpression of NUCKS1 or empty vector. *P* values indicate the statistical significance relative to the percentages of G0/G1 phase. **K** Relative luciferase activity of NUCKS1 with si-NC or si-DDX21 in 293 T cells. **L** Relative cell viability of proliferative CRC cells with DDX21 siRNA transfection before and after overexpression of NUCKS1 or empty vector, followed by 6 Gy irradiation. D/P indicates the cells in a dormancy-like or proliferative state. Data are presented as mean ± SD from at least three biologically independent experiments. Statistical significance was determined using a two-tailed unpaired Student’s *t* test. *P < 0.05, **P < 0.01, *** P < 0.001, **** P < 0.0001.

To uncover the molecular mechanisms underlying DDX21-mediated regulation of dormancy-like phenotypes, we performed RNA-sequencing analysis in two proliferative CRC cell lines after DDX21 knockdown. Through comparative transcriptomic profiling, 515 DEGs (|Fold change|> 2.0, *P* < 0.05) were identified upon DDX21 depletion (Fig. 4A). In line with our hypothesis, the cell cycle-related pathway was significantly enriched in the DDX21 knockdown cells, as evidenced by the GSEA analysis (Fig. 4B). Then, the RNA-seq data were integrated with TMT-based proteomic data, leading to the identification of 16 differentially expressed candidates. Among them, NUCKS1 exhibited the most significant down-regulation (Fig. 4C and Table S5). NUCKS1, a recognized substrate for both nuclear casein kinase and cyclin-dependent kinase, plays a crucial role in cell cycle regulation, especially during the G1/S transition [34]. Based on these findings, we hypothesized that NUCKS1 might act as a key mediator in DDX21-associated dormancy-like phenotypes.

In accordance with the expression profile of DDX21, NUCKS1 was found to be notably downregulated in dormancy-like cells when compared to their proliferative counterparts (Figs. 4D and S5A). Knockdown of NUCKS1 in proliferative HCT116 cells led to a substantial increase in both p27 and p21 protein levels. Although p21 was not detectable in DLD-1 cells, a significant upregulation of p27 was similarly observed (Figs. 4E and S5B). Furthermore, depletion of NUCKS1 induced G0/G1 phase arrest and increased the proportion of EGFP-positive cells (Fig. S5C–E). These findings indicate that NUCKS1 regulates dormancy-like phenotype by modulating the cell cycle through p21/p27-dependent mechanisms.

Intriguingly, knockdown of NUCKS1 had no effect on DDX21 expression (Fig. 4E). However, silencing DDX21 significantly reduced both the NUCKS1 protein level (Figs. 4F and S5F) and mRNA level (Fig. 4G) in proliferative cells, suggesting that DDX21 acts upstream of NUCKS1 in regulating the transition of the cell cycle into a dormancy-like state. Consistent with this, DDX21 knockdown markedly upregulated the expression of p21 and p27 in HCT116 cells and p27 in DLD-1 cells (Fig. 4F). Since the expression of p21 protein was hardly detectable in DLD-1 cells, to verify the regulatory role of DDX21 on p21/p27 in both CRC cell lines, we successively inhibited the expression of DDX21 and p21 in HCT116 cells. As expected, the upregulation of p27 induced by DDX21 siRNA was further enhanced after p21 knockdown (Figs. 4H and S5G). Collectively, these findings demonstrate that down-regulation of DDX21 induces a dormancy-like state by increasing the expression of p21/p27, leading to G0/G1 phase arrest, with p27 partially compensating for the loss of p21 in DLD-1 cells.

To further elucidate the regulatory relationship between NUCKS1 and DDX21, we overexpressed NUCKS1 in both HCT116 and DLD-1 cells (Fig. S5H), followed by DDX21 knockdown. Strikingly, NUCKS1 overexpression significantly attenuated the dormancy-like phenotypes induced by DDX21 knockdown, impairing the upregulation of p21/p27 and G0/G1 phase arrest (Figs. 4I, J and S5I). Moreover, DDX21 knockdown markedly reduced the activity of a NUCKS1-luciferase reporter, suggesting that NUCKS1 is a direct transcriptional or post-transcriptional target of DDX21 (Fig. 4K). Functionally, NUCKS1 overexpression significantly enhanced the sensitivity of DDX21-knockdown cells to 6 Gy X-ray irradiation (Fig. 4L). Taken together, these findings establish DDX21 as an upstream regulator of NUCKS1 and demonstrate that DDX21 downregulation drives cell cycle arrest and promotes a dormancy-like phenotype in CRC cells via the DDX21-NUCKS1-p21/p27 axis.

### DDX21 in dormancy-like CRC cells is degraded via the ubiquitin-proteasome pathway

**Fig. 5:**
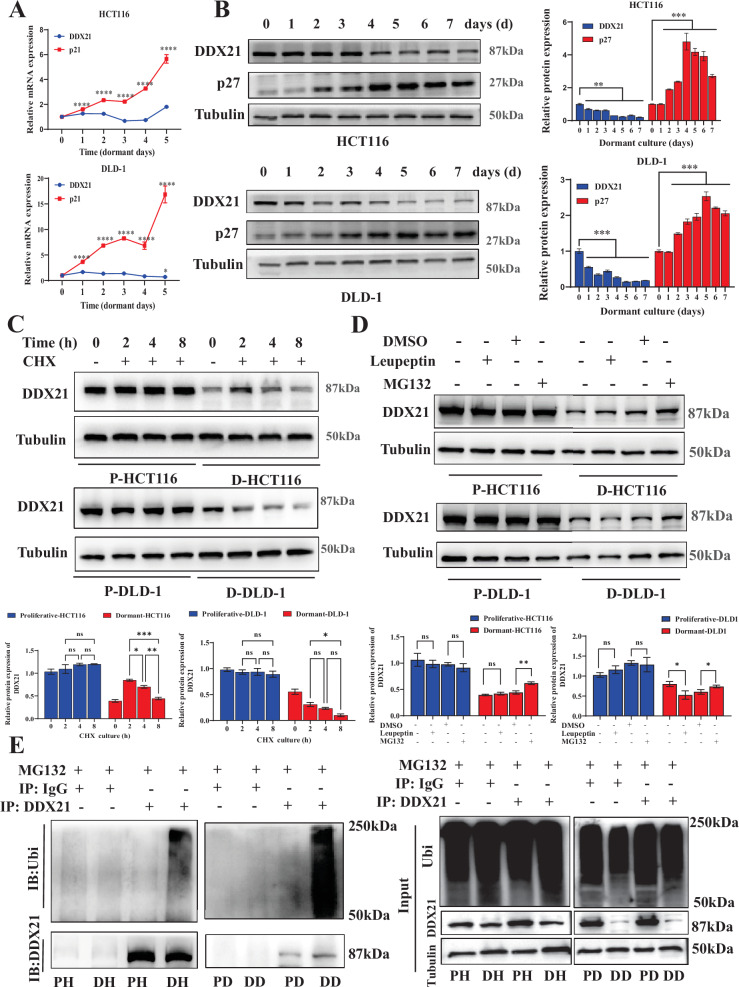
DDX21 in dormancy-like cells was degraded via the ubiquitin-proteasome pathway. **A** RT-qPCR analysis of p21 and DDX21 expressions in the HCT116 (above) and DLD-1 (below) cells at different time points during dormancy-like induction. **B** Western blot assay of DDX21 and p27 proteins in the HCT116 (above) and DLD-1 (below) cells at different time points during dormancy-like induction. **C** Western blot assay of DDX21 protein in the proliferative and dormancy-like HCT116 (above) and DLD-1 (below) cells treated with cycloheximide (CHX) for the indicated time. **D** Western blot assay of DDX21 protein in the proliferative and dormancy-like HCT116 (above) and DLD-1 (below) cells with or without the treatment of MG132 or Leupeptin. **E** Western blot assay of the ubiquitination of DDX21 in the proliferative and dormancy-like CRC cells. D/P indicates the cells in a dormancy-like or proliferative state. Data are presented as mean ± SD from three biologically independent experiments (*n* = 3). Statistical significance was determined using a two-tailed unpaired Student’s *t* test. *P < 0.05, **P < 0.01, *** P < 0.001, **** P < 0.0001.

To investigate how DDX21 is downregulated in dormancy-like CRC cells, we examined the expression dynamics of DDX21 during the dormancy process. Interestingly, DDX21 mRNA abundance showed no significant reduction within 5 days post-dormancy induction, but its protein levels exhibited a time-dependent decrease. Meanwhile, the expression levels of p21 and p27 continuously increased over time (Fig. 5A, B). This indicates that the decrease of DDX21 in dormancy-like CRC cells is mediated by post-translational modifications rather than transcriptional regulation.

Protein expression can be regulated via multiple mechanisms, including autophagy [35]. midnolin-proteasome pathways [36]. and ubiquitin-proteasome systems [37]. In this study, treatment with cycloheximide (CHX), which inhibits new protein synthesis, significantly decreased the half-life of the DDX21 protein in dormancy-like CRC cells compared to the proliferative cells (Fig. 5C). Inhibiting the 26S proteasome with MG132 reversed the reduction of DDX21 protein levels in the dormancy-like CRC cells whereas the lysosome inhibitor leupeptin had no impact (Fig. 5D). Consistent with these findings, ubiquitination signals associated with DDX21 were significantly higher in dormancy-like cells than in proliferative counterparts (Fig. 5E). These results together demonstrate that the down-regulation of DDX21 in dormancy-like cells is mediated by ubiquitin-proteasome-dependent degradation.

### DDX21 degradation in dormancy-like cells is promoted by HERC2-mediated K48 and K63-linked polyubiquitination chains

**Fig. 6:**
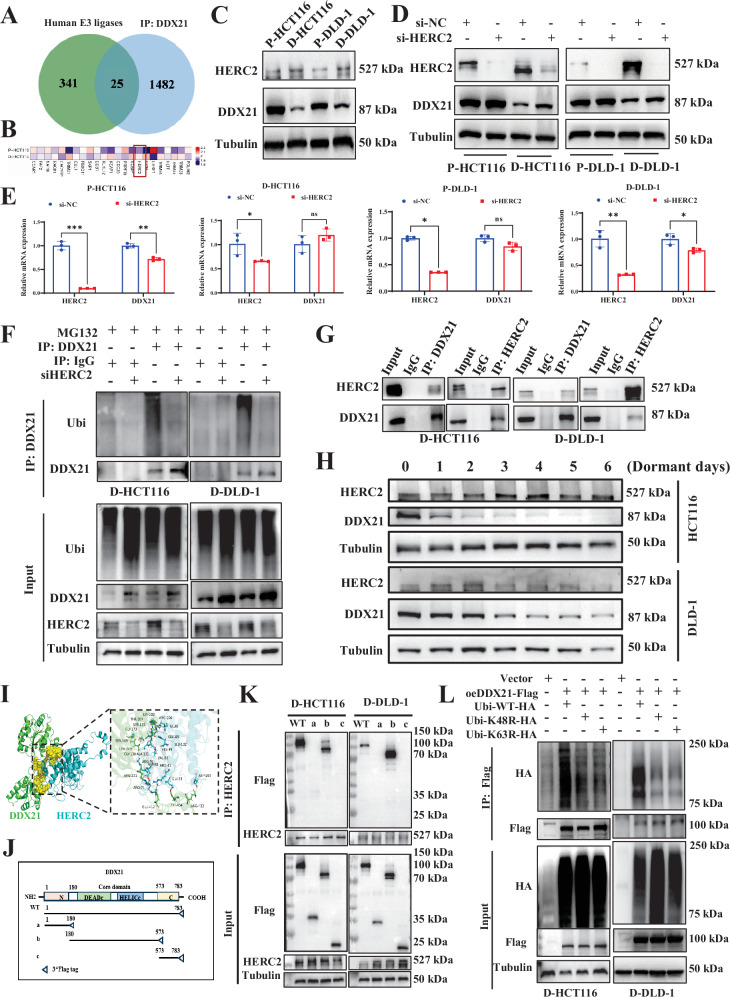
DDX21 degradation in dormancy-like cells is promoted by HERC2-mediated K48 and K63-linked polyubiquitination chains. **A** Venn diagram of the overlap proteins between the known human E3 ubiquitin ligases (green) and DDX21- immunoprecipitated proteins in dormancy-like HCT116 cells (blue). **B** Heatmap of the expression of overlapped E3 ubiquitin ligases in the proliferative (above) and dormancy-like (below) HCT116 cells based on the TMT dataset. **C** Western blot assay of HERC2 and DDX21 proteins in proliferative and dormancy-like CRC cells. **D** Western blot assay of HERC2 and DDX21 proteins in HERC2-KD proliferative and dormancy-like CRC cells. **E** RT-qPCR analysis of *HERC2* and *DDX21* expressions in HERC2-KD proliferative and dormancy-like CRC cells. **F** Western blot assay of the ubiquitination of DDX21 in the HERC2-KD dormancy-like CRC cells. **G** Co-immunoprecipitation and Western blot assay of HERC2 and DDX21 proteins in the whole cell lysates of dormancy-like CRC cells. **H** Western blot assay of DDX21 and HERC2 proteins in the HCT116 (above) and DLD-1 (below) cells at different time points during dormancy-like induction. **I** Surface diagram of the docking model and their interface residues between HERC2 and DDX21 protein (HERC2, blue; DDX21, green). **J** Schematic representation of three flag-fused DDX21 constructs containing amino acids 1–180, 180–573 and 573–783. **K** Co-immunoprecipitation and Western blot assay of HERC2 and different DDX21 domains in the whole cell lysates of dormancy-like CRC cells. **L** Anti-HA immunoblotting assay of DDX21 polyubiquitination in dormancy-like CRC cells transfected with wild-type or K48/K63 mutant Ubiquitin. D/P indicates the cells in a dormancy-like or proliferative state. Data are presented as mean ± SD from three biologically independent experiments (*n* = 3). Statistical significance was determined using two-tailed unpaired Student’s *t* test. *P < 0.05, **P < 0.01, *** P < 0.001.

We then explored the specific E3 ubiquitin ligase that mediates DDX21 ubiquitination in dormancy-like CRC cells. Using co-immunoprecipitation from dormancy-like HCT116 cell lysates, we captured DDX21-associated proteins and overlapped them with the reference E3 ligase list from UbiBrowser. This comparison identified 25 overlapping E3 ligases (Fig. 6A). We further analyzed the expression of these candidates in a dormancy-like cell TMT dataset and generated a corresponding heatmap (Fig. 6B). Given that DDX21 is a nuclear protein, its E3 ligase is likely to be located in the nucleus. Among them, we noticed HERC2, a nuclear protein that has been widely reported as an E3 ubiquitin-protein ligase [38]. Subsequently, we confirmed that HERC2 was upregulated in dormancy-like CRC cells and that its expression was inversely correlated with that of DDX21 (Figs. 6C and S6A). To further investigate the influence of HERC2 on DDX21, we silenced HERC2 expression in both CRC proliferative and dormancy-like cells. The results indicated that DDX21 expression increased significantly after HERC2 silencing, especially in dormancy-like CRC cells (Figs. 6D and S6B). Meanwhile, HERC2-knockdown (HERC2-KD) failed to induce significant upregulation of DDX21 at the transcriptional level, suggesting the observed protein accumulation is not transcriptionally mediated (Fig. 6E).

To determine whether DDX21 degradation is mediated by HERC2-dependent ubiquitination, we first examined ubiquitination levels in HERC2-KD dormancy-like CRC cells. As expected, we observed significantly reduced DDX21 ubiquitination signals in HERC2-KD dormancy-like cells (Fig. 6F), demonstrating that HERC2 plays an essential role in DDX21 ubiquitination. Meanwhile, HERC2 silencing attenuated the cell cycle arrest in dormancy-like CRC cells (Fig. S6C), indicating that HERC2 knockdown facilitates cell cycle re-entry from the dormancy-like state. Then immunoprecipitation assays were carried out to investigate whether HERC2 directly binds to DDX21. Interestingly, the specific mutual binding of HERC2 and DDX21 was only observed in dormancy-like CRC cells. (Figs. 6G and S6D). To test whether this binding is specific to dormancy-like conditions or merely a consequence of elevated HERC2 protein levels, endogenous HERC2 and DDX21 protein levels were investigated over time. The results revealed a time-dependent downregulation of DDX21, the rate and magnitude of which were markedly greater than those of HERC2 upregulation (Fig. 6H). Furthermore, the addition of proteasome inhibitor MG132 led to an accumulation of HERC2 protein in proliferative cells, yet no binding of HERC2 with DDX21 was detected under these conditions (Fig. S6E). These results suggest that the direct HERC2-DDX21 interaction was dependent on dormancy-inducing conditions, rather than HERC2 abundance alone.

The interaction was further evaluated by the ZDOCK model, and the surface analysis showed that domain 187-412 of DDX21 might interact with HERC2 via several hydrogen bonds (Fig. 6I), thus confirming the stability of the interaction between DDX21 and HERC2 in dormancy-like CRC cells. Subsequently, DDX21 functional truncation constructs were generated (a: 1-180aa, NTD; b: 180-573aa, Core helicase domain; c: 573-783aa, CTD), together with the full-length DDX21 (WT), to determine the specific domain interacting with HERC2 (Fig. 6J). Consistent with the predicted results, only the core helicase domain of DDX21 (construct b) interacts with HERC2 (Fig. 6K), verifying the conclusion that HERC2 interacts with the core helicase domain of DDX21 to induce its ubiquitination-mediated degradation in the dormancy-like CRC cells.

Next, we aimed to characterize the ubiquitin linkage types involved in DDX21 ubiquitination. Since poly-ubiquitination chains can form through eight lysine residues (K6, K11, K27, K29, K33, K48, K63, or Met1), with K48- and K63-linked chains being the most prevalent [39]. We employed ubiquitin mutants (K48R and K63R) to block these specific linkages. Interestingly, we found that both K48R and K63R mutants could partly reduce DDX21 polyubiquitination in dormancy-like cells (Fig. 6L). This implies a complex ubiquitination pattern that may involve multiple chain types in the regulation of DDX21 stability during dormancy-like induction.

### Low expression of DDX21 facilitates immune evasion and radio-resistance in colorectal cancer

**Fig. 7:**
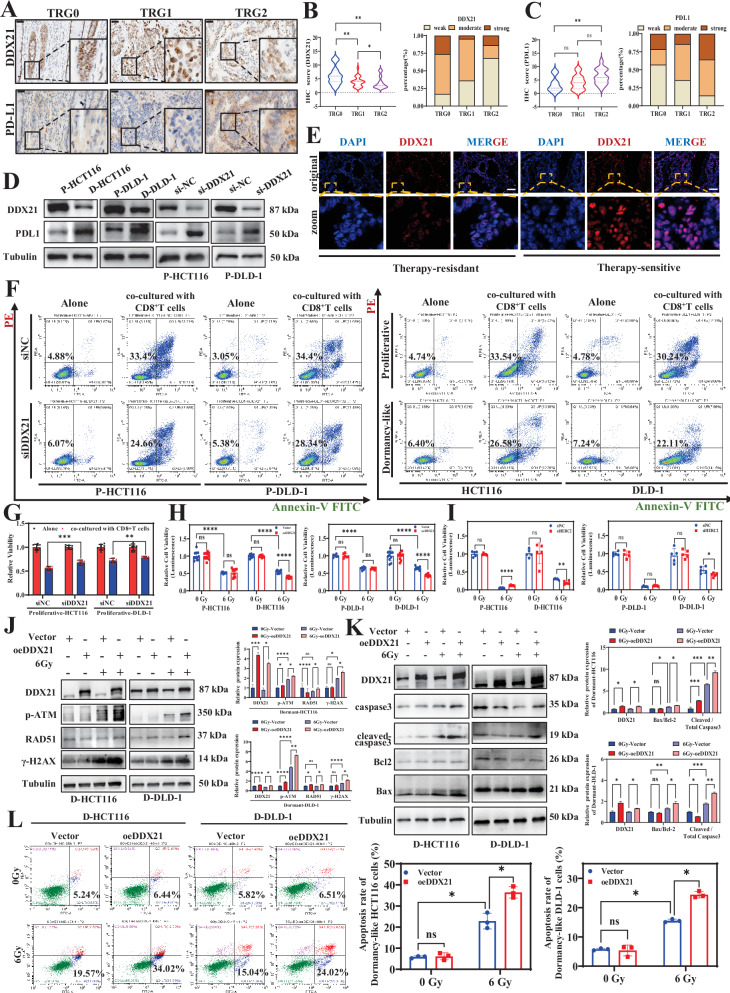
Low expression of DDX21 promotes immune evasion and radio-resistance in colorectal cancer. **A** Representative immunohistochemical images of DDX21 and PD-L1 staining in colorectal cancer tissue samples of different tumor regression grade (TRG) (*n* = 10). Scale bars, 50 μm. **B, C** Immunohistochemistry scores and staining intensities percentages of DDX21 and PD-L1 in colorectal cancer tissue samples of different TRG. **D** Western blot assay of PD-L1 and DDX21 in proliferative, dormancy-like CRC cells, and DDX21-KD proliferative CRC cells. **E** Representative immunofluorescence images of DDX21 expression in the tumor tissues of a therapy-resistant CRC patient and a therapy-sensitive CRC patient. Nuclei were stained with DAPI. Scale bars, 100 μm. **F** Apoptosis rates of proliferative CRC cells with or without DDX21 knockdown (left), and of proliferative versus dormancy-like CRC cells (right), following co-culture with activated CD8⁺ T cells for 48 h. **G** Relative viability of proliferative CRC cells with or without DDX21 knockdown, assessed by CCK-8 assay after 48 h of co-culture with activated CD8⁺ T cells. **H** Relative cell viabilities of proliferative and dormancy-like CRC cells transfected with DDX21 overexpression plasmid or empty vector following 6 Gy irradiation. **I** Relative cell viabilities of proliferative and dormancy-like HERC2-KD CRC cells following 6 Gy irradiation. **J** Western blot assay of DDX21, p-ATM, RAD51 and γ-H2AX in dormancy-like CRC cells transfected with DDX21 overexpression plasmid or empty vector before and after 6 Gy irradiation. **K** Western blot assay of DDX21, caspase3, cleaved caspase3, Bax and Bcl2 in dormancy-like CRC cells transfected with DDX21 overexpression plasmid or empty vector before and after 6 Gy irradiation. **L** Apoptosis rates of dormancy-like CRC cells transfected with DDX21 overexpression plasmid or empty vector before and after 6 Gy irradiation. D/P indicates the cells in a dormancy-like or proliferative state. Data are presented as mean ± SD from three biologically independent experiments (*n* = 3). Statistical significance was determined using two-tailed unpaired Student’s *t* test. *P < 0.05, **P < 0.01, *** P < 0.001, **** P < 0.0001.

Dormant tumor cells are widely known for their resistance to various therapies [40]. To investigate whether DDX21 serves as a viable biomarker for this dormancy-like population, we evaluated its expression in primary colorectal cancer specimens via immunohistochemistry, assessing its association with dormancy and clinical prognosis. The results showed a significant negative correlation between DDX21 expression and tumor regression grades (TRG) (Fig. 7A). With tumor progression, DDX21 staining in CRC cells was impaired, and the proportion of DDX21-positive cells also decreased (Fig. 7B). Conversely, PD-L1, a marker of immune evasion, was positively correlated with CRC TRG (Fig. 7A, C).

To explore the relationship between DDX21 and PD-L1, we examined PD-L1 expression levels in dormancy-like CRC cells and DDX21-knockdown (DDX21-KD) CRC cells (Figs. 7D and S6F, G). The results showed that both dormancy-like and DDX21-KD cells displayed increased PD-L1 expression, indicating that low DDX21 expression not only induces a dormancy-like state but also promotes immune evasion in CRC cells. Additionally, the analysis of a pair of CRC clinical samples revealed that DDX21 expression was significantly lower in therapy-resistant sample tissues compared to therapy-sensitive tissues (Fig. 7E). Functional assays revealed that after 48 h of co-culture with activated CD8 + T cells isolated from human peripheral blood, DDX21-KD and dormancy-like CRC cells exhibited a significantly reduced apoptosis rate (Fig. 7F) and enhanced cell viability (Fig. 7G) compared to their respective control. Morphological assessment further confirmed that DDX21-KD and dormancy-like CRC cells maintained better cellular integrity under co-culture conditions (Fig. S6H). These results suggested that DDX21 downregulation could recapitulate the immune-resistant phenotype observed in dormancy-like cells. We have demonstrated that NUCKS1 functions downstream of DDX21. Notably, while DDX21 knockdown led to PD-L1 upregulation, this effect was markedly attenuated by concomitant NUCKS1 overexpression (Fig. S6I). Collectively, these results suggest that DDX21 may contribute to immune evasion in dormancy-like CRC cells, at least partially by upregulation of PD-L1 through a NUCKS1-dependent pattern.

To assess the effect of DDX21 on the radiosensitivity of CRC cells, we compared the viability of empty vector cells and DDX21-overexpressing cells following exposure to 6 Gy of irradiation using the Cell Counting-Lite 3D Luminescent Cell Viability Assay. The results showed that DDX21 overexpression specifically enhanced radiosensitivity in dormancy-like CRC cells, rather than in proliferative CRC cells (Fig. 7H). At the same time, silencing the DDX21-related E3 enzyme HERC2 notably enhanced the radiosensitivity in CRC dormancy-like cells (Fig. 7I). Western blot analysis further verified that DDX21 overexpression specifically inhibited the expression of cell cycle arrest proteins in dormancy-like CRC cells (Fig. S6J). Meanwhile, irradiation significantly increased the expression of DNA damage markers, such as p-ATM and γ-H2AX, in DDX21-overexpressing dormancy-like CRC cells (Fig. 7J). Moreover, the Bax/Bcl-2 ratio and Cleaved caspase-3/Total caspase-3 levels were elevated in DDX21-overexpressing dormancy-like CRC cells after irradiation (Fig. 7K), which was consistent with the results of flow cytometry apoptosis assays (Fig. 7L). Taken together, these findings collectively demonstrate that DDX21 overexpression significantly enhances the radiosensitivity of dormancy-like CRC cells, thus further supporting its functional role in promoting dormancy-like state and the consequent immune evasion and radio-resistance in colorectal cancer cells.

## Discussion

Cancer cell dormancy is increasingly recognized as a fundamental mechanism that contributes to therapeutic resistance and recurrence [41]. posing significant clinical challenges to the effective treatment of CRC [42]. Despite advancements in the study of tumor dormancy across various cancer types [43–45]. The underlying molecular mechanisms of CRC dormancy remain poorly understood. Our study confirmed the malignant potentials of CRC dormancy-like cells, including their radio-resistance both in vitro and in vivo, enhanced migratory capacity upon reactivation, and significant up-regulation of PD-L1. Most importantly, we highlighted a significant association between the reduced DDX21 expression and the above characteristics of dormancy-like cells. Based on the TCGA colorectal cancer cohort, we observed that low DDX21 expression was significantly correlated with reduced overall survival, and combined with our own clinical patient samples, we further revealed that low DDX21 levels were associated with poor treatment response and elevated PD-L1. These findings position DDX21 as a promising biomarker for assessing CRC malignancy and predicting the therapeutic response to radiotherapy or immunotherapy. Therefore, therapeutic strategies targeting dormant CRC cells or modulating their dormant characteristics may represent a promising approach to improving patient outcomes.

DDX21, an RNA helicase involved in transcription and rRNA metabolism, is highly expressed in various malignancies and promotes proliferation and transcriptional activity in colorectal cancer and other tumor types [46–48]. In this study, we found that the downregulation of DDX21 is a critical event in dormancy-like induction. When DDX21 was silenced in HCT116 and DLD-1, many dormancy characteristics were exhibited, including G0/G1 phase arrest, growth inhibition, decreased cyclin D1, and elevated p21/p27 expression. This is consistent with the study of other CRC cell lines such as SW480, SW460 and HCT8, where the down-regulation of DDX21 was verified to suppress cell proliferation and cell cycle development [49]. Meanwhile, in endothelial cells and HUVEC cells, the loss of DDX21 could also lead to an accumulation of G0/G1 phase arrest [50]. Importantly, our analysis revealed the downregulation of DDX21 expression during CRC progression. Particularly, pronounced reduction was observed in stage IV tumors compared to earlier disease stages. This finding is consistent with previous studies in colorectal cancer by Tanaka, A et al., which demonstrated that decreased DDX21 expression in advanced-stage or specific molecular subtypes correlates with enhanced tumor aggressiveness and increased malignant potential [51]. Furthermore, our analysis revealed that decreased DDX21 expression is associated with poorer prognosis in colorectal cancer patients, consistent with observations in glioma and renal cell carcinoma cohorts from the Human Protein Atlas database. Likewise, DDX21 downregulation has also been shown to correlate with increased metastatic potential and unfavorable clinical outcomes in breast cancer patients [52]. Our findings suggested that DDX21 downregulation could serve as a biomarker for a dormancy-like state in colorectal cancer cells, similar to the role of NR2F1 in identifying dormant breast cancer cells and predicting bone metastasis risk [53]. From a therapeutic perspective, although direct clinical translation remains speculative, the identification of the DDX21-NUCKS1-p21/p27 axis provides a conceptual framework for future investigation into the regulation of tumor cell dormancy. Whether this axis can be exploited to therapeutically modulate dormancy and prevent recurrence will require further investigation in appropriate preclinical models.

Additionally, we confirmed that PD-L1 expression is upregulated in both dormancy-like CRC cells and DDX21-KD CRC cells, suggesting the immune evasion potential of dormancy-like cells. As an immune checkpoint molecule, PD-L1 functions as a transmembrane protein that interacts with programmed cell death protein 1 (PD-1) on T cells, leading to the inhibition of T cell activation and subsequent tumor immune escape [54]. Accumulating evidence indicates that PD-L1 is markedly upregulated in CRC tissues compared to normal counterparts, and its overexpression is clinically associated with advanced tumor stage, lymph node metastasis, and vascular invasion [55]. In accordance with our results, dormant breast cancer cells suppress T cell function through PD-L1 upregulation. And the transcriptional signature of dormant breast cells includes the coordinated expression of PD-L1 and other immune checkpoint genes, suggesting an intrinsic immunosuppressive program in these dormant tumor cells [56]. Similarly, a study of dormant esophageal cancer stem cells showed that dormant cells can escape immune elimination by enhancing PD-L1 signaling and suppressing CD8^+^T cell infiltration [57]. In this study, our analysis of clinical colorectal cancer data revealed a significant inverse correlation between DDX21 and PD-L1 expression. We found that PD-L1 levels exhibited a marked elevation with the progression of tumor regression grade in colorectal cancer. Collectively, our findings indicate that dormancy-like CRC cells may enhance immune evasion by downregulating DDX21, which subsequently upregulates immune evasion-related molecules such as PD-L1, thereby impacting clinical therapeutic efficacy.

The AKT and p38MAPK pathways are the most studied and regarded as classic dormant pathways [58]. Our research confirmed the involvement of the AKT and p38MAPK pathways in dormancy-like induction in CRC cells and revealed that suppressing DDX21 can effectively promote these classic dormant pathways, subsequently inducing dormancy-like phenotypes. This finding is in line with prior research showing that low p-AKT expression or reduced AKT activity is associated with metastatic dormancy in breast cancer [59]. and quiescence in ovarian cancer spheroids [60]. In accordance with our results, P38MAPK activation has also been reported to contribute to cellular and tumor dormancy in prostate cancer [32] and breast cancer [61]. Interestingly, we discovered a negative feedback loop between p-p38 signaling and DDX21 expression. To the best of our knowledge, little literature has reported the interaction between DDX21 and the p38MAPK pathway. More importantly, we demonstrate that inhibiting p38MAPK in dormancy-like CRC cells overcomes their radio-resistance and may enhance the efficacy of radiotherapy.

Meanwhile, it is important to note that the effect of radiotherapy on tumor dormancy is highly context-dependent. Our findings show that radiation can induce a dormancy-like state in colorectal cancer cells, as evidenced by an increased proportion of Ki67-negative cells. This observation aligns with a study reporting that ionizing radiation induces sustained cell cycle arrest through activation of the ATM–Chk2–p21 axis [62]. This effect is further enhanced by microenvironmental cues such as hypoxia, which has been shown to induce G1 phase accumulation and thereby confer a radioprotective phenotype that persists even upon reoxygenation [63]. On the other hand, IR-induced tissue damage and subsequent inflammation can create an awakening-permissive niche that triggers dormant cell reactivation. Key pro-inflammatory cytokines released after irradiation, including IL-6, G-CSF, and IL-17A, have been shown to cooperatively disrupt dormancy in breast cancer models [64]. These factors recruit and polarize M2-type tumor-associated macrophages, which secrete EGFR ligands that directly activate ERK/MAPK signaling and drive cell cycle re-entry in dormant cancer cells [65]. Additionally, IR-induced angiogenesis and extracellular matrix remodeling, such as osteoclast-mediated bone matrix degradation, releasing TGF-β and IGF-1, can further contribute to dormancy exit [66]. A recent study by Ridwan et al. demonstrated that in a mouse model of intracerebral melanoma, radiation-induced dormancy could be either shortened or prolonged by subsequent endotoxin-induced inflammation, depending on the inflammatory context [67]. This duality underscores the critical role of the tumor microenvironment, immune status, and inflammatory signals in determining the fate of dormant cells after radiation exposure. A comprehensive understanding of these opposing effects will be essential for developing therapeutic strategies that reliably promote durable dormancy and prevent recurrence.

Notably, we revealed that the DDX21-NUCKS1-p27/p21 axis regulates dormancy-like state in CRC cells, representing a previously unrecognized mechanism. NUCKS1 is involved in a number of biological processes, such as DNA damage response [68]. energy metabolism, and tumor development [69]. Recently, it was reported that NUCKS1 promoted cells into S phase and facilitated the degradation of p21/p27 [34]. Here, we demonstrated the involvement of NUCKS1 in inducing a dormancy-like state of CRC cells and identified that NUCKS1 was a direct downstream target of DDX21. Its overexpression alleviated the DDX21 knockdown-induced upregulation of p21, p27, and PD-L1. Meanwhile, DDX21 can directly bind to the promoter region and regulate NUCKS1 at the transcriptional level. In previous studies, DDX21 has been demonstrated to bind to the promoter regions of Pol II-transcribed genes, indicating its direct involvement in transcription pre-initiation complex assembly and transcriptional regulation [16]. For example, DDX21 could occupy the MCM5 gene locus and induce MCM5 expression [70]. in CRC cells, and it could be recruited to the promoter of Snail in MCF-7 cells [52]. Collectively, these findings explained how DDX21 induces and sustains dormancy-like CRC cells in the G0/G1 phase of the cell cycle.

Last but not least, we elucidated how DDX21 was down-regulated in dormancy-like CRC cells. It is very interesting that the decrease in DDX21 was not due to mRNA expression modulation but was mediated by HERC2-induced ubiquitination. There are several reports indicating that the expression of DDX21 can be modulated by post-translational mechanisms [71, 72]. HERC2 belongs to the large HERC family, which has been implicated in various physiological processes, including membrane trafficking, immune response, DNA repair, and cancer biology [73]. Previous studies have shown that HERC2, as a nuclear E3 ubiquitin ligase, could mediate the ubiquitination of diverse substrates [74]. and mediate the radiosensitivity [20]. For instance, by interacting with RNF8, HERC2 could facilitate the formation of the HERC2-MDC1-RNF8 ternary complex, thus coordinating the ubiquitin-dependent assembly of DNA repair factors on damaged chromosomes and promoting the DNA repair [19]. Consistently, our study reveals that HERC2 binds to the core domain of DDX21 in dormancy-like CRC cells, mediating DDX21 ubiquitination and thereby inducing dormancy-like phenotypes and enhancing cellular radio-resistance. The molecular architecture of DDX21 consists of three main domains: an N-terminal domain (NTD), a central helicase core domain (HCD), and a C-terminal domain (CTD). The NTD has not been functionally characterized. The CTD facilitates RNA G-quadruplex recognition and binding, while the HCD mediates both RNA unwinding and recognition, representing the signature functional module of this protein family [75]. Here, we identified that HERC2 directly binds to the DDX21 helicase core domain, and specifically, this interaction is only observed in dormancy-like CRC cells and is absent in their proliferative counterparts. This differential interaction pattern appears to be primarily mediated by the dormancy-like condition, rather than merely resulting from elevated HERC2 protein levels. Under certain conditions, when dormant signals and pathways are activated in CRC cells, HERC2 is upregulated and enriched to interact with DDX21 and induces its subsequent ubiquitin-mediated degradation. Notably, our experiments also demonstrated that HERC2 knockdown significantly enhanced radiosensitivity in dormancy-like CRC cells, which provides functional evidence that HERC2 regulates the DDX21-mediated dormancy process and its associated phenotypic traits.

There are still limitations to this study. First, the low-adhesion serum-free culture method employed in this study is widely recognized as a reliable and established approach for modeling dormancy-like states in vitro [76, 77]. The findings acquired using the in vitro model should not be directly equated with genuine tumor dormancy in vivo, as they lack the effect of the tumor micro-environmental factors. Second, the reliance on established cell lines represents another inherent limitation, as these models may not fully recapitulate the complex dormancy dynamics and therapeutic responses of patient tumors. Therefore, colorectal cancer patient-derived organoids (PDOs) may offer a promising approach to investigate these phenomena in more clinically relevant systems [78]. Furthermore, our study establishes HERC2 as a key E3 ligase targeting DDX21 for proteasomal degradation in dormancy-like CRC cells. However, the upstream signals that trigger HERC2 upregulation during dormancy-like induction remain an open question. We speculate that the unique stresses of the dormant niche, such as the imperative for protein homeostasis remodeling and the accumulation of oxidative stress, are plausible inducers of HERC2 expression [38, 79]. Testing this hypothesis and identifying the specific signaling pathways involved will be a vital goal for future studies.

**Fig. 8:**
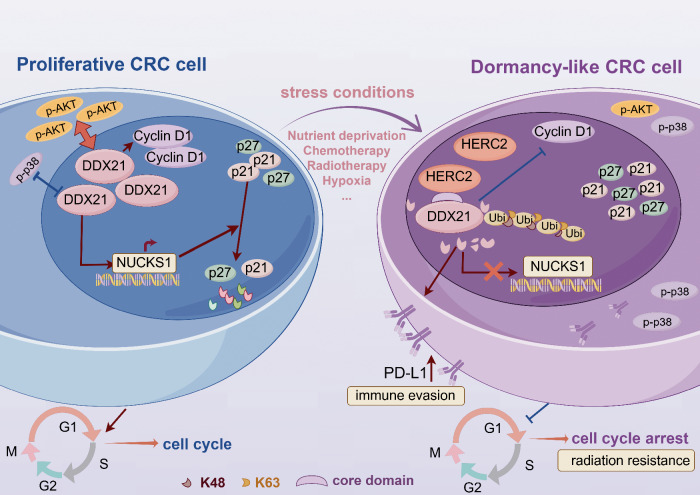
Schematic model of dormancy-like state induced by DDX21-NUCKS1-p27/p21 axis in CRC cells. Under severe stress conditions such as serum-free suspension culture, colorectal cancer (CRC) cells undergo DDX21 protein degradation, which triggers a dormancy-like state. This dormancy-associated DDX21 depletion results in PD-L1 upregulation and enhanced therapy resistance. In dormancy-like CRC cells, HERC2 protein accumulates and binds to the core domain of DDX21, promoting its ubiquitination and subsequent degradation through K48- and K63-linked polyubiquitin chain formation. DDX21 could bind to the promoter region of NUCKS1 and mediate the degradation of downstream p21 and p27 proteins. Therefore, in dormancy-like CRC cells, downregulation of DDX21 suppresses CyclinD1 and NUCKS1 expression while increasing p21 and p27 accumulation, ultimately blocking G0/G1 to S phase transition and inducing dormancy-like phenotypes. Meanwhile, DDX21 expression is reciprocally regulated by phosphorylation cascades and canonical dormancy-associated pathways, exhibiting an inverse relationship with p-p38 MAPK activity while demonstrating a positive correlation with AKT phosphorylation status. This figure was created using Figdraw (https://www.figdraw.com).

In summary, this study discovers that DDX21 serves as a central regulator of a dormancy-like state, which confers therapy-resistance in CRC cells (Fig. 8). By binding to the core domain of DDX21, HERC2 mediates the ubiquitination degradation of DDX21, which then activates the p38MAPK pathway and inhibits AKT phosphorylation. Additionally, the ubiquitination of DDX21 suppresses NUCKS1 transcription and impairs the degradation of p21/p27, ultimately resulting in G0/G1 phase arrest. From the above two perspectives, low-expressed DDX21 promotes CRC cells into a dormancy-like state. Overall, these findings provide novel insights into the mechanisms underlying dormancy-like state and therapy-resistance of CRC cells, establishing DDX21 as a promising target for monitoring dormancy-like cells and predicting therapy efficacy in clinical practice.

## Supplementary information

Supplementary Figures and Tables

Uncropped Western blots

Reproducibility checklist

## Data Availability

Data presented in this study are available on request from the corresponding author upon reasonable request.

## References

[1] Bray F, Ferlay J, Soerjomataram I, Siegel RL, Torre LA, Jemal A. Global cancer statistics 2018: GLOBOCAN estimates of incidence and mortality worldwide for 36 cancers in 185 countries. CA Cancer J Clin. 2018;68:394–424. 30207593 10.3322/caac.21492

[2] Dekker E, Tanis PJ, Vleugels JLA, Kasi PM, Wallace MB. Colorectal cancer. Lancet. 2019;394:1467–80. 31631858 10.1016/S0140-6736(19)32319-0

[3] Basu S, Dong Y, Kumar R, Jeter C, Tang DG. Slow-cycling (dormant) cancer cells in therapy resistance, cancer relapse and metastasis. Semin Cancer Biol. 2022;78:90–103. 33979674 10.1016/j.semcancer.2021.04.021 PMC8576068

[4] Risson E, Nobre AR, Maguer-Satta V, Aguirre-Ghiso JA. The current paradigm and challenges ahead for the dormancy of disseminated tumor cells. Nat Cancer. 2020;1:672–80. 33681821 10.1038/s43018-020-0088-5 PMC7929485

[5] Phan TG, Croucher PI. The dormant cancer cell life cycle. Nat Rev Cancer. 2020;20:398–411. 32488200 10.1038/s41568-020-0263-0

[6] Correia AL, Guimarães JC, Auf der Maur P, De Silva D, Trefny MP, Okamoto R, et al. Hepatic stellate cells suppress NK cell-sustained breast cancer dormancy. Nature. 2021;594:566–71. 34079127 10.1038/s41586-021-03614-z

[7] Cackowski FC, Heath EI. Prostate cancer dormancy and recurrence. Cancer Lett. 2022;524:103–8. 34624433 10.1016/j.canlet.2021.09.037 PMC8694498

[8] Zhang M, Peng R, Wang H, Yang Z, Zhang H, Zhang Y, et al. Nanog mediated by FAO/ACLY signaling induces cellular dormancy in colorectal cancer cells. Cell Death Dis. 2022;13:159. 35177584 10.1038/s41419-022-04606-1 PMC8854412

[9] Khalil BD, Sanchez R, Rahman T, Rodriguez-Tirado C, Moritsch S, Martinez AR, et al. An NR2F1-specific agonist suppresses metastasis by inducing cancer cell dormancy. J Exp Med. 2021;219:e20210836. 34812843 10.1084/jem.20210836 PMC8614154

[10] Hu J, Sánchez-Rivera FJ, Wang Z, Johnson GN, Ho Y-J, Ganesh K, et al. STING inhibits the reactivation of dormant metastasis in lung adenocarcinoma. Nature. 2023;616:806–13. 36991128 10.1038/s41586-023-05880-5 PMC10569211

[11] Dai J, Cimino PJ, Gouin KH, Grzelak CA, Barrett A, Lim AR, et al. Astrocytic laminin-211 drives disseminated breast tumor cell dormancy in brain. Nat Cancer. 2022;3:25–42. 35121993 10.1038/s43018-021-00297-3 PMC9469899

[12] Tufail M, Jiang C-H, Li N. Tumor dormancy and relapse: understanding the molecular mechanisms of cancer recurrence. Mil Med Res. 2025;12:7. 39934876 10.1186/s40779-025-00595-2 PMC11812268

[13] Bakhshandeh S, Heras U, Taïeb HM, Varadarajan AR, Lissek SM, Hücker SM, et al. Dormancy-inducing 3D engineered matrix uncovers mechanosensitive and drug-protective FHL2-p21 signaling axis. Sci Adv. 2024;10:eadr3997. 39504377 10.1126/sciadv.adr3997 PMC11540038

[14] Ohta Y, Fujii M, Takahashi S, Takano A, Nanki K, Matano M, et al. Cell-matrix interface regulates dormancy in human colon cancer stem cells. Nature. 2022;608:784–94. 35798028 10.1038/s41586-022-05043-y

[15] Singh DK, Carcamo S, Farias EF, Hasson D, Zheng W, Sun D, et al. 5-Azacytidine- and retinoic-acid-induced reprogramming of DCCs into dormancy suppresses metastasis via restored TGF-β-SMAD4 signaling. Cell Rep. 2023;42:112560. 37267946 10.1016/j.celrep.2023.112560 PMC10592471

[16] Calo E, Flynn RA, Martin L, Spitale RC, Chang HY, Wysocka J. RNA helicase DDX21 coordinates transcription and ribosomal RNA processing. Nature. 2015;518:249–53. 25470060 10.1038/nature13923 PMC4827702

[17] Kim DS, Camacho CV, Nagari A, Malladi VS, Challa S, Kraus WL. Activation of PARP-1 by snoRNAs controls ribosome biogenesis and cell growth via the RNA Helicase DDX21. Mol Cell. 2019;75:1270–85 e14. 31351877 10.1016/j.molcel.2019.06.020 PMC6754283

[18] Lu P, Yu Z, Wang K, Zhai Y, Chen B, Liu M, et al. DDX21 interacts with WDR5 to promote colorectal cancer cell proliferation by activating CDK1 expression. J Cancer. 2022;13:1530–9. 35371306 10.7150/jca.69216 PMC8965128

[19] Bekker-Jensen S, Rendtlew Danielsen J, Fugger K, Gromova I, Nerstedt A, Lukas C, et al. HERC2 coordinates ubiquitin-dependent assembly of DNA repair factors on damaged chromosomes. Nat Cell Biol. 2010;12:80–6. 20023648 10.1038/ncb2008

[20] Mathieu NA, Levin RH, Spratt DE. Exploring the roles of HERC2 and the NEDD4L HECT E3 ubiquitin ligase subfamily in p53 signaling and the DNA damage response. Front Oncol. 2021;11:659049. 33869064 10.3389/fonc.2021.659049 PMC8044464

[21] Pan H, Song Y, Zhang H, Bai Y, Konishi T, Kobayashi A, et al. Radiation engenders converse migration and invasion in colorectal cancer cells through opposite modulation of ANXA2/AKT/GSK3beta pathway. Am J Cancer Res. 2021;11:61–78. 33520360 PMC7840724

[22] Pan Y, Ye S, Yuan D, Zhang J, Bai Y, Shao C. Hydrogen sulfide (H2S)/cystathionine gamma-lyase (CSE) pathway contributes to the proliferation of hepatoma cells. Mutat Res. 2014;763-764:10–8. 24657251 10.1016/j.mrfmmm.2014.03.002

[23] Li H, Rokavec M, Jiang L, Horst D, Hermeking H. Antagonistic effects of p53 and HIF1A on microRNA-34a regulation of PPP1R11 and STAT3 and hypoxia-induced epithelial to mesenchymal transition in colorectal cancer cells. Gastroenterology. 2017;153:505–20. 28435028 10.1053/j.gastro.2017.04.017

[24] Gotz S, García-Gómez JM, Terol J, Williams TD, Nagaraj SH, Nueda MJ, et al. High-throughput functional annotation and data mining with the Blast2GO suite. Nucleic Acids Res. 2008;36:3420–35. 18445632 10.1093/nar/gkn176 PMC2425479

[25] Bardou P, Mariette J, Escudie F, Djemiel C, Klopp C. jvenn: an interactive Venn diagram viewer. BMC Bioinforma. 2014;15:293. 10.1186/1471-2105-15-293 PMC4261873 25176396

[26] Pierce BG, Wiehe K, Hwang H, Kim B-H, Vreven T, Weng Z. ZDOCK server: interactive docking prediction of protein-protein complexes and symmetric multimers. Bioinformatics. 2014;30:1771–3. 24532726 10.1093/bioinformatics/btu097 PMC4058926

[27] Francescangeli F, Contavalli P, De Angelis ML, Careccia S, Signore M, Haas TL, et al. A pre-existing population of ZEB2+ quiescent cells with stemness and mesenchymal features dictate chemoresistance in colorectal cancer. J Exp Clin Cancer Res: CR. 2020;39:2. 31910865 10.1186/s13046-019-1505-4 PMC6947904

[28] Hauge S, Eek Mariampillai A, Rødland GE, Bay LTE, Landsverk HB, Syljuåsen RG. Expanding roles of cell cycle checkpoint inhibitors in radiation oncology. Int J Radiat Biol. 2021;99:941–50. 33877959 10.1080/09553002.2021.1913529

[29] Oki T, Nishimura K, Kitaura J, Togami K, Maehara A, Izawa K, et al. A novel cell-cycle-indicator, mVenus-p27K-, identifies quiescent cells and visualizes G0-G1 transition. Sci Rep. 2014;4:4012. 24500246 10.1038/srep04012 PMC3915272

[30] Goddard ET, Linde MH, Srivastava S, Klug G, Shabaneh TB, Iannone S, et al. Immune evasion of dormant disseminated tumor cells is due to their scarcity and can be overcome by T cell immunotherapies. Cancer Cell. 2024;42:119–34. e12. 38194912 10.1016/j.ccell.2023.12.011 PMC10864018

[31] Sosa MS, Parikh F, Maia AG, Estrada Y, Bosch A, Bragado P, et al. NR2F1 controls tumour cell dormancy via SOX9- and RARβ-driven quiescence programmes. Nat Commun. 2015;6:6170. 25636082 10.1038/ncomms7170 PMC4313575

[32] Yu-Lee LY, Yu G, Lee YC, Lin SC, Pan J, Pan T, et al. Osteoblast-secreted factors mediate dormancy of metastatic prostate cancer in the bone via activation of the TGFbetaRIII-p38MAPK-pS249/T252RB pathway. Cancer Res. 2018;78:2911–24. 29514796 10.1158/0008-5472.CAN-17-1051 PMC5984689

[33] Maik-Rachline G, Lifshits L, Seger R. Nuclear P38: roles in physiological and pathological processes and regulation of nuclear translocation. Int J Mol Sci. 2020;21:6102. 32847129 10.3390/ijms21176102 PMC7504396

[34] Hume S, Grou CP, Lascaux P, D’Angiolella V, Legrand AJ, Ramadan K, et al. The NUCKS1-SKP2-p21/p27 axis controls S phase entry. Nat Commun. 2021;12:6959. 34845229 10.1038/s41467-021-27124-8 PMC8630071

[35] Vargas JNS, Hamasaki M, Kawabata T, Youle RJ, Yoshimori T. The mechanisms and roles of selective autophagy in mammals. Nat Rev Mol Cell Biol. 2023;24:167–85. 36302887 10.1038/s41580-022-00542-2

[36] Gu X, Nardone C, Kamitaki N, Mao A, Elledge SJ, Greenberg ME. The midnolin-proteasome pathway catches proteins for ubiquitination-independent degradation. Science. 2023;381:eadh5021. 37616343 10.1126/science.adh5021 PMC10617673

[37] Xu P, Liu Y, Liu C, Guey B, Li L, Melenec P, et al. The CRL5-SPSB3 ubiquitin ligase targets nuclear cGAS for degradation. Nature. 2024;627:873–9. 38418882 10.1038/s41586-024-07112-w PMC10972748

[38] Anandhan A, Dodson M, Shakya A, Chen J, Liu P, Wei Y, et al. NRF2 controls iron homeostasis and ferroptosis through HERC2 and VAMP8. Sci Adv. 2023;9:eade9585. 36724221 10.1126/sciadv.ade9585 PMC9891695

[39] Swatek KN, Komander D. Ubiquitin modifications. Cell Res. 2016;26:399–422. 27012465 10.1038/cr.2016.39 PMC4822133

[40] Recasens A, Munoz L. Targeting cancer cell dormancy. Trends Pharmacol Sci. 2019;40:128–41. 30612715 10.1016/j.tips.2018.12.004

[41] Agudo J, Aguirre-Ghiso JA, Bhatia M, Chodosh LA, Correia AL, Klein CA. Targeting cancer cell dormancy. Nat Rev Cancer. 2024;24:97–104. 38062251 10.1038/s41568-023-00642-x PMC11038906

[42] Heinz MC, Peters NA, Oost KC, Lindeboom RGH, van Voorthuijsen L, Fumagalli A, et al. Liver colonization by colorectal cancer metastases requires YAP-controlled plasticity at the micrometastatic stage. Cancer Res. 2022;82:1953–68. 35570706 10.1158/0008-5472.CAN-21-0933 PMC9381095

[43] Hosseini H, Obradović MMS, Hoffmann M, Harper KL, Sosa MS, Werner-Klein M, et al. Early dissemination of seeds metastasis in breast cancer. Nature. 2016;540:552–8. 27974799 10.1038/nature20785 PMC5390864

[44] Puig I, Tenbaum SP, Chicote I, Arqués O, Martínez-Quintanilla J, Cuesta-Borrás E, et al. TET2 controls chemoresistant slow-cycling cancer cell survival and tumor recurrence. J Clin Investig. 2018;128:3887–905. 29944140 10.1172/JCI96393 PMC6118637

[45] Hoang-Minh LB, Siebzehnrubl FA, Yang C, Suzuki-Hatano S, Dajac K, Loche T, et al. Infiltrative and drug-resistant slow-cycling cells support metabolic heterogeneity in glioblastoma. EMBO J. 2018;37:e98772. 30322894 10.15252/embj.201798772 PMC6276884

[46] Bora P, Gahurova L, Hauserova A, Stiborova M, Collier R, Potesil D, et al. DDX21 is a p38-MAPK-sensitive nucleolar protein necessary for mouse preimplantation embryo development and cell-fate specification. Open Biol. 2021;11:210092. 34255976 10.1098/rsob.210092 PMC8277471

[47] Putra V, Hulme AJ, Tee AE, Sun JQJ, Atmadibrata B, Ho N, et al. The RNA-helicase DDX21 upregulates CEP55 expression and promotes neuroblastoma. Mol Oncol. 2021;15:1162–79. 33497018 10.1002/1878-0261.12906 PMC8024731

[48] Tang W, Yang Y, Fu Z, Xu W, Ou W, Liu F, et al. The RNA helicase DDX21 activates YAP to promote tumorigenesis and is transcriptionally upregulated by β-catenin in colorectal cancer. Oncogene. 2024;43:3227–39. 39285230 10.1038/s41388-024-03160-8 PMC11518987

[49] Wang K, Li B, Fan P, Ren X, Jiang H. Downregulation of DEAD-box helicase 21 (DDX21) inhibits proliferation, cell cycle, and tumor growth in colorectal cancer via targeting cell division cycle 5-like (CDC5L). Bioengineered. 2021;12:12647–58. 34903139 10.1080/21655979.2021.2011636 PMC8810101

[50] Koltowska K, Okuda KS, Gloger M, Rondon-Galeano M, Mason E, Xuan J, et al. The RNA helicase Ddx21 controls Vegfc-driven developmental lymphangiogenesis by balancing endothelial cell ribosome biogenesis and p53 function. Nat Cell Biol. 2021;23:1136–47. 34750583 10.1038/s41556-021-00784-w

[51] Tanaka A, Wang JY, Shia J, Zhou Y, Ogawa M, Hendrickson RC, et al. DEAD-box RNA helicase protein DDX21 as a prognosis marker for early-stage colorectal cancer with microsatellite instability. Sci Rep. 2020;10:22085. 33328538 10.1038/s41598-020-79049-9 PMC7745018

[52] Zhang H, Zhang Y, Chen C, Zhu X, Zhang C, Xia Y, et al. A double-negative feedback loop between DEAD-box protein DDX21 and Snail regulates epithelial-mesenchymal transition and metastasis in breast cancer. Cancer Lett. 2018;437:67–78. 30165191 10.1016/j.canlet.2018.08.021

[53] Borgen E, Rypdal MC, Sosa MS, Renolen A, Schlichting E, Lønning PE, et al. NR2F1 stratifies dormant disseminated tumor cells in breast cancer patients. Breast Cancer Res. 2018;20:120. 30322396 10.1186/s13058-018-1049-0 PMC6190561

[54] Lin X, Kang K, Chen P, Zeng Z, Li G, Xiong W, et al. Regulatory mechanisms of PD-1/PD-L1 in cancers. Mol Cancer. 2024;23:108. 38762484 10.1186/s12943-024-02023-w PMC11102195

[55] Weng J, Li S, Zhu Z, Liu Q, Zhang R, Yang Y, et al. Exploring immunotherapy in colorectal cancer. J Hematol Oncol. 2022;15:95. 35842707 10.1186/s13045-022-01294-4 PMC9288068

[56] Richbourg NR, Irakoze N, Kim H, Peyton SR. Outlook and opportunities for engineered environments of breast cancer dormancy. Sci Adv. 2024;10:eadl0165. 38457510 10.1126/sciadv.adl0165 PMC10923521

[57] Wei J-R, Zhang B, Zhang Y, Chen W-M, Zhang X-P, Zeng T-T, et al. QSOX1 facilitates dormant esophageal cancer stem cells to evade immune elimination via PD-L1 upregulation and CD8 T cell exclusion. Proc Natl Acad Sci USA. 2024;121:e2407506121. 39432781 10.1073/pnas.2407506121 PMC11536095

[58] Sosa MS, Avivar-Valderas A, Bragado P, Wen H-C, Aguirre-Ghiso JA. ERK1/2 and p38α/β signaling in tumor cell quiescence: opportunities to control dormant residual disease. Clin Cancer Res. 2011;17:5850–7. 21673068 10.1158/1078-0432.CCR-10-2574 PMC3226348

[59] Schewe DM, Aguirre-Ghiso JA. ATF6alpha-Rheb-mTOR signaling promotes survival of dormant tumor cells in vivo. Proc Natl Acad Sci USA. 2008;105:10519–24. 18650380 10.1073/pnas.0800939105 PMC2492459

[60] Correa RJM, Peart T, Valdes YR, DiMattia GE, Shepherd TG. Modulation of AKT activity is associated with reversible dormancy in ascites-derived epithelial ovarian cancer spheroids. Carcinogenesis. 2012;33:49–58. 22045027 10.1093/carcin/bgr241

[61] Guereno M, Delgado Pastore M, Lugones AC, Cercato M, Todaro L, Urtreger A, et al. Glypican-3 (GPC3) inhibits metastasis development promoting dormancy in breast cancer cells by p38 MAPK pathway activation. Eur J Cell Biol. 2020;99:151096. 32800275 10.1016/j.ejcb.2020.151096

[62] Sato K, Yoshino H, Sato Y, Sasaki F, Munakata N, Tsuruga E. Impact of the ATM/Chk2 pathway and cell cycle phase on radiation-induced senescence in A549 human lung cancer cells. Biomed Rep. 2025;23:169. 40927689 10.3892/br.2025.2047 PMC12415813

[63] Menegakis A, Klompmaker R, Vennin C, Arbusà A, Damen M, van den Broek B, et al. Resistance of hypoxic cells to ionizing radiation is mediated in part via hypoxia-induced quiescence. Cells. 2021;10:610. 33801903 10.3390/cells10030610 PMC7998378

[64] Pereira P, Panier J, Nater M, Herbst M, Calvanese AL, Köksal H, et al. Inflammatory cytokines mediate the induction of and awakening from metastatic dormancy. Cell Rep. 2025;44:115388. 40023846 10.1016/j.celrep.2025.115388

[65] Zhang J, Zhang J, Han L, Wu S, Li J, Eaton EN, et al. Inflammation awakens dormant cancer cells by modulating the epithelial-mesenchymal phenotypic state. Proc Natl Acad Sci USA. 2025;122:e2515009122. 40901881 10.1073/pnas.2515009122 PMC12435312

[66] Bakir M, Dabaliz A, Dawalibi A, Mohammad KS. Microenvironmental and molecular pathways driving dormancy escape in bone metastases. Int J Mol Sci. 2025;26:11893. 41465319 10.3390/ijms262411893 PMC12732430

[67] Ridwan SM, Emlein R, Mesbahi A, Annabi A, Hainfeld JF, Smilowitz HM. Radiation-induced dormancy of intracerebral melanoma: endotoxin inflammation leads to both shortened tumor dormancy and long-term survival with localized senescence. Cancer Immunol Immunotherapy. 2023;72:3851–9. 10.1007/s00262-023-03481-9 PMC10992577 37612405

[68] Maranon DG, Sharma N, Huang Y, Selemenakis P, Wang M, Altina N, et al. NUCKS1 promotes RAD54 activity in homologous recombination DNA repair. J Cell Biol. 2020;219:e201911049. 32876692 10.1083/jcb.201911049 PMC7659731

[69] Zheng S, Ji R, He H, Li N, Han C, Han J, et al. NUCKS1, a LINC00629-upregulated gene, facilitated osteosarcoma progression and metastasis by elevating asparagine synthesis. Cell Death Dis. 2023;14:489. 37528150 10.1038/s41419-023-06010-9 PMC10393983

[70] Gao H, Wei H, Yang Y, Li H, Liang J, Ye J, et al. Phase separation of DDX21 promotes colorectal cancer metastasis via MCM5-dependent EMT pathway. Oncogene. 2023;42:1704–15. 37029300 10.1038/s41388-023-02687-6 PMC10202810

[71] Zhang N, Wang B, Ma C, Zeng J, Wang T, Han L, et al. LINC00240 in the 6p22.1 risk locus promotes gastric cancer progression through USP10-mediated DDX21 stabilization. J Exp Clin Cancer Res. 2023;42:89. 37072811 10.1186/s13046-023-02654-9 PMC10111703

[72] Zhang G, Yang R, Wang B, Yan Q, Zhao P, Zhang J, et al. TRIP13 regulates progression of gastric cancer through stabilising the expression of DDX21. Cell Death Dis. 2024;15:622. 39187490 10.1038/s41419-024-07012-x PMC11347623

[73] Liu Y, Xu Q, Deng F, Zheng Z, Luo J, Wang P, et al. HERC2 promotes inflammation-driven cancer stemness and immune evasion in hepatocellular carcinoma by activating STAT3 pathway. J Exp Clin Cancer Res. 2023;42:38. 36721234 10.1186/s13046-023-02609-0 PMC9890722

[74] Liu Y, Xu Q, Liu Y, Cao S, Luo J, Zheng Z, et al. Hepatocyte-targeted lipid nanoparticle delivery of HERC2 plasmid controls drug-induced hepatotoxicity by limiting β-catenin-regulated CYP2E1 expression. Adv Sci. 2024;11:e2401633. 10.1002/advs.202401633 PMC11633468 39440550

[75] Song A, Liu B, Li W, Chen B, Gui P, Zhang H, et al. Competitive binding between DDX21 and SIRT7 enhances NAT10-mediated ac4C modification to promote colorectal cancer metastasis and angiogenesis- DDX21 promotes colorectal cancer metastasis. Cell Death Dis. 2025;16:353. 40301349 10.1038/s41419-025-07656-3 PMC12041575

[76] Feng J, Xi Z, Jiang X, Li Y, Nik Nabil WN, Liu M, et al. Saikosaponin A enhances Docetaxel efficacy by selectively inducing death of dormant prostate cancer cells through excessive autophagy. Cancer Lett. 2022;554:216011. 36442771 10.1016/j.canlet.2022.216011

[77] Hnit SST, Bi L, Xie C, Xu L, Zhong Y, Yang M, et al. Inhibition of dormant lung cancer cell reactivation by Punica granatum peel and Dioscorea nipponica: involving MYC, SKP2 and p27. Drug Des Devel Ther. 2025;19:3997–4010. 40391177 10.2147/DDDT.S494168 PMC12087594

[78] Roper J, Tammela T, Cetinbas NM, Akkad A, Roghanian A, Rickelt S, et al. In vivo genome editing and organoid transplantation models of colorectal cancer and metastasis. Nat Biotechnol. 2017;35:569–76. 28459449 10.1038/nbt.3836 PMC5462879

[79] Galligan JT, Martinez-Noël G, Arndt V, Hayes S, Chittenden TW, Harper JW, et al. Proteomic analysis and identification of cellular interactors of the giant ubiquitin ligase HERC2. J Proteome Res. 2014;14:953–66. 25476789 10.1021/pr501005v PMC4324439

