# Supplementary material for: Ubiquitination of DDX21 by HERC2 induces a dormancy-like phenotype via the NUCKS1-p21/p27 axis to promote radio-resistance in colorectal cancer cells
Source: Cell Death Dis. 2026 May 3;17(1):588. doi: 10.1038/s41419-026-08811-0 (PMC13284300; doi:10.1038/s41419-026-08811-0)
Supplement: Supplementary file 1 — Supplementary Figures and Tables [file 41419_2026_8811_MOESM1_ESM.pdf]

**Ubiquitination of DDX21 by HERC2 induces a dormancy-like phenotype via the NUCKS1-p21/p27 axis to promote radioresistance in colorectal cancer cells**

# Supplementary figures and tables

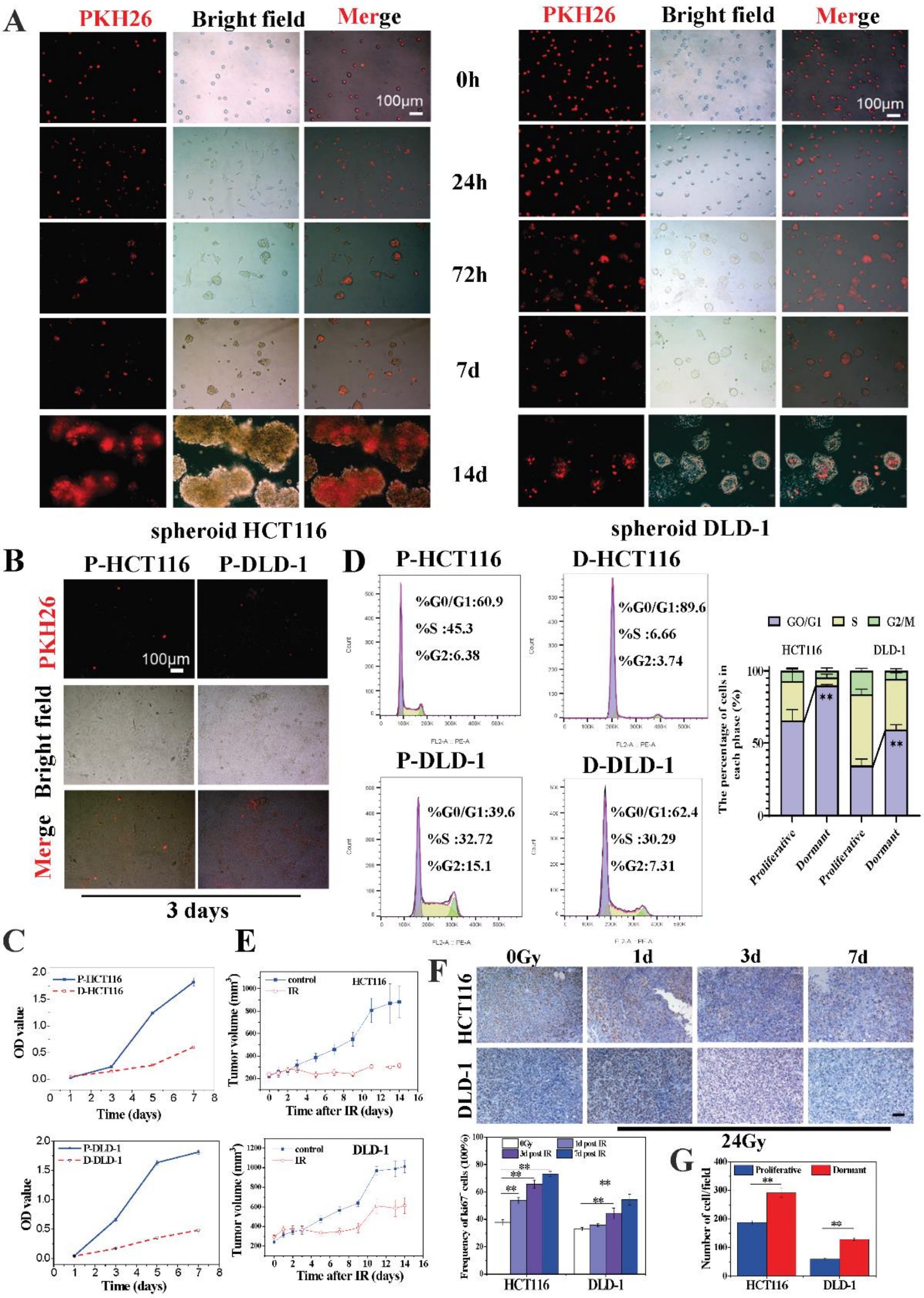


**Figure S1. Label-retaining cells within colon cancer spheroids exhibit a dormancy-like state. (A)** Representative images of PKH26-stained HCT116 and DLD-1 spheroids over a 14-day period. **(B)** Representative images of PKH26-stained HCT116 and DLD-1 cells cultured in standard medium at day 3. **(C)** The growth curves of proliferative and dormancy-like CRC cells. **(D)** Cell cycle analysis of proliferative and dormancy-like CRC cells. *P* values indicate the statistical significance relative to the percentages of G0/G1 phase. **(E)** The growth curves of xenograft tumor of HCT116 (above) and DLD-1 (below) ((n=6 or 8/group). **(F)** IHC quantification of Ki67-negative (dormancy-like) CRC cells in tumor tissues at days 1, day3, and day7 post-irradiation. Scale bar, 100 μm. **(G)** Statistical column plots of the migration assay of the dormancy-like HCT116 and DLD-1 cells compared with their proliferative counterparts. D/P indicates the cells at dormancy-like or proliferative state**.** The data are presented as means ± SD, ** *P*<0.01.

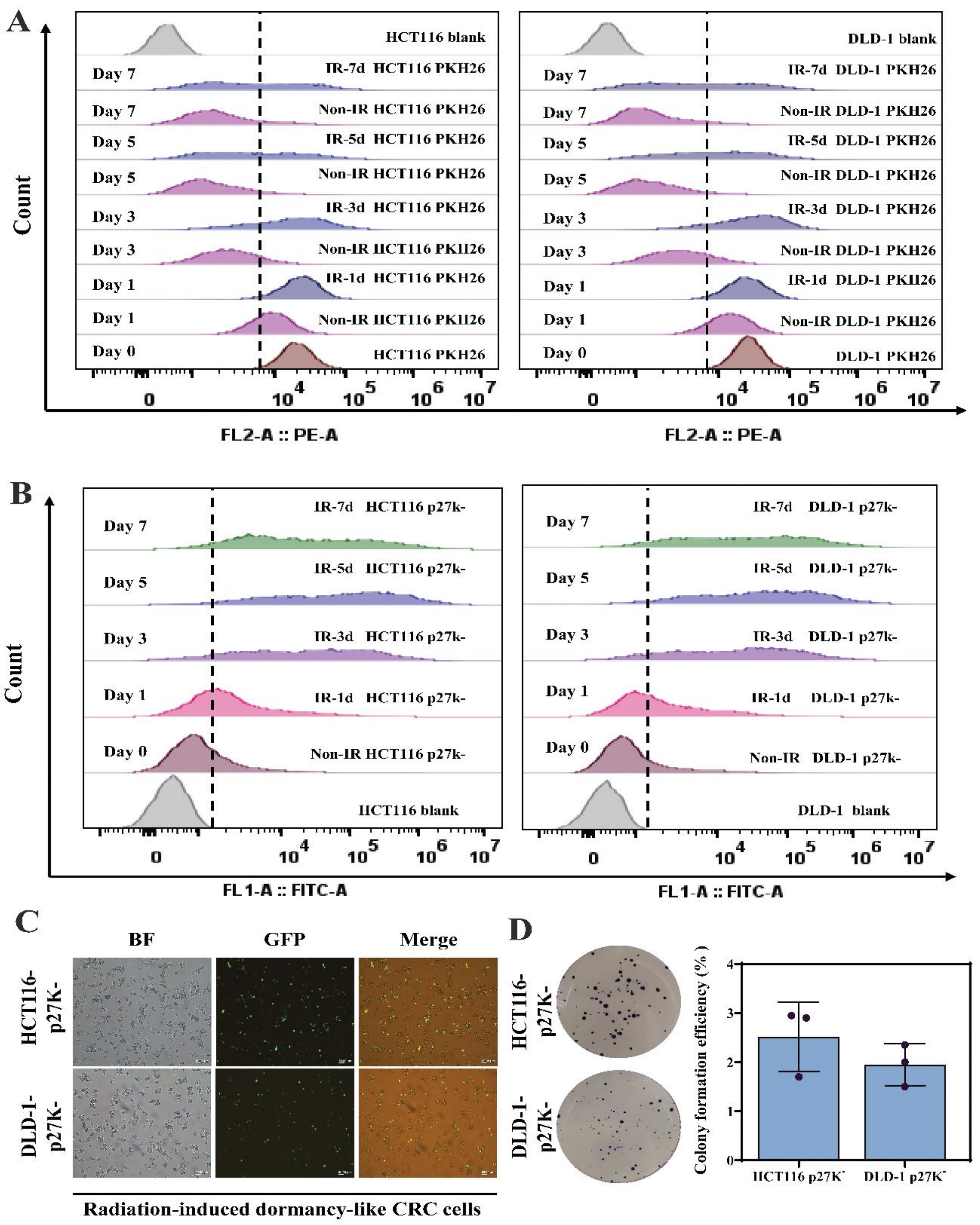


**Figure S2. Radiation induces CRC cells into a dormancy-like state. (A)** PKH26 dye retention in HCT116 and DLD-1 cells on days 0, 1, 3, 5, and 7 post-irradiation following membrane labeling with PKH26. **(B)** EGFP fluorescence intensity in HCT116 and DLD-1 cells stably expressing the p27K$^{-}$ reporter on days 0, 1, 3, 5, and 7 post-irradiation. **(C)** Fluorescence images of sorted radiation-induced dormancy-like populations from HCT116 and DLD-1 cells on day 5 post-irradiation, captured 24 h after sorting. Scale bar, 50 µm. **(D)** Representative images (left) and quantification (right) of colony formation by sorted radiation-induced dormancy-like CRC cells. Cells were seeded at 1000 cells per well and cultured for 10 days. Colonies were fixed, stained with crystal violet, and counted. Colony formation efficiency was calculated as (number of colonies / number of seeded cells) × 100%. The data are presented as means ± SD.

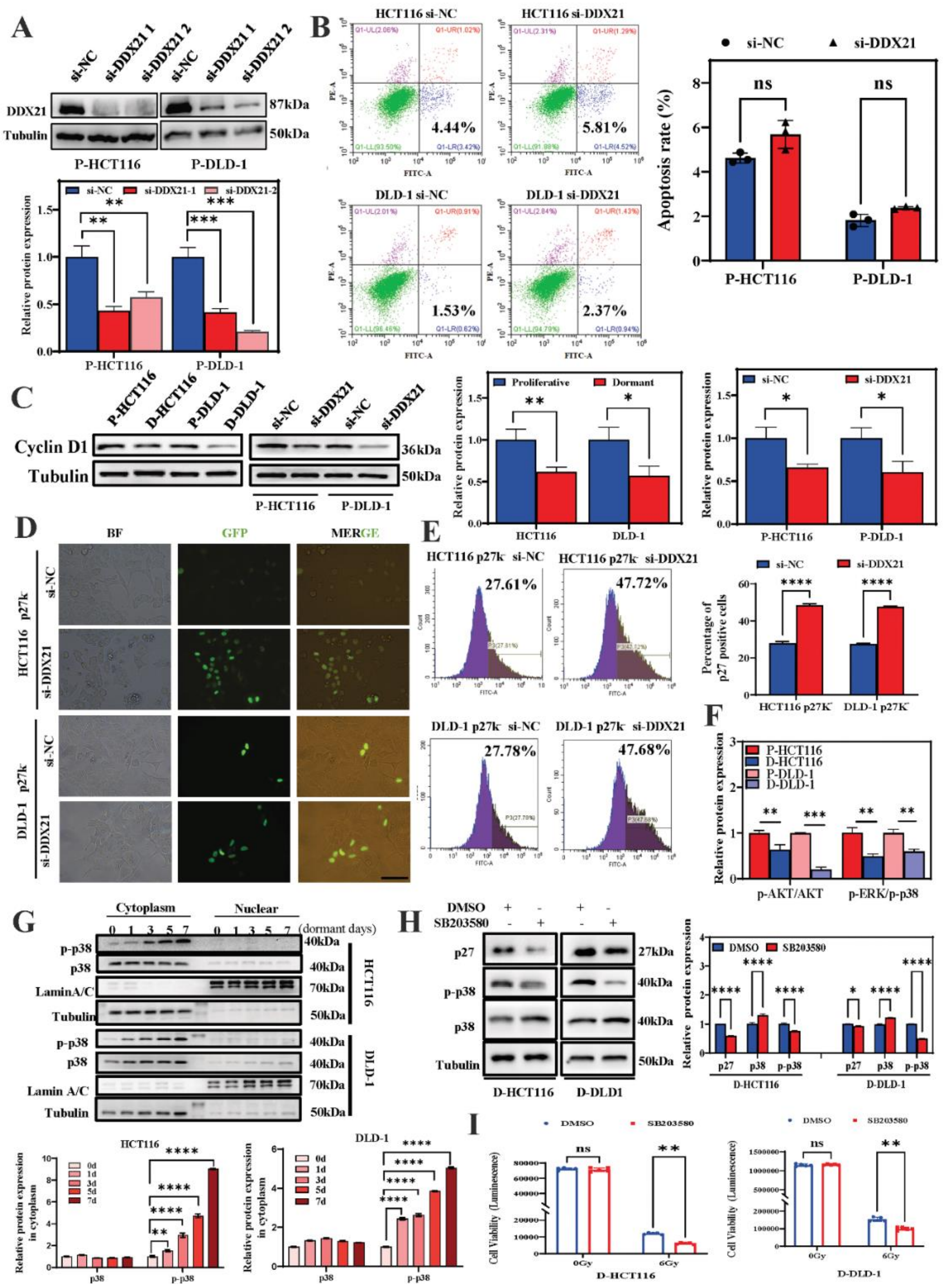


**Figure S3. Down-regulation of DDX21 induces CRC cells into dormancy-like state. (A)** Western blot assay showing the interference efficiency of siRNA-mediated DDX21 knockdown in the proliferative HCT116 and DLD-1 cells. **(B)** Apoptosis rates of proliferative HCT116 and DLD-1 cells after DDX21 knockdown. **(C)** Western blot assay of Cyclin D1 in proliferative and dormancy-like CRC cells with or without si-DDX21 transfection. **(D)** Representative images of EGFP-p27K$^-$ fluorescence (a dormancy marker) in proliferative CRC cells after DDX21 knockdown. Scale bar, 100 μm. **(E)** Flow cytometry analysis (left) and quantification (right) of EGFP-p27K$^-$ positive dormancy-like cells following DDX21 knockdown in CRC cells. **(F)** Western blot assay of p-AKT, AKT, p-ERK, ERK, p-p38, and p38 proteins in the proliferative versus dormancy-like CRC cells. The p-ERK/p-p38 ratio was calculated by first normalizing p-ERK to total ERK and p-p38 to total p38, then dividing the normalized p-ERK value by the normalized p-p38 value. Quantitative results are presented as bar graphs. **(G)** Western blot assay of p38 and p-p38 in the cytoplasm and nuclear lysates of CRC cells at different time points during dormancy-like induction process. **(H)** Western blot assay of p27, p38, and p-p38 in dormancy-like CRC cells treated with or without 10 μM SB203580. **(I)** Cell viability of dormancy-like CRC cells pretreated with or without 10 μM SB203580 for 2 hours followed by 6 Gy irradiation. D/P indicates the cells at dormancy-like or proliferative state. The data are presented as means ± SD, *$P < 0.05$, **$P < 0.01$, *** $P < 0.001$, **** $P < 0.0001$.

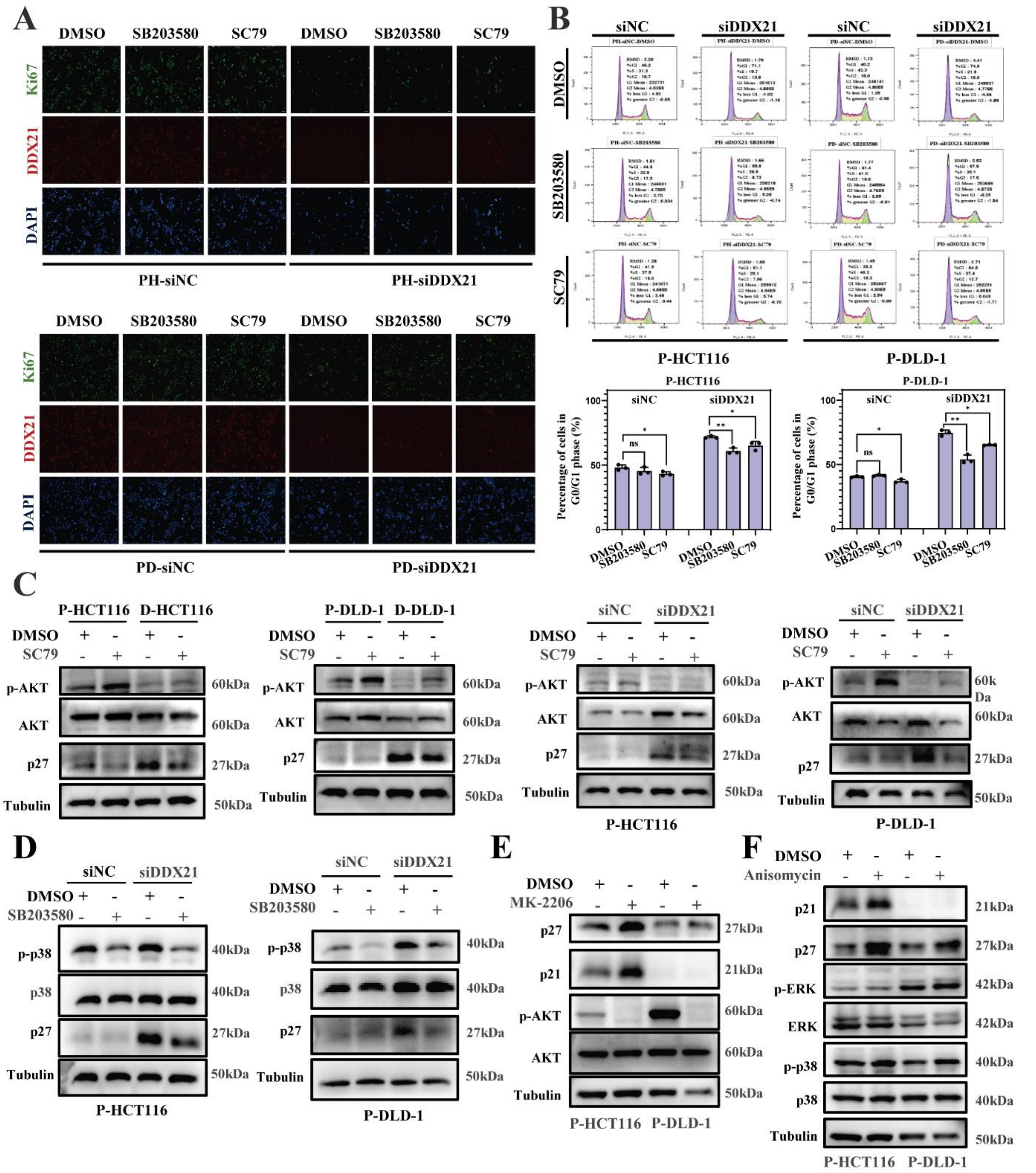


**Figure S4. DDX21 knockdown induces a dormancy-like state in CRC cells via the p38MAPK/AKT signaling pathway. (A)** Representative immunofluorescence images of Ki67 (green), DDX21 (red), and DAPI (blue) in proliferative CRC cells following DDX21 knockdown, with or without treatment with the p-p38 inhibitor SB203580 or the p-AKT activator SC79. Cells treated with 0.1% DMSO served as a control. Scale bar, 50 μm. **(B)** Cell cycle distribution of proliferative CRC cells under the same conditions as in (A). *P* values indicate statistical significance compared to the G0/G1 phase percentage of the control group. **(C)** Western blot assay of p-AKT, total AKT, and p27 in proliferative, dormancy-like, and DDX21-KD CRC cells, with or without SC79 treatment. **(D)** Western blot assay of p-p38, total p38, and p27 in DDX21-KD CRC cells, with or without SB203580 treatment. **(E)** Western blot assay of p-AKT, total AKT, p21, and p27 in proliferative CRC cells treated with the p-AKT inhibitor MK-2206. **(F)** Western blot assay of p-p38, total p38, p-ERK, total ERK, p21, and p27 in proliferative CRC cells with the p-p38 activator Anisomycin. D/P indicates the cells at dormancy-like or proliferative state. The data are presented as means ± SD, *$P < 0.05$, **$P < 0.01$, *** $P < 0.001$.

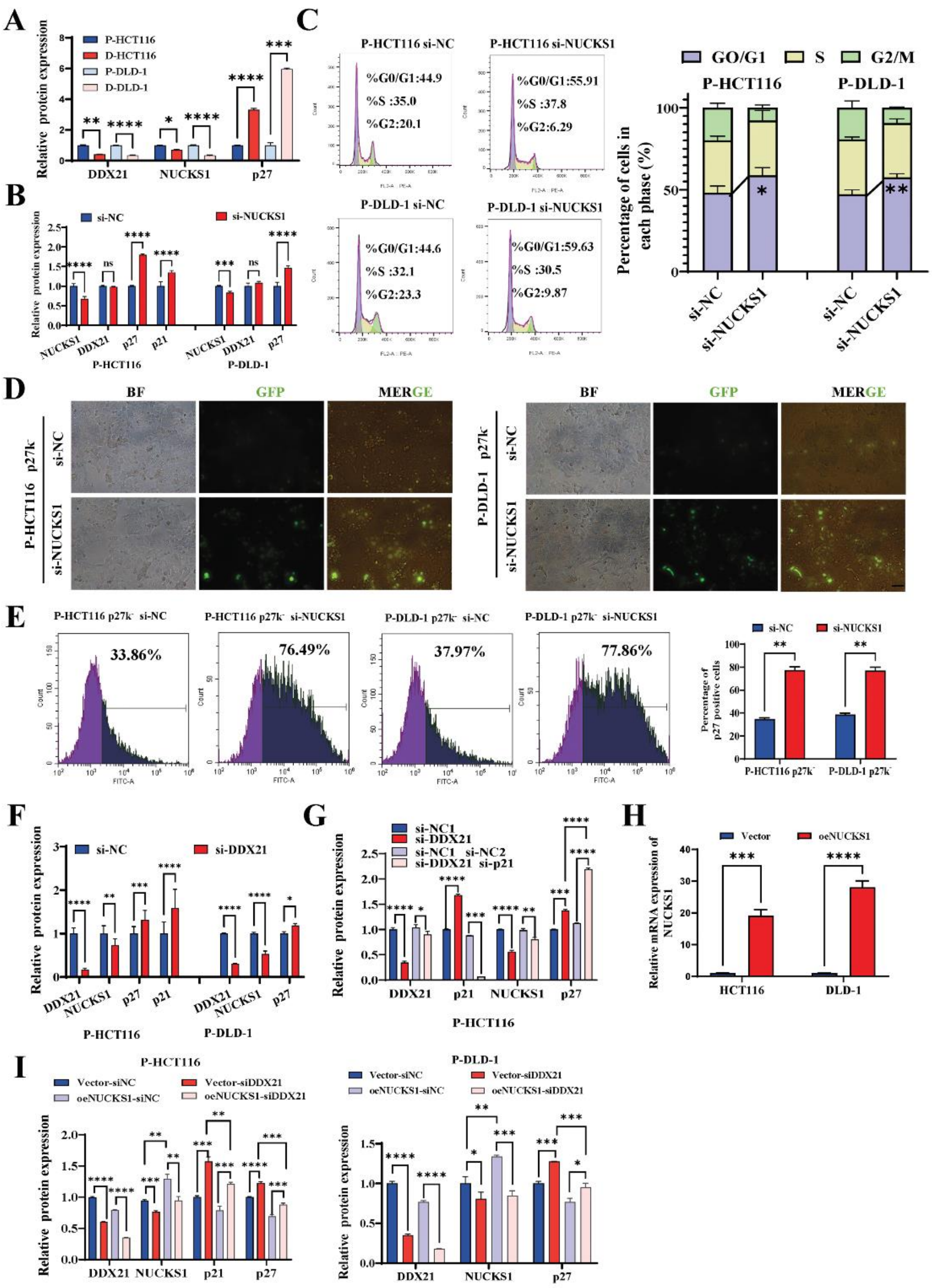


**Figure S5. Low expression of DDX21 induces CRC cells into dormancy through DDX21-NUCKS1-p27/p21 axis. (A)** Relative protein expression of DDX21, NUCKS1 and p27 in proliferative and dormancy-like CRC cells. **(B)** Relative protein expression of DDX21, NUCKS1, p21, and p27 in proliferative CRC cells following NUCKS1-KD. **(C)** Cell cycle analysis of the proliferative CRC cells with NUCKS1 siRNA transfection. *P* values indicate the statistical significance relative to the percentages of G0/G1 phase. **(D)** Representative images of EGFP-p27K$^-$ fluorescence in proliferative CRC cells after NUCKS1 knockdown. Scale bar, 50 μm. **(E)** Flow cytometry analysis (left) and quantification (right) of EGFP-p27K$^-$positive dormancy-like cells following NUCKS1 knockdown in CRC cells. **(F)** Relative protein expression of DDX21, NUCKS1, p27, and p21 in proliferative CRC cells following DDX21 knockdown. **(G)** Relative protein expression of DDX21, NUCKS1, p21, and p27 in proliferative HCT116 cells after combined treatment of p21 knockdown and DDX21 knockdown. **(H)** Relative mRNA expression of *NUCKS1* in proliferative HCT116 and DLD-1 cells transfected with NUCKS1 overexpression plasmid or empty vector. Data normalized to ACTB and presented as fold change relative to control (Vector). **(I)** Relative protein expression of DDX21, NUCKS1, p21, and p27 in proliferative CRC cells following DDX21-KD with or without transfected with NUCKS1 overexpression plasmid or empty vector. D/P indicates the cells at dormancy-like or proliferative state. The data are presented as means ± SD, $*P < 0.05$, $**P < 0.01$, $***P < 0.001$, $****P < 0.0001$.

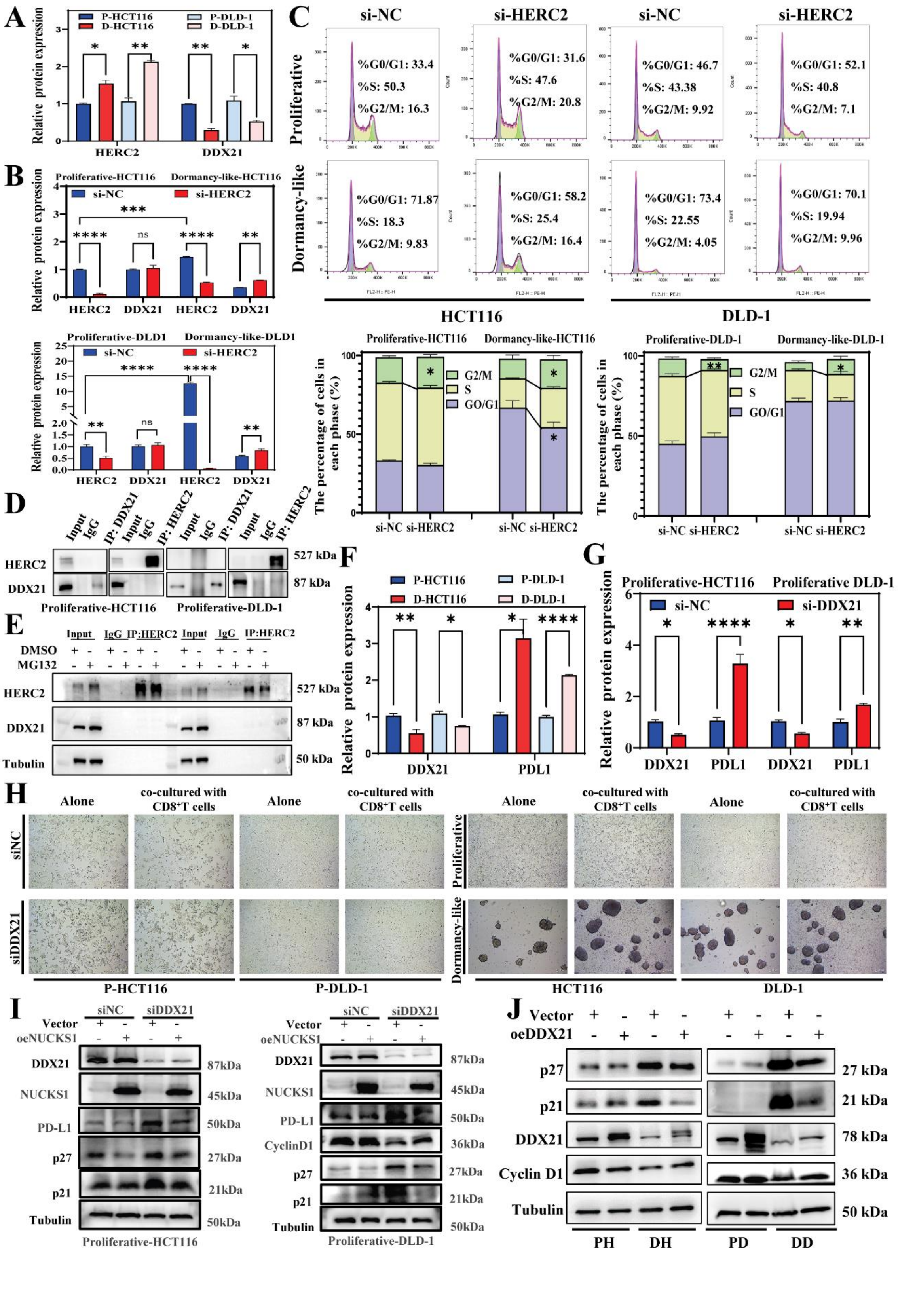
A
B
C
D
E
F
G
H
I
J
HERC2
DDX21
si-NC
si-HERC2
si-DDX21
Proliferative
Dormancy-like
HCT116
DLD-1
Proliferative-HCT116
Proliferative-DLD-1
Relative protein expression
The percentage of cells in each phase (%)
co-cultured with CD8+T cells
Alone
PD-L1
p27
p21
Tubulin
Cyclin D1
NUCKS1

**Figure S6. HERC2-mediated degradation of DDX21 promotes immune evasion and radio-resistance in dormancy-like CRC cells.** (A) Relative protein expression of HERC2 and DDX21 in the proliferative and dormancy-like CRC cells. (B) Relative protein expression of HERC2 and DDX21 in the proliferative and dormancy-like CRC cells following HERC2-KD. (C) Cell cycle analysis of proliferative and dormancy-like CRC cells following HERC2-KD. (D) Co-immunoprecipitation and Western blot assay of HERC2 and DDX21 proteins in the whole cell lysates of proliferative CRC cells. (E) Co-immunoprecipitation and Western blot assay of HERC2 and DDX21 proteins in the whole cell lysates of proliferative CRC cells with the treatment of MG132. (F) Relative protein expressions of DDX21 and PD-L1 in the proliferative and dormancy-like CRC cells. (G) Relative protein expressions of DDX21 and PD-L1 in the proliferative CRC cells following DDX21-KD. (H) Representative images of proliferative CRC cells with or without DDX21 knockdown (left), and of proliferative versus dormancy-like CRC cells (right), after 48 h of co-culture with activated $CD8^+$ T cells. Scale bar, 50 μm. (I) Western blot assay of DDX21, NUCKS1, PD-L1 and dormant-related proteins in CRC proliferative cells transfected with NUCKS1 overexpression plasmid or empty vector with or without the knockdown of DDX21. (J) Western blot assay of DDX21, Cyclin D1, p21, and p27 proteins in the proliferative and dormancy-like CRC cells transfected with DDX21 overexpression plasmid or empty vector. D/P indicates the cells at dormancy-like or proliferative state. The data are presented as means ± SD, *$P < 0.05$, **$P < 0.01$, **** $P < 0.0001$.

**A**

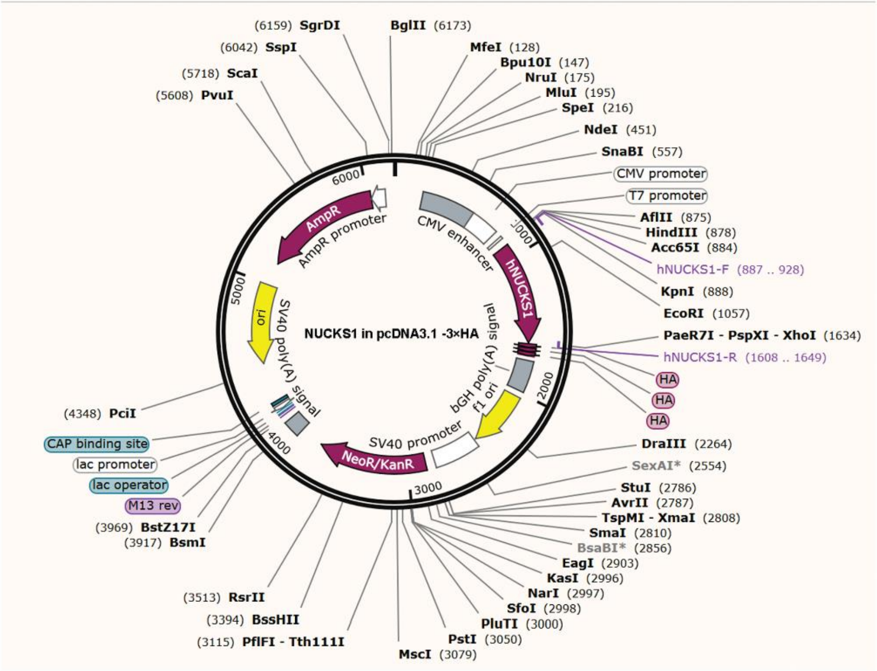


**B**

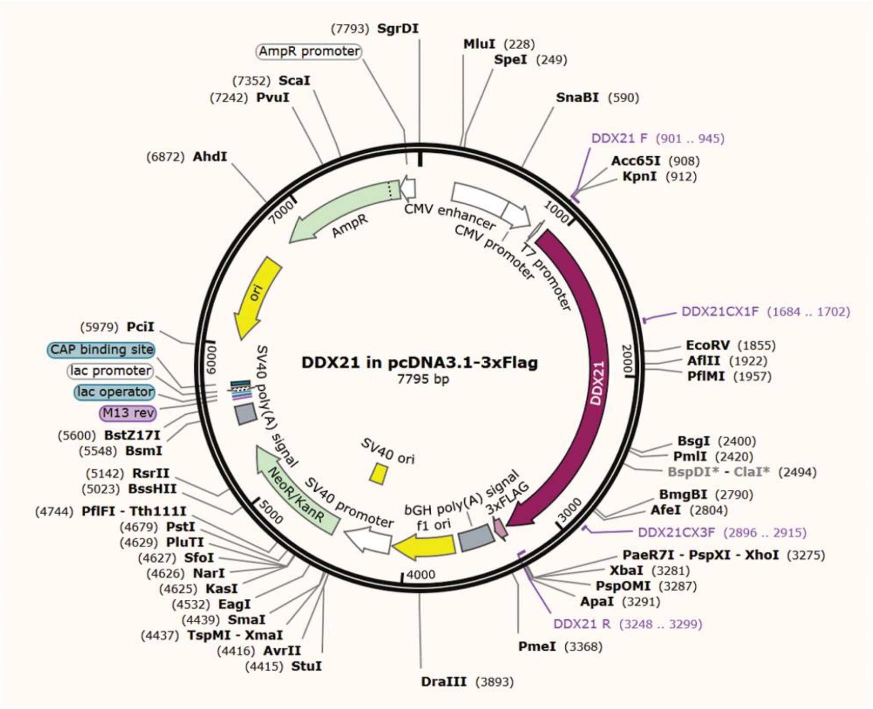


**Figure S7. Plasmid maps of key constructs. (A)** NUCKS1 overexpression vector. Full-length human NUCKS1 cDNA was PCR-amplified and cloned into pcDNA3.1(+) (Invitrogen). All constructs were validated by restriction digestion and sequencing. **(B)** DDX21 overexpression vector. Full-length human DDX21 cDNA was PCR-amplified and cloned into pcDNA3.1(+) (Invitrogen). All constructs were validated by restriction digestion and sequencing.

**Table S1.** Clinical information of 10 different tumor regression grades (TRG) colorectal cancer patients for immunofluorescence and immunohistochemistry assay.

| No. | Pathology number | Sex | Age | T stage | Lymph node metastasis | Distant metastasis | Neoplasm stage | Tumor regression grades (TRG) |
|---|---|---|---|---|---|---|---|---|
| 1 | 814556 | male | 61 | 3 | 2 | 0 | III | 0 |
| 2 | 792269 | male | 65 | 3 | 2 | x | III | 3 |
| 3 | 828805 | male | 64 | 3 | 0 | 0 | II | 0 |
| 4 | 791353 | male | 55 | 3 | x | 0 | II | 1 |
| 5 | 827175 | male | 56 | 4 | 2 | 0 | III | 2 |
| 6 | 802659 | male | 55 | 2 | 1 | 0 | III | 3 |
| 7 | 606146 | female | 51 | 0 | 0 | 0 | 0 | 0 |
| 8 | 556330 | male | 66 | 3 | + | 0 | III | 1 |
| 9 | 537367 | female | 45 | 3 | 2 | x | III | 2 |
| 10 | 614705C | male | 55 | 3 | + | x | III | 3 |

**Table S2.** Antibodies, recombinant protein, and drugs information.

| Name | Supplier | Cat.no | Application |
|---|---|---|---|
| DDX21 | Santa Cruz Biotechnology | sc-376953 | 1:1000 |
| phosphorylated AKT | Cell Signaling Technology | 4060T | 1:1000 |
| Total AKT | ProteinTech | 10176-2-AP | 1:1000 |
| phosphorylated p38 | Cell Signaling Technology | 4511T | 1:1000 |
| Total p38 | Cell Signaling Technology | 8690T | 1:1000 |
| Phospho-ERK1-T202/Y204 + ERK2-T185/Y187 Rabbit mAb | Abclonal | AP0974 | 1:1000 |
| ERK1/2 Rabbit mAb | Abclonal | A4782 | 1:1000 |
| Ubi | Cell Signaling Technology | 3936T | 1:1000 |
| NUCKS1 | ProteinTech | 12023-2-AP | 1:1000 |
| Cyclin D1 | Abclonal | A19038 | 1:1000 |
| CDKN1B/Kip1 p27 | Santa Cruz Biotechnology | sc-1641 | 1:1000 |
| CDKN1A/p21CIP1 | Abclonal | A27846 | 1:1000 |
| PDL1 | Abclonal | A19135 | 1:1000 |
| Phosphorylated ATM | Cell Signaling Technology | D25E5 | 1:1000 |
| ATM | Cell Signaling Technology | 2873S | 1:1000 |
| RAD51 | Abclonal | A26856 | 1:1000 |
| p-Histone H2AX | Santa Cruz Biotechnology | sc-517348 | 1:1000 |
| caspase-3 | Santa Cruz Biotechnology | sc-56053 | 1:1000 |
| Cleaved-caspase3 | Cell Signaling Technology | 9661 | 1:1000 |
| Bcl-2 | Santa Cruz Biotechnology | sc-7382 | 1:1000 |
| Bax | ProteinTech | 50599-2-Ig | 1:1000 |
| Herc2 | Santa Cruz Biotechnology | sc-515891 | 1:1000 |
| Tubulin | Beyotime | AT819-1 | 1:1000 |
| Lamin A/C | Abclonal | A19524 | 1:50000 |
| HA-Tag(26D11) mAb | Abmart | M20003S | 1:5000 |
| anti DDDDK-Tag pAb | ABclonal | AE004 | 1:2000 |
| VeriBlot for IP Detection Reagent (HRP) | Abcam | ab131366 | 1:3000 |
| HRP-labeled Goat Anti-Mouse IgG(H+L) | Beyotime | A0216 | 1:3000 |
| HRP-labeled Goat Anti-Rabbit IgG(H+L) | Beyotime | A0208 | 1:3000 |
| anti-CD3 | BioLegend | 300438 | 5 μg/mL |
| anti-CD28 | BioLegend | 302934 | 5 μg/mL |
| recombinant human IL-2 | ABclonal | RP01039 | 100 U/mL |
| MG132 | MedChemExpress | HY-13259 | 10 μM |
| Cyclohexamide | MedChemExpress | HY-12320 | 200 μg/ml |
| SC79 | TargetMol | T2274 | 10 μM |
| Anisomycin | TargetMol | T6758 | 10 nM |
| MK-2206 | TargetMol | T1952 | 10 μM |
| WAY-600 | TargetMol | T6730 | 3 μM |
| SB202190 | MedChemExpress | HY-10295 | 10 μM |
| SB203580 | MedChemExpress | HY-10256 | 10 μM |

**Table S3.** The primer sequences of qRT-PCR.

| Gene | Primer sequences (5' to 3') |
|---|---|
| DDX21 | F: AATGTTGCTGCACGTGGGTTAG<br>R: CCCGGATCGATGAATGTAGGAC |
| ACTB | F: AGGATTCCTATGTGGGCGAC<br>R: GATAGCACAGCCTGGATAGCAA |
| CDKN1A（p21) | F: GCGACTGTGATGCGCTAATG<br>R: GTGGTAGAAATCTGTCATGCTGG |
| NUCKS1 | F: GGCCTGTCAGAAATAGGAAGGT<br>R: TTTAGCTTCTCGGGGAGATGAT |
| HERC2 | F: GCGCTGTCTTTTGCCTTTG<br>R: AGGAACCTGGTCGCTCTCTC |

**Table S4.** Small interfering RNA sequences.

| Gene | Primer sequences (5' to 3') |
|---|---|
| DDX21#1 | GCGGAGTTTCAGTAAAGCATT |
| DDX21#2 | CGCTCCTTGATCAACTCAAAT |
| NUCKS1 | GTTGTTGATTACTCACAGTTT |
| CDKN1A（p21) | CGCTCTACATCTTCTGCCTTA |
| HERC2 | CCUGGCAUAAUGGAGUCAUUU |

**Table S5.** Differential expression genes between si-NC and si-DDX21 in CRC cells.

| Cell type | Gene Name | si-DDX21 | si-NC | log2FoldChange | Fold Change | *P* value | Regulated type |
|---|---|---|---|---|---|---|---|
| P-HCT116 | DDX21 | 769 | 8020 | -3.567402433504 | 0.084353841 | 5.53E-54 | down |
| | **NUCKS1** | 2622 | 10841 | -2.2327848 | 0.212747665 | 3.19E-25 | down |
| | HMGB1 | 4789 | 14392 | -1.772526574 | 0.292695694 | 5.14E-17 | down |
| | KRT7 | 54 | 247 | -2.375697944 | 0.192683115 | 3.54E-16 | down |
| | RDX | 1916 | 5564 | -1.72303921 | 0.302909932 | 5.53E-16 | down |
| | PIP4K2A | 1279 | 3233 | -1.52284359 | 0.347999324 | 9.33E-13 | down |
| | TOP2A | 3347 | 8247 | -1.486048156 | 0.356989079 | 1.62E-12 | down |
| | MYCBP | 192 | 418 | -1.306885455 | 0.404192525 | 6.01E-08 | down |
| | TMOD2 | 233 | 512 | -1.320404317 | 0.400422704 | 2.30E-08 | down |
| | SULT2B1 | 4 | 32 | -3.143157205 | 0.113191913 | 8.97E-07 | down |
| | HIST1H2BK | 57 | 142 | -1.499766857 | 0.35361053 | 7.63E-07 | down |
| | C2orf76 | 55 | 118 | -1.284312496 | 0.41056641 | 3.94E-05 | down |
| | TPM4 | 9954 | 1789 | 2.290963771 | 4.893829268 | 3.06E-26 | up |
| | ADAM15 | 3282 | 996 | 1.535162441 | 2.898210616 | 7.61E-13 | up |
| | SCG2 | 373 | 99 | 1.7274015 | 3.311308669 | 3.69E-11 | up |
| | TSC22D1 | 7052 | 2319 | 1.41939992 | 2.674742335 | 1.61E-11 | up |
| P-DLD-1 | DDX21 | 1712 | 12696 | -3.139648916 | 0.113467504 | 5.42E-45 | down |
| | **NUCKS1** | 3693 | 13032 | -2.068283864 | 0.238442967 | 3.59E-22 | down |
| | TOP2A | 4128 | 13377 | -1.945335515 | 0.259654383 | 5.86E-20 | down |
| | RDX | 1282 | 4307 | -1.997303463 | 0.250467711 | 1.83E-20 | down |
| | SULT2B1 | 112 | 322 | -1.771456824 | 0.292912807 | 7.15E-12 | down |
| | HMGB1 | 9299 | 19820 | -1.340927643 | 0.394766742 | 1.27E-10 | down |
| | HIST1H2BK | 184 | 427 | -1.462983012 | 0.362742323 | 1.47E-09 | down |
| | MYCBP | 317 | 699 | -1.389558531 | 0.38168158 | 1.26E-09 | down |
| | TMOD2 | 37 | 110 | -1.817250641 | 0.283761223 | 5.42E-08 | down |
| | C2orf76 | 35 | 97 | -1.715873327 | 0.304418232 | 5.03E-07 | down |
| | PIP4K2A | 668 | 1152 | -1.035201488 | 0.487947729 | 2.11E-06 | down |
| | KRT7 | 7 | 32 | -2.419203579 | 0.186959336 | 4.17E-05 | down |
| | SCG2 | 12 | 30 | -1.560311297 | 0.33907791 | 0.004744033 | down |
| | ADAM15 | 4217 | 1740 | 1.027950537 | 2.039125453 | 1.00E-06 | up |
| | TSC22D1 | 3088 | 1282 | 1.019080458 | 2.02662682 | 1.44E-06 | up |
| | TPM4 | 10641 | 2573 | 1.798933817 | 3.479629781 | 2.42E-17 | up |
