# Supplementary material for: Ubiquitination of DDX21 by HERC2 induces a dormancy-like phenotype via the NUCKS1-p21/p27 axis to promote radio-resistance in colorectal cancer cells
Source: Cell Death Dis. 2026 May 3;17(1):588. doi: 10.1038/s41419-026-08811-0 (PMC13284300; doi:10.1038/s41419-026-08811-0)

Uncropped original western blots

Western blots in the article | Original uncropped western

Fig. 3A

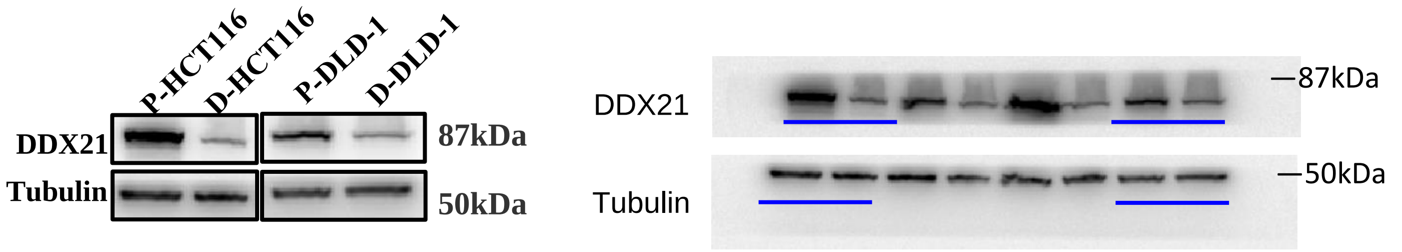

Fig. 3E

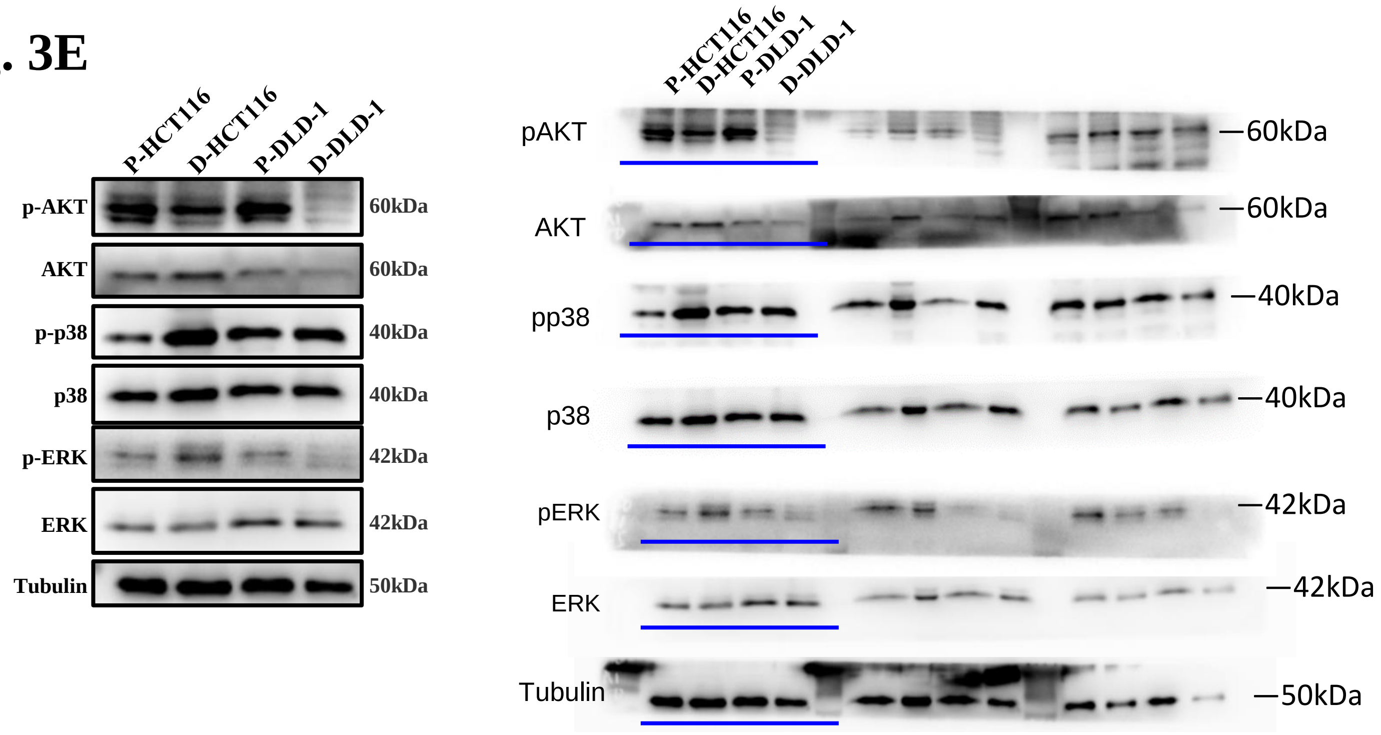

Fig. 3F

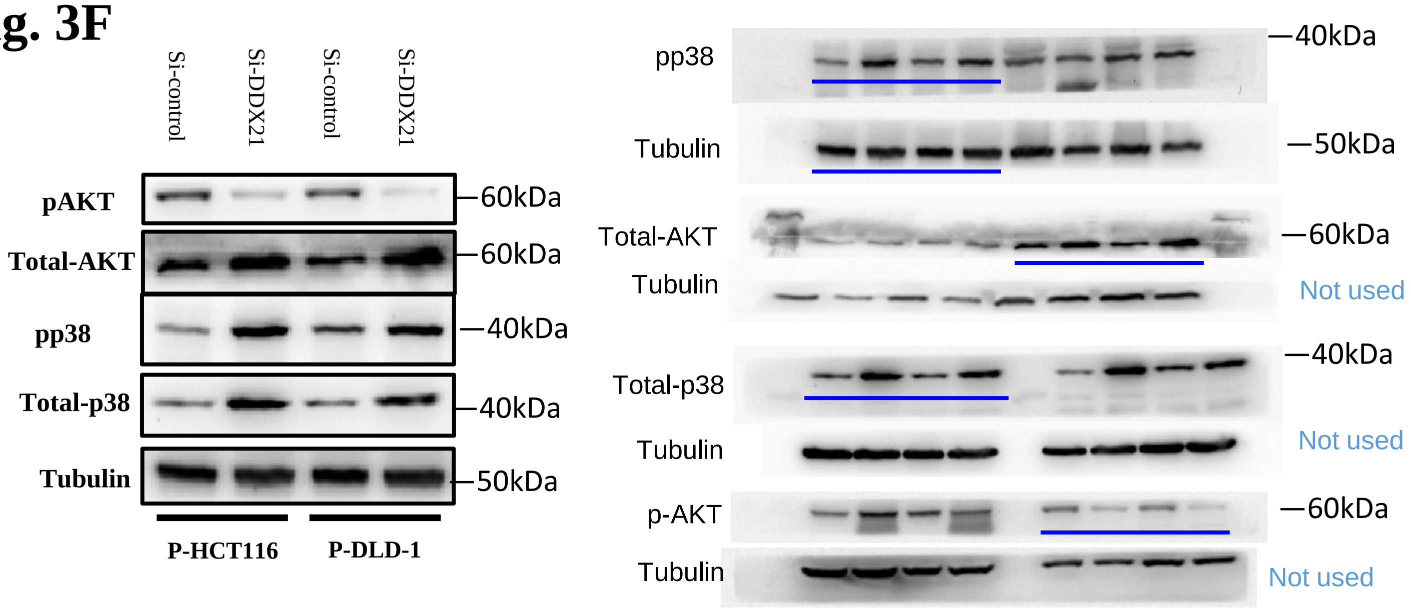

Western blots in the article

Original uncropped western

Fig. 3G

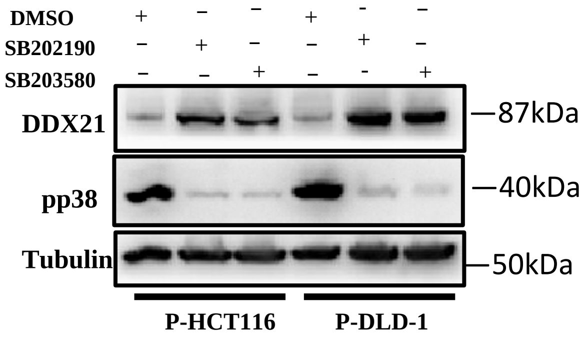

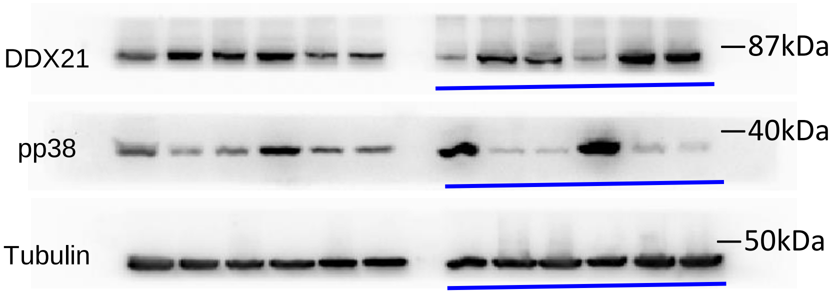

Fig. 3H

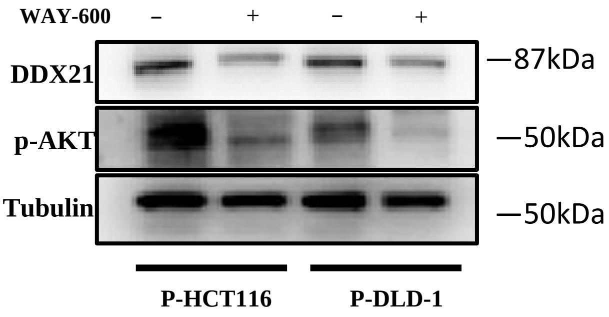

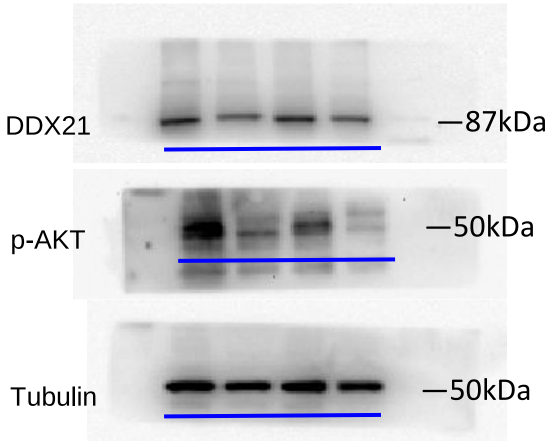

Western blots in the article

Original uncropped western

Fig. 4D

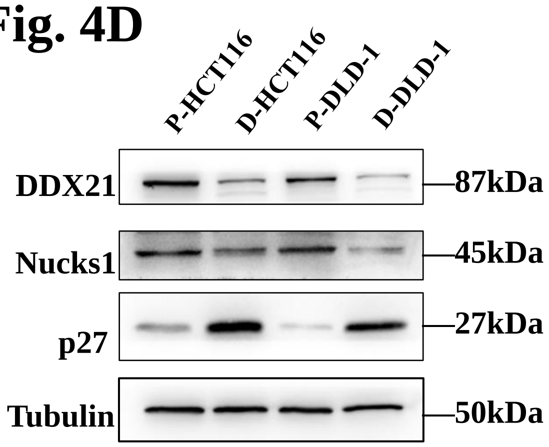

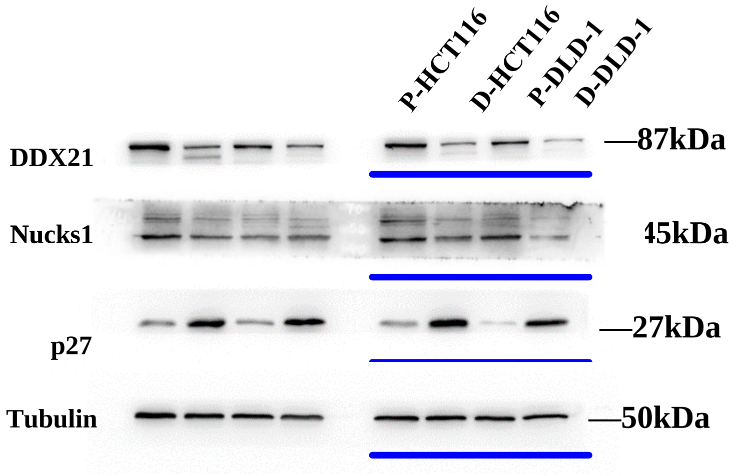

Fig. 4E

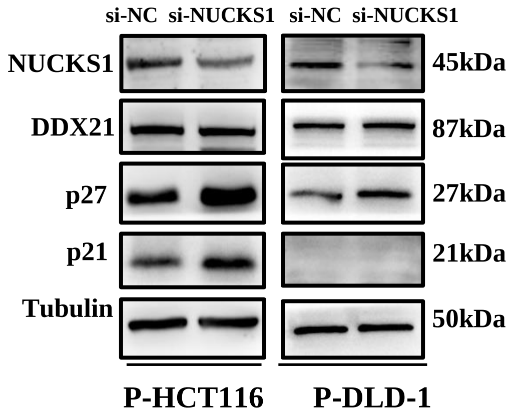

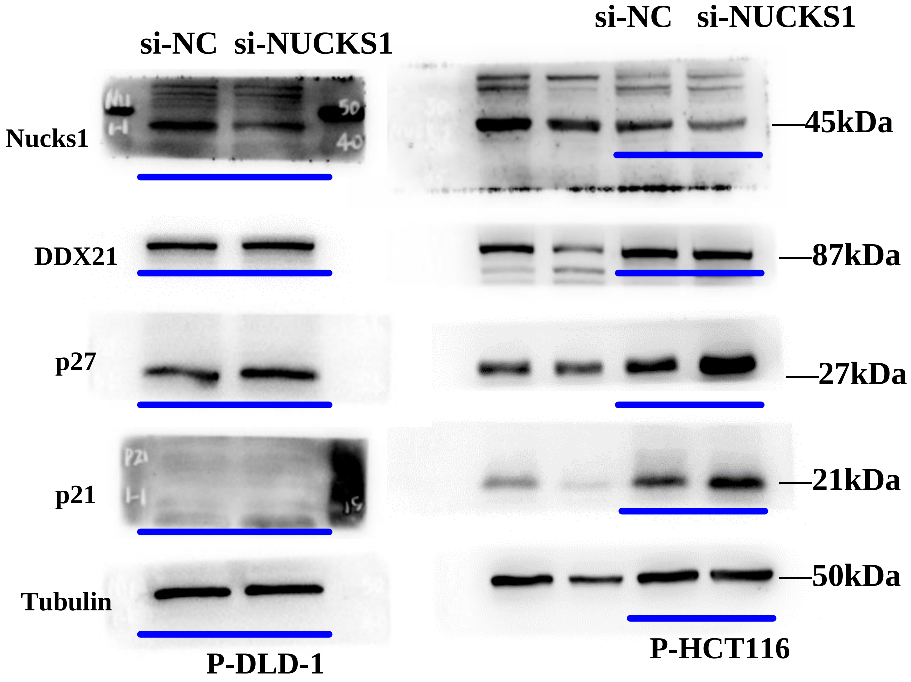

Fig. 4F

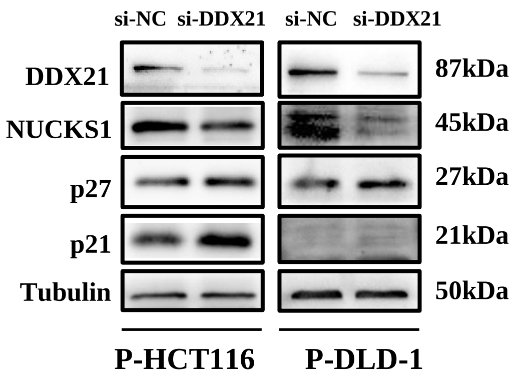

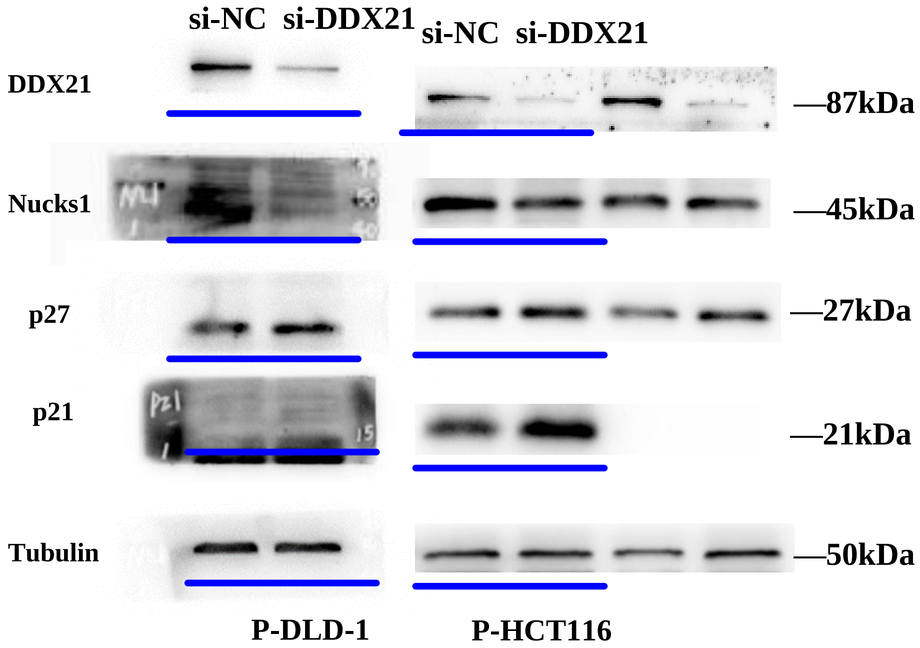

Western blots in the article

Original uncropped western

Fig. 4H

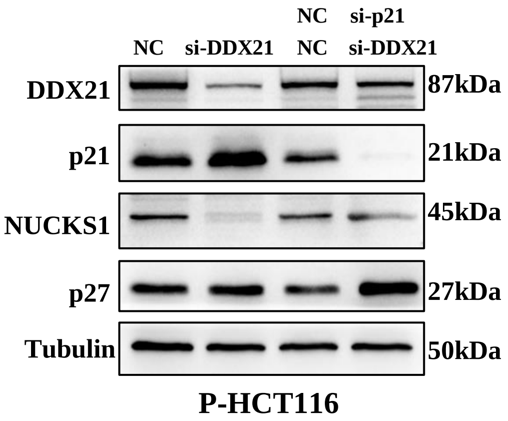

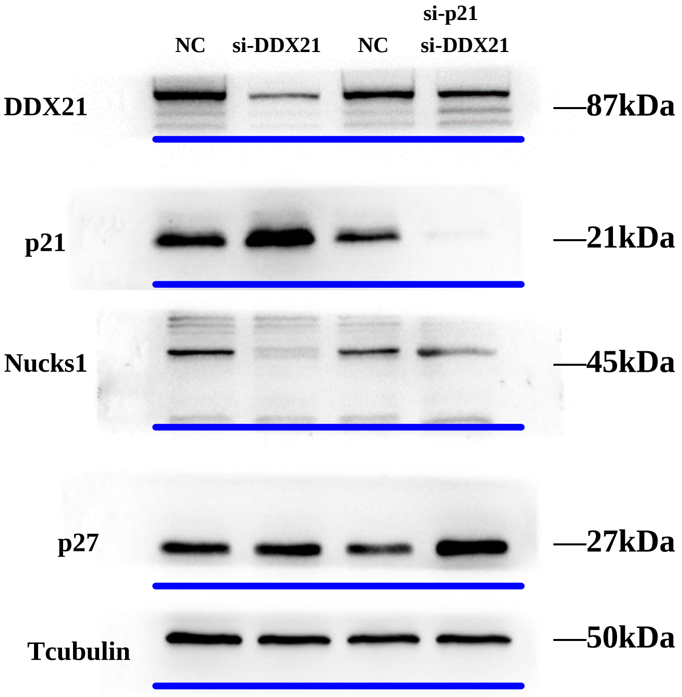

Fig. 4I

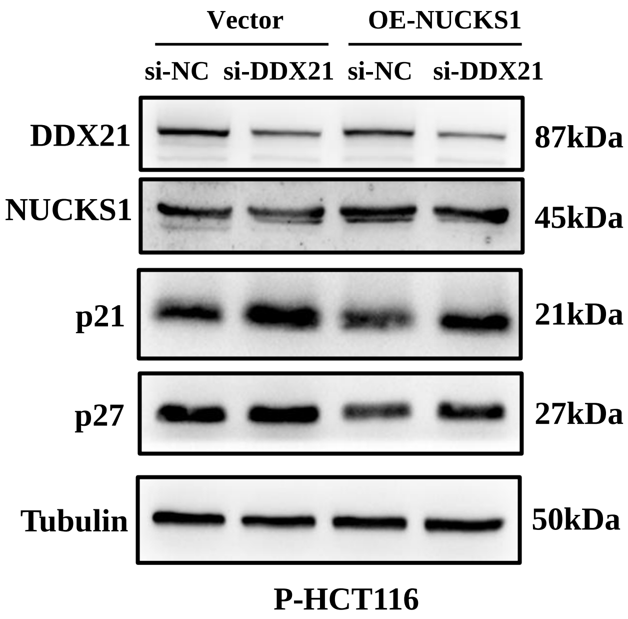

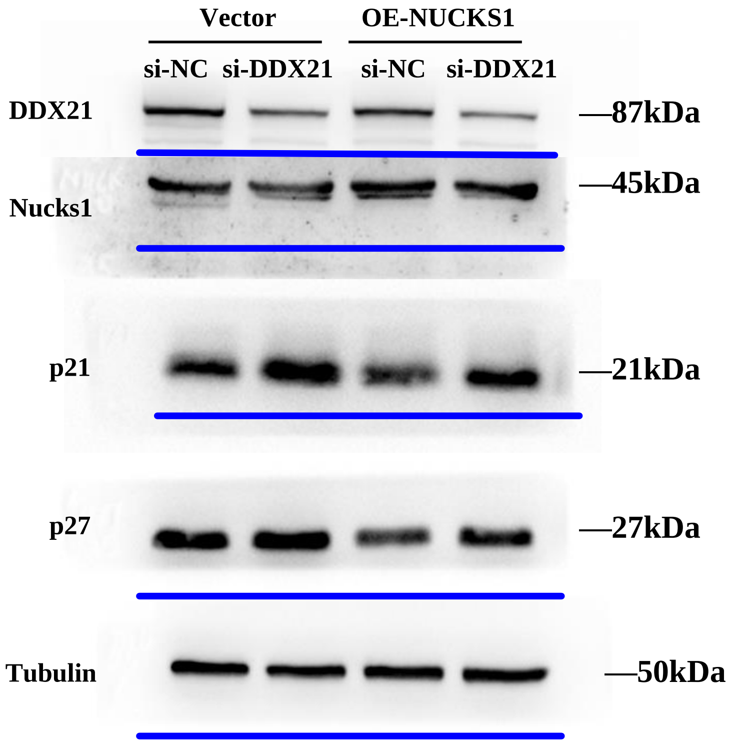

Western blots in the article

Original uncropped western

Fig. 4I

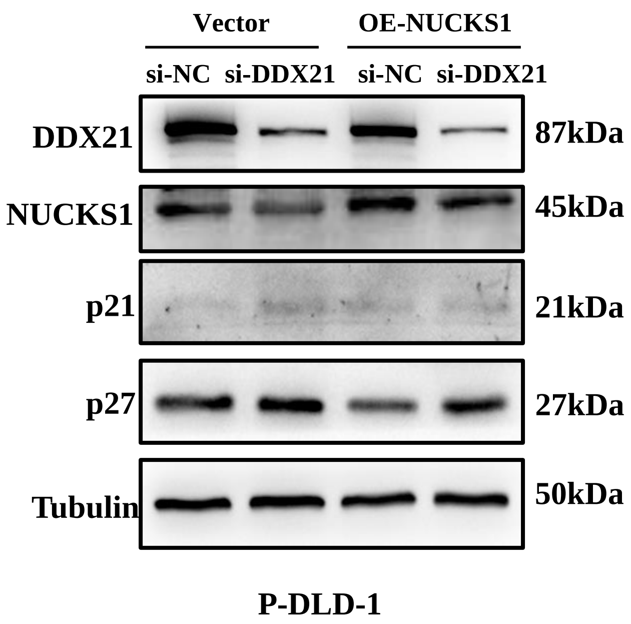

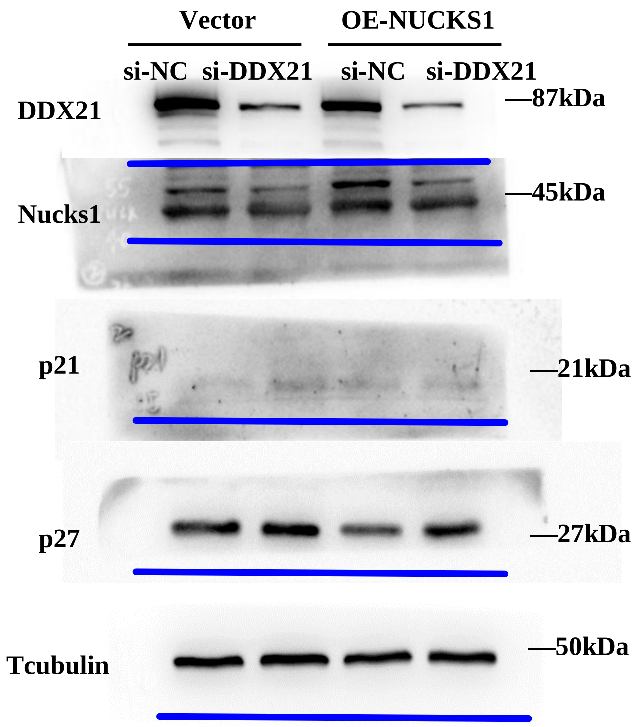

Fig. 5B

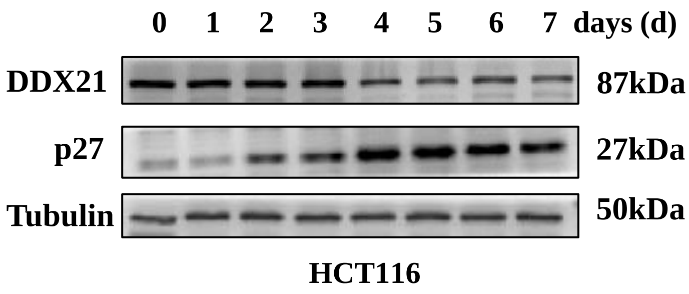

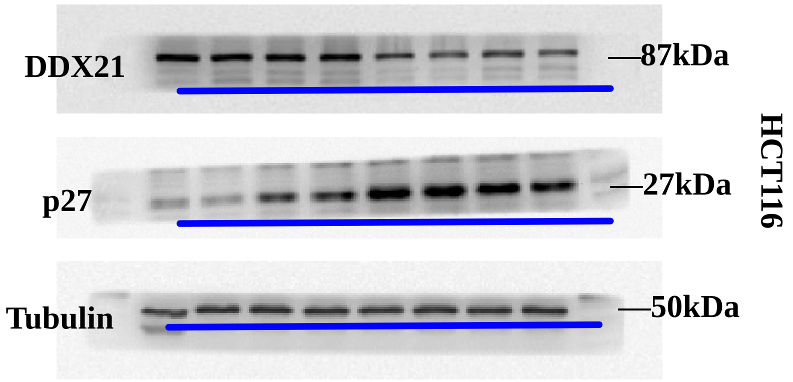

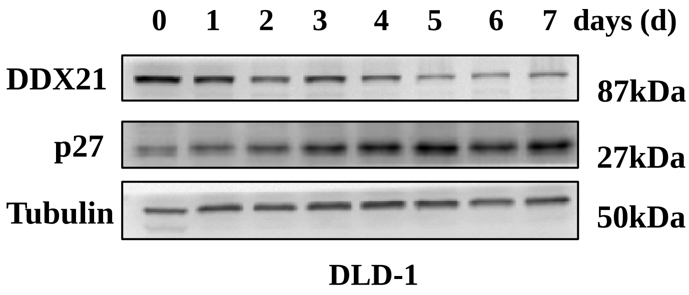

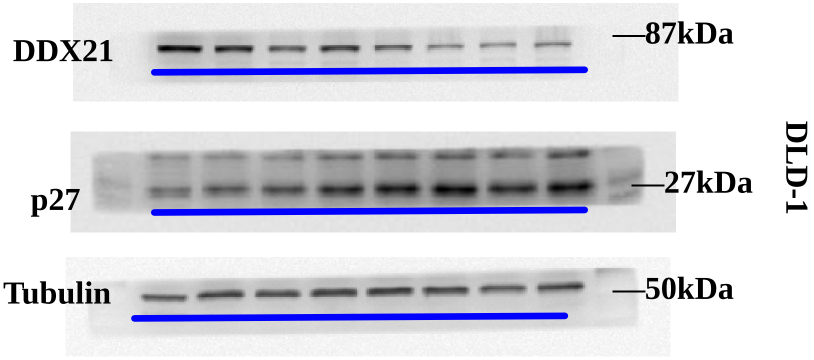

Western blots in the article

Original uncropped western

Fig. 5C

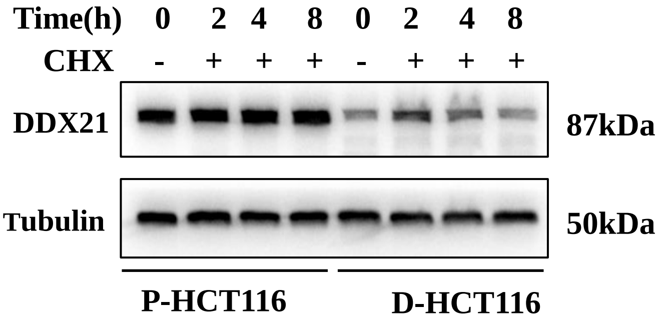

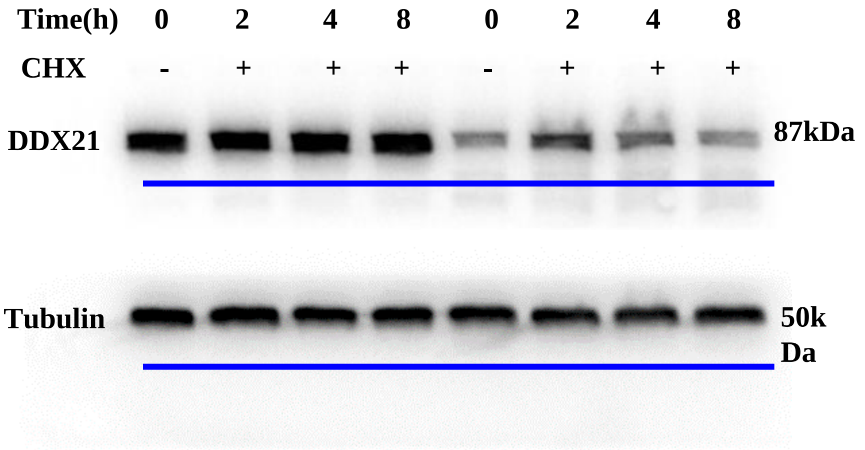

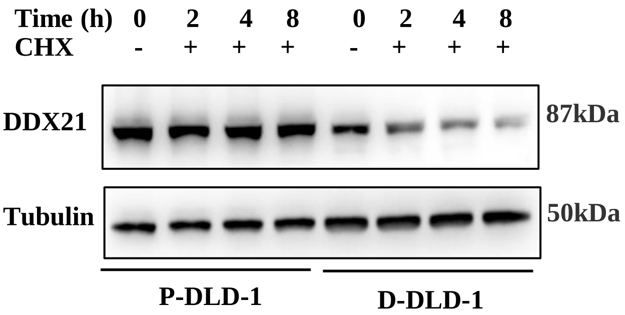

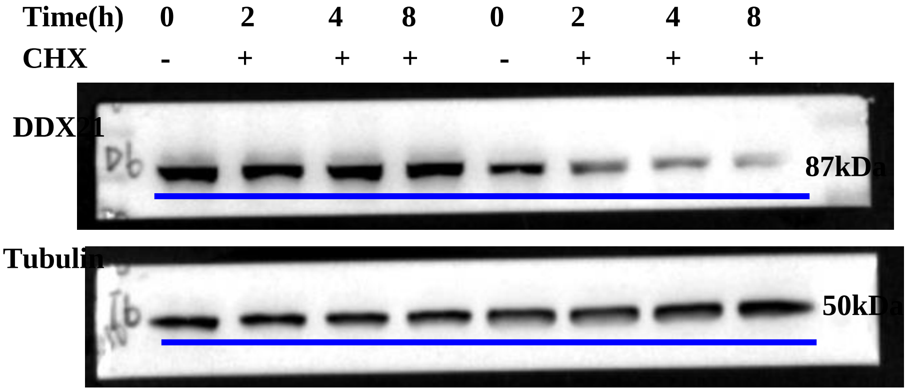

Fig. 5D

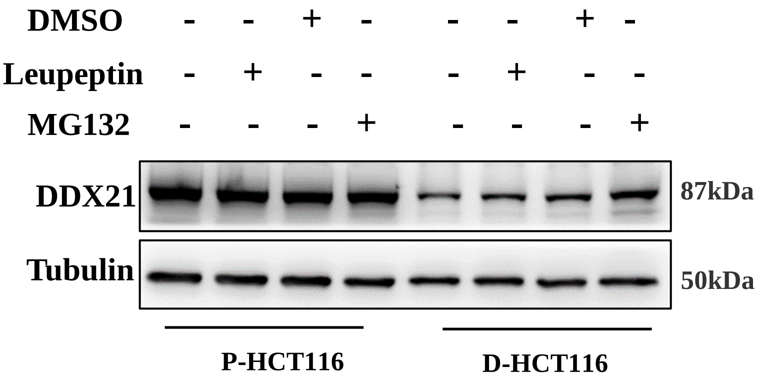

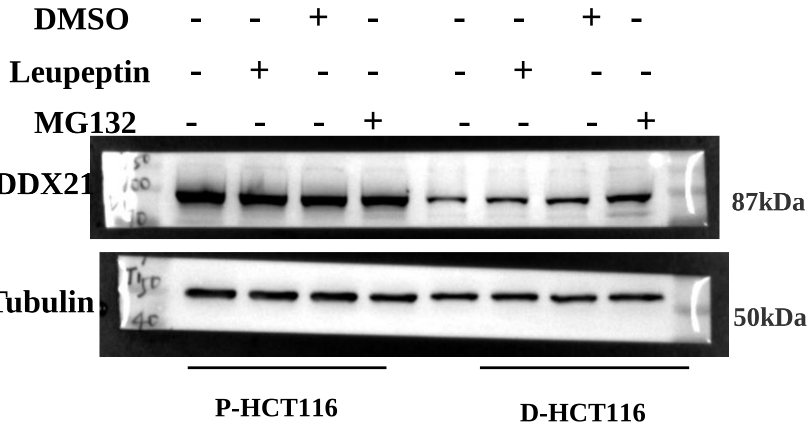

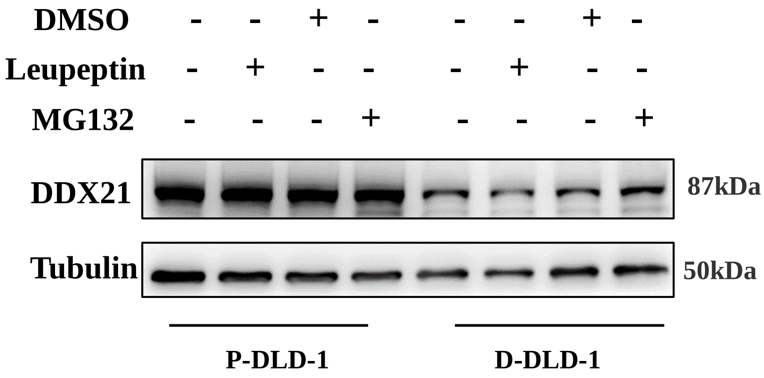

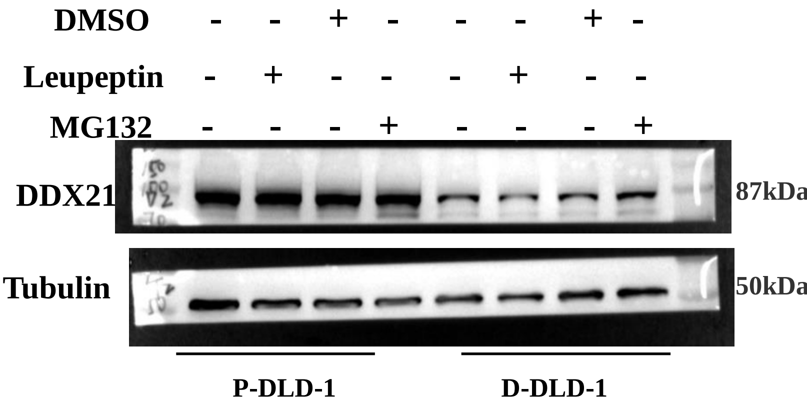

Western blots in the article

Original uncropped western

Fig. 5E

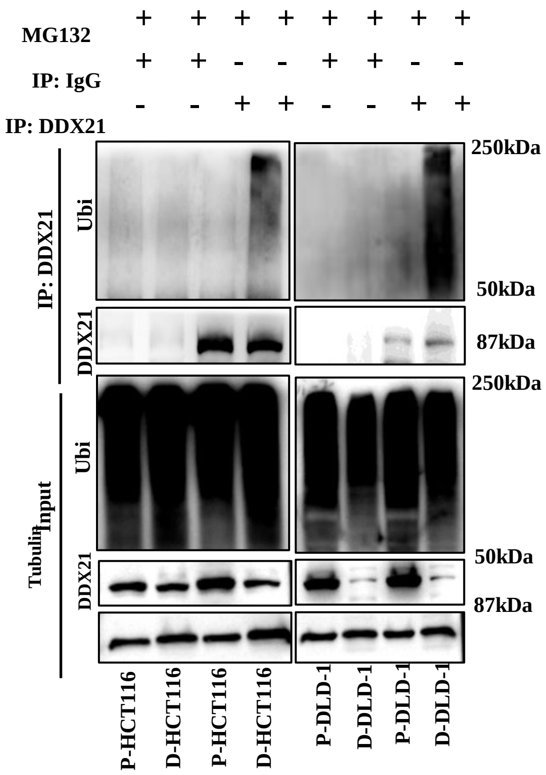

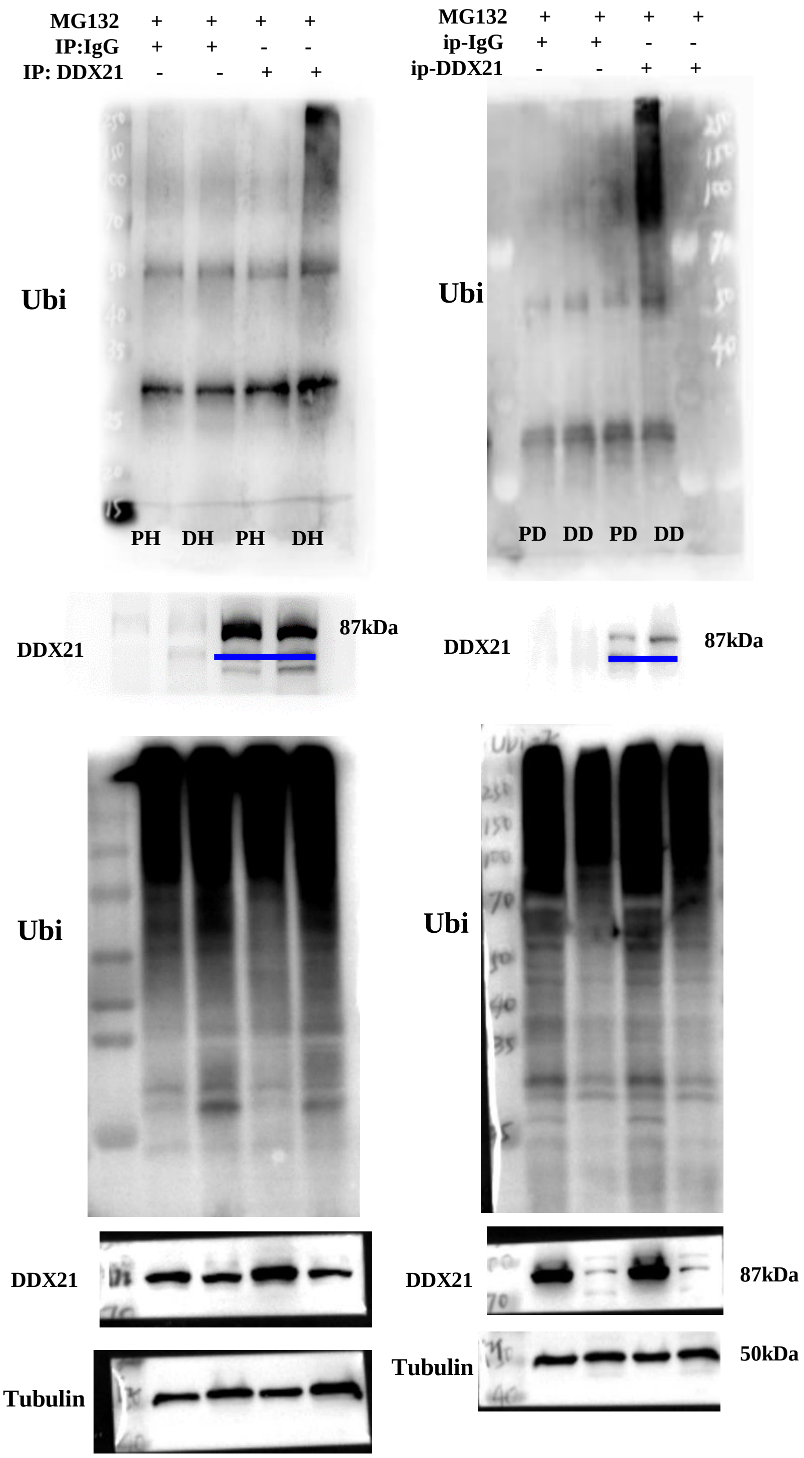

Western blots in the article

Original uncropped western

Fig. 6C

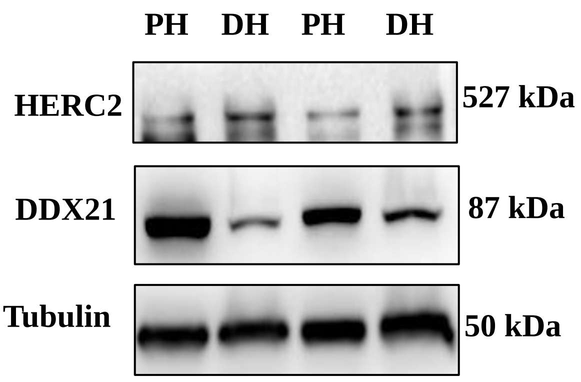

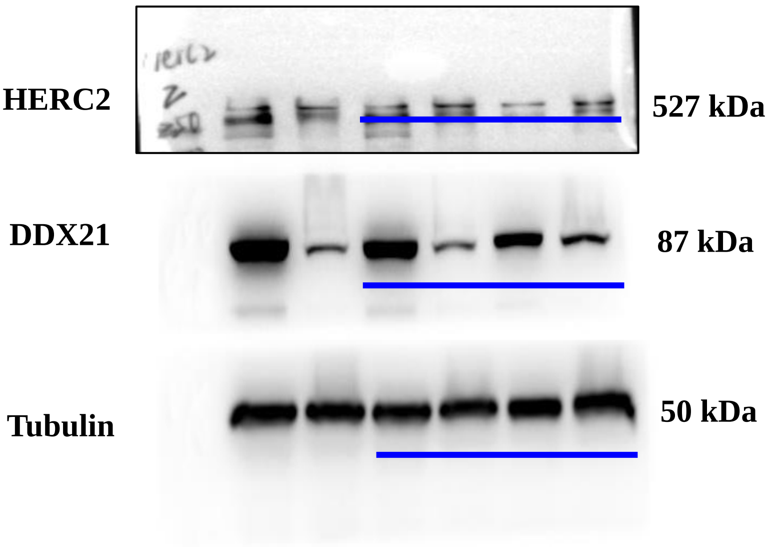

Fig. 6D

Western blots in the article

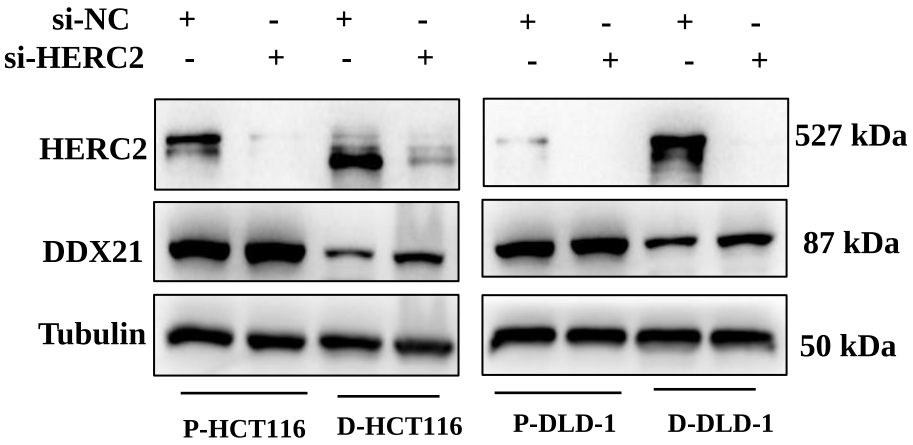

Original uncropped western

Original uncropped western

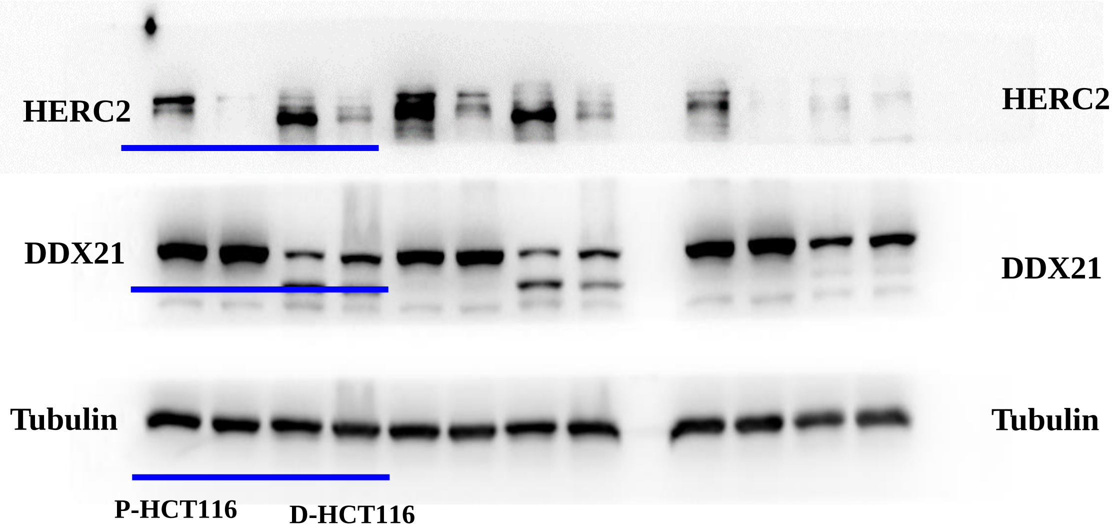

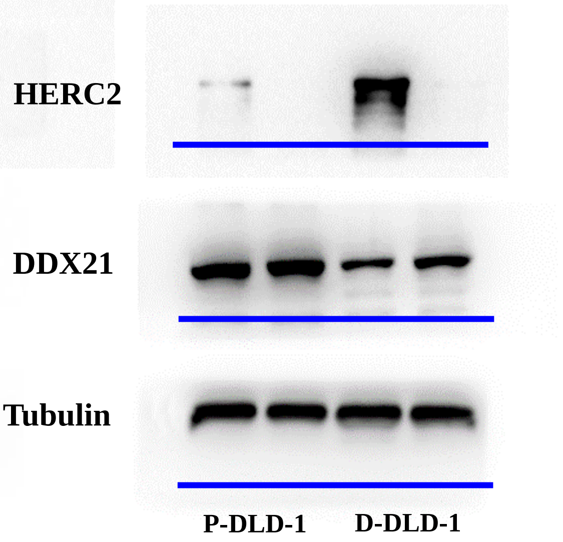

Western blots in the article

Original uncropped western

Fig. 6F

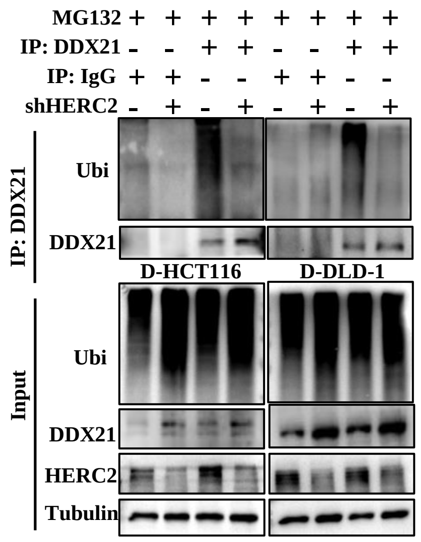

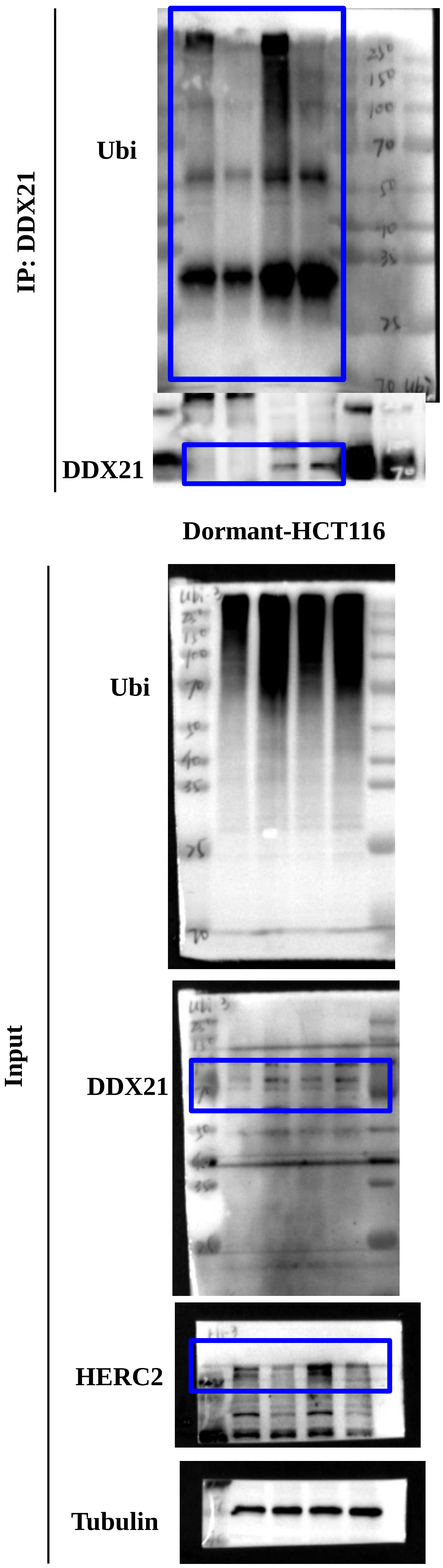

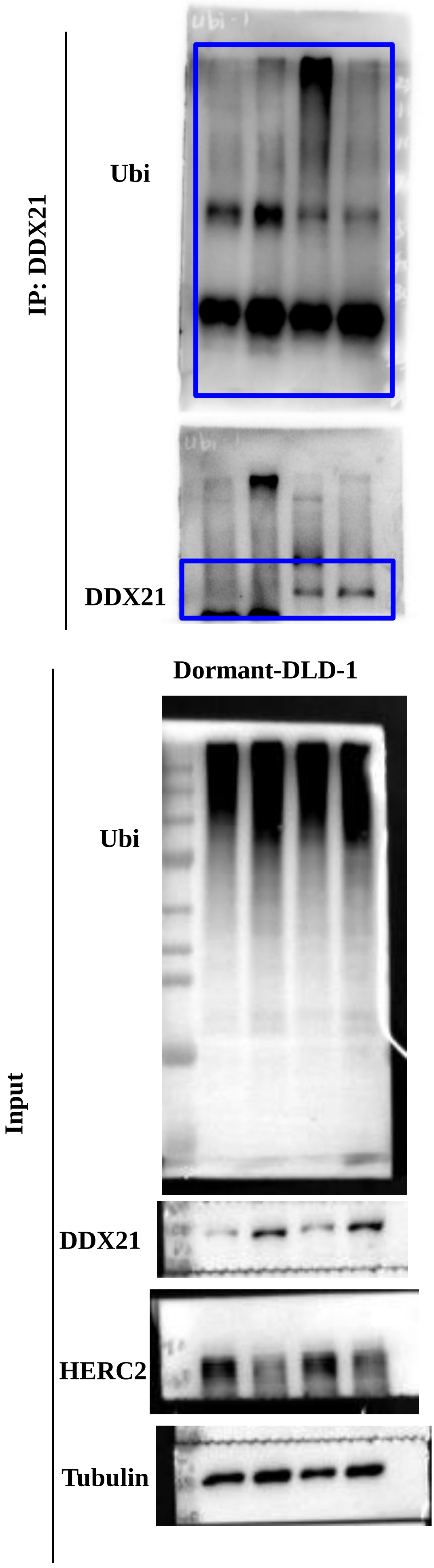

Fig. 6G

Western blots in the article

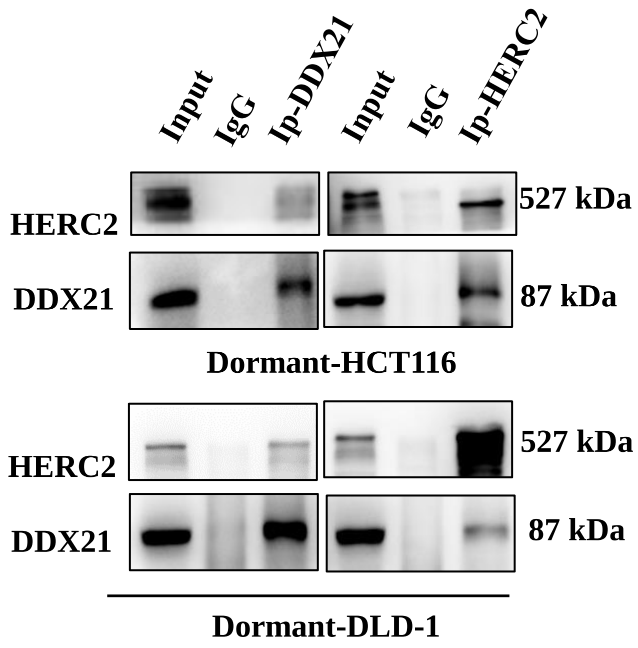

Original uncropped western

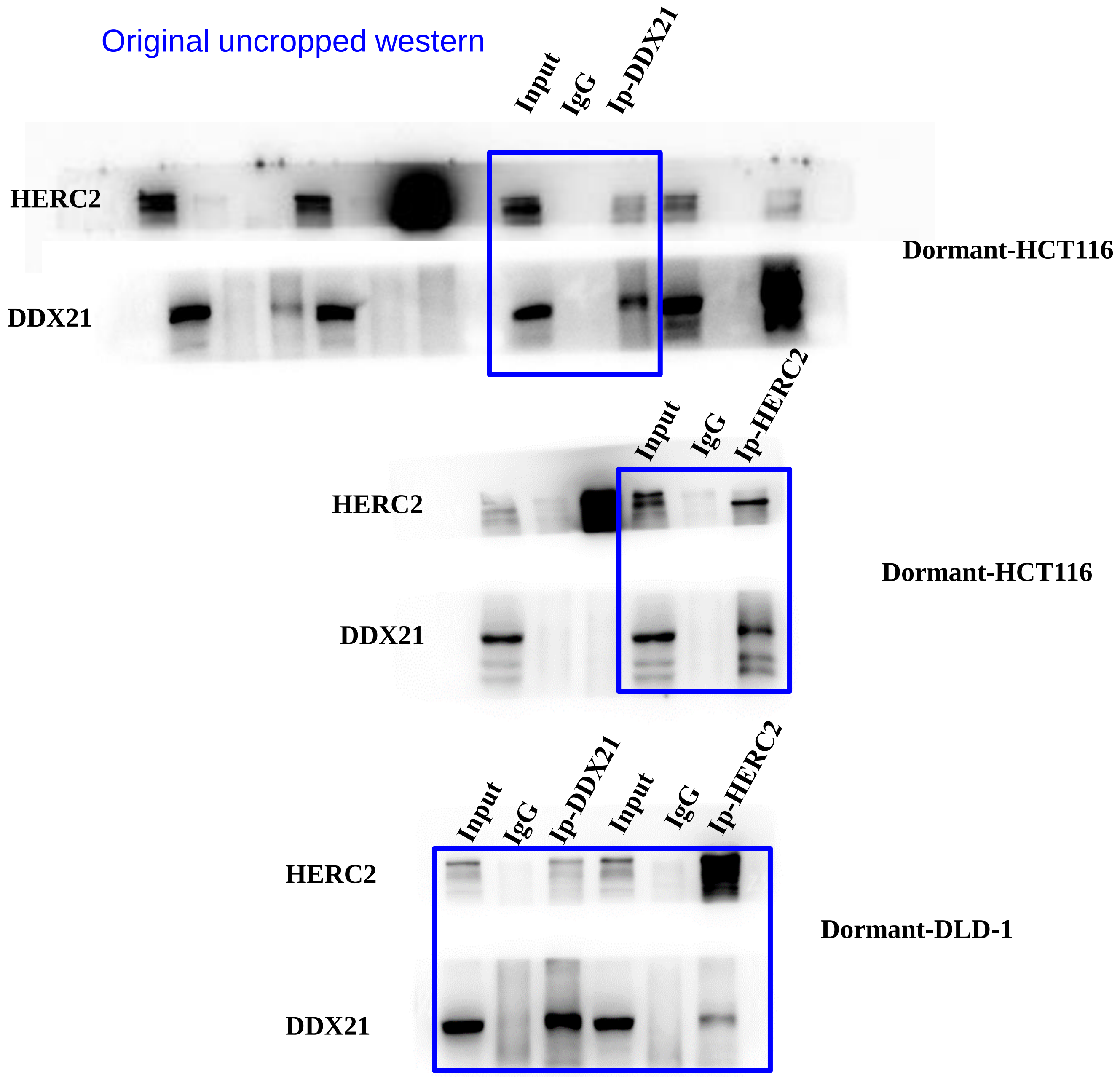

Fig. 6H

Western blots in the article

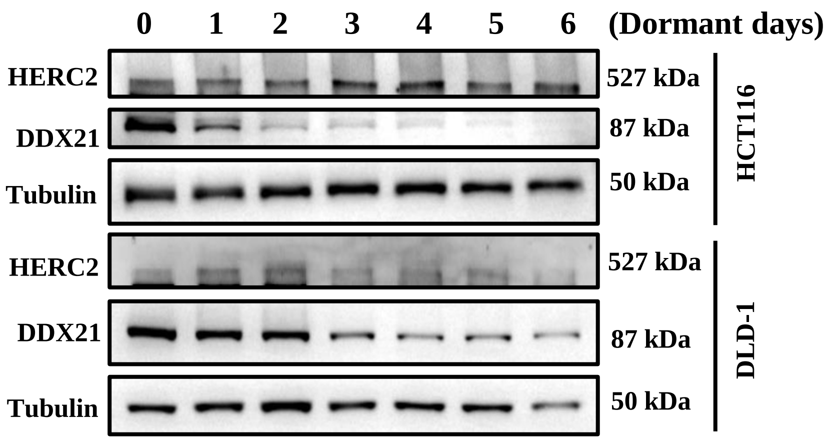

Original uncropped western

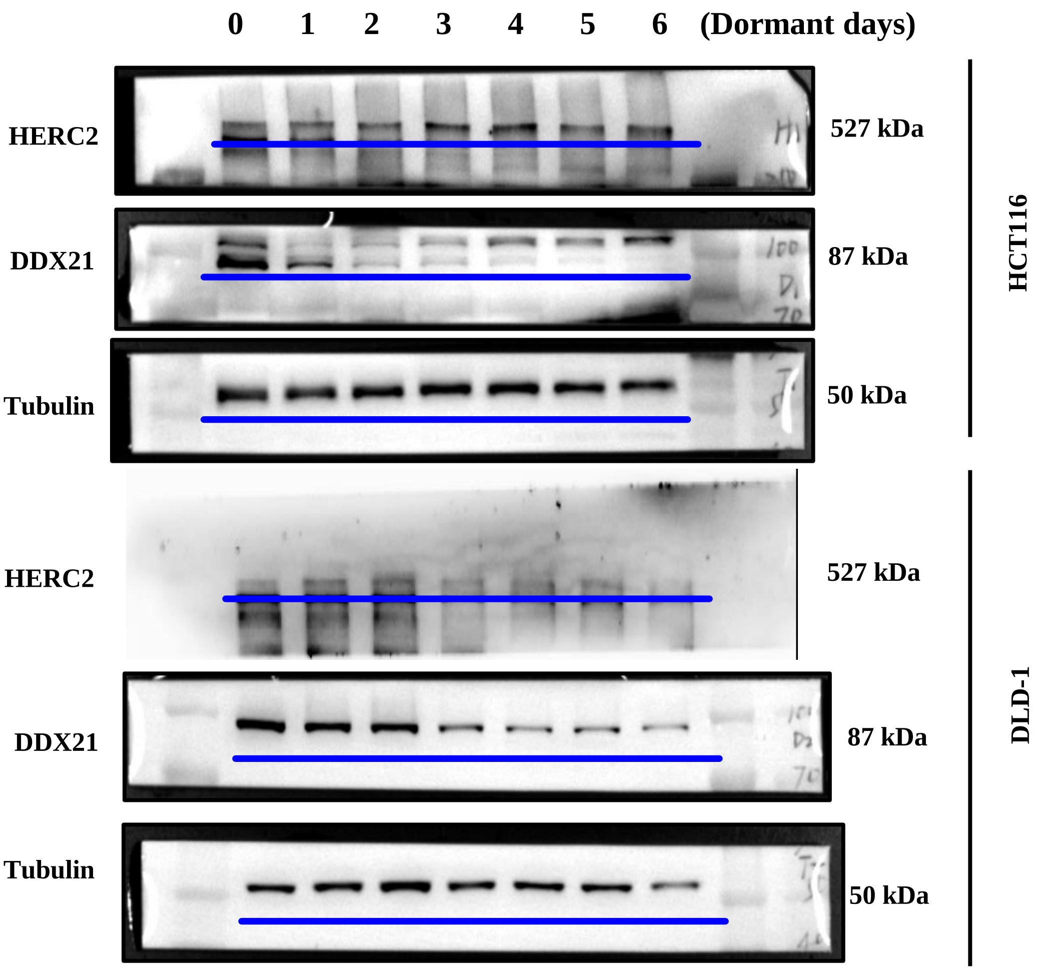

Fig. 6K

Western blots in the article

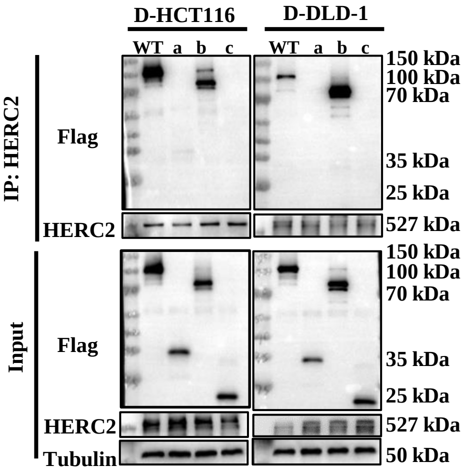

Original uncropped western

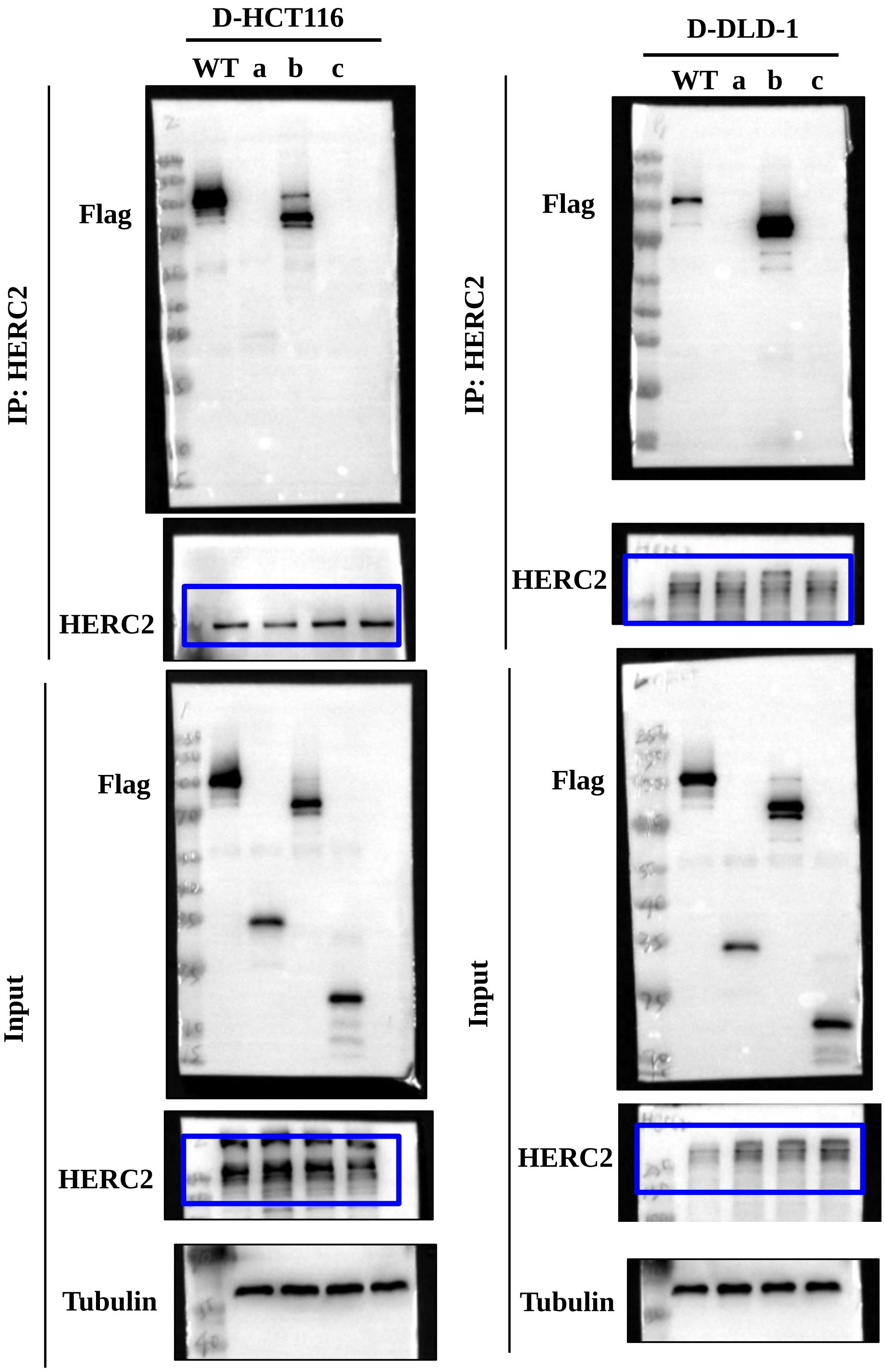

Fig. 6L

Original uncropped western

Western blots in the article

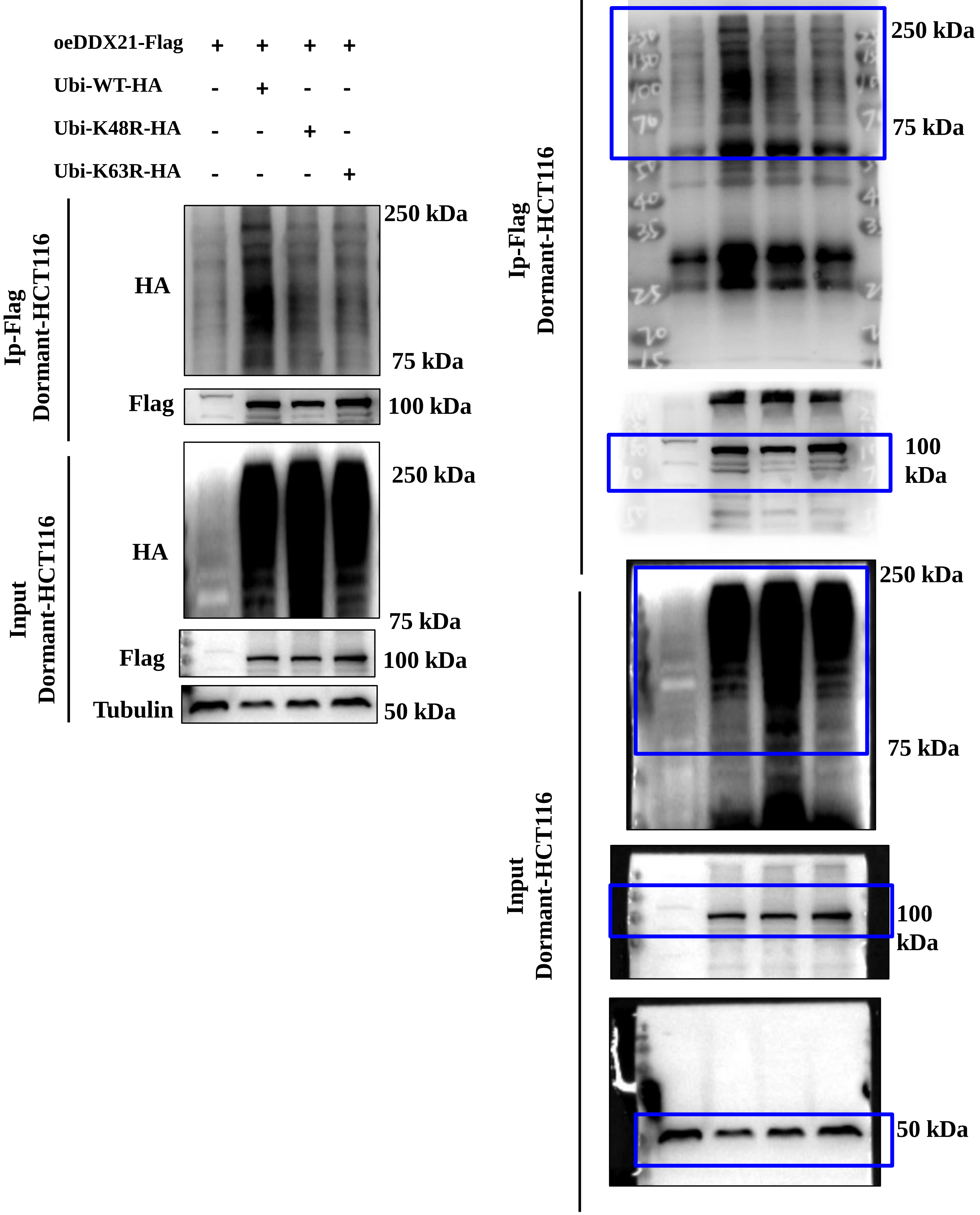

Fig. 6L

Western blots in the article

Original uncropped western

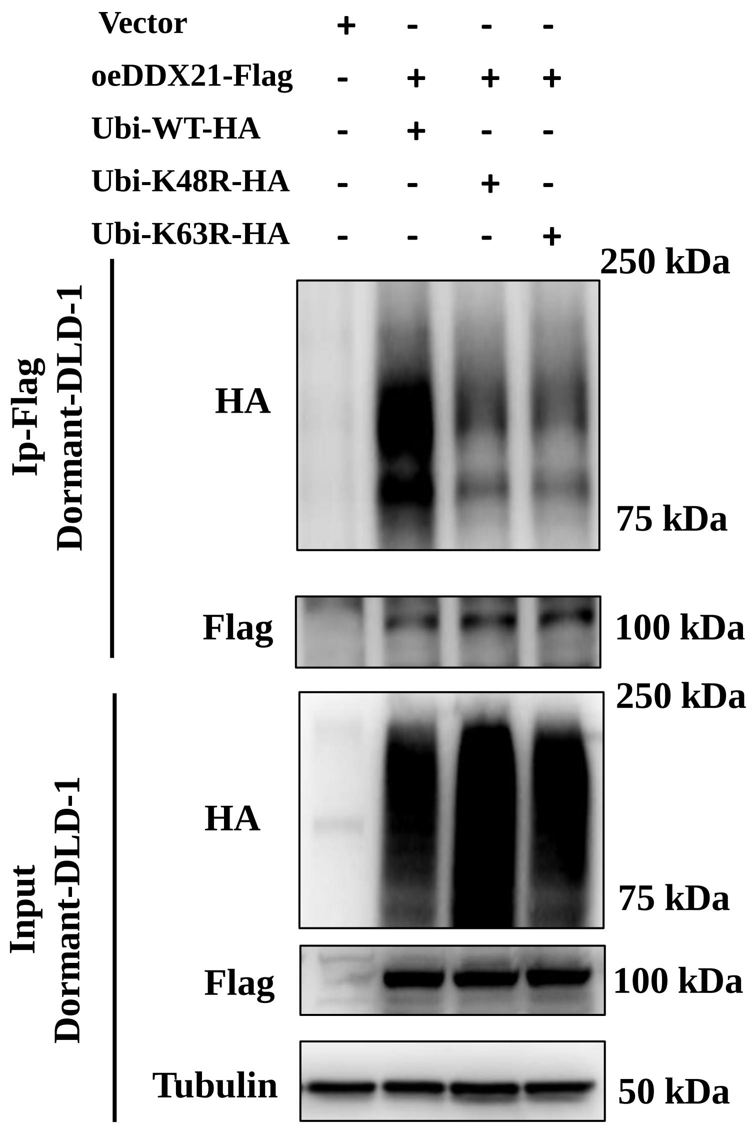

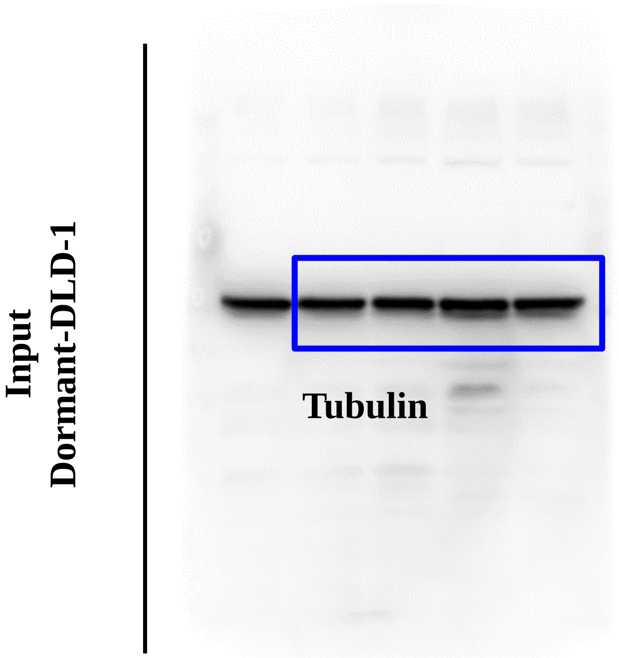

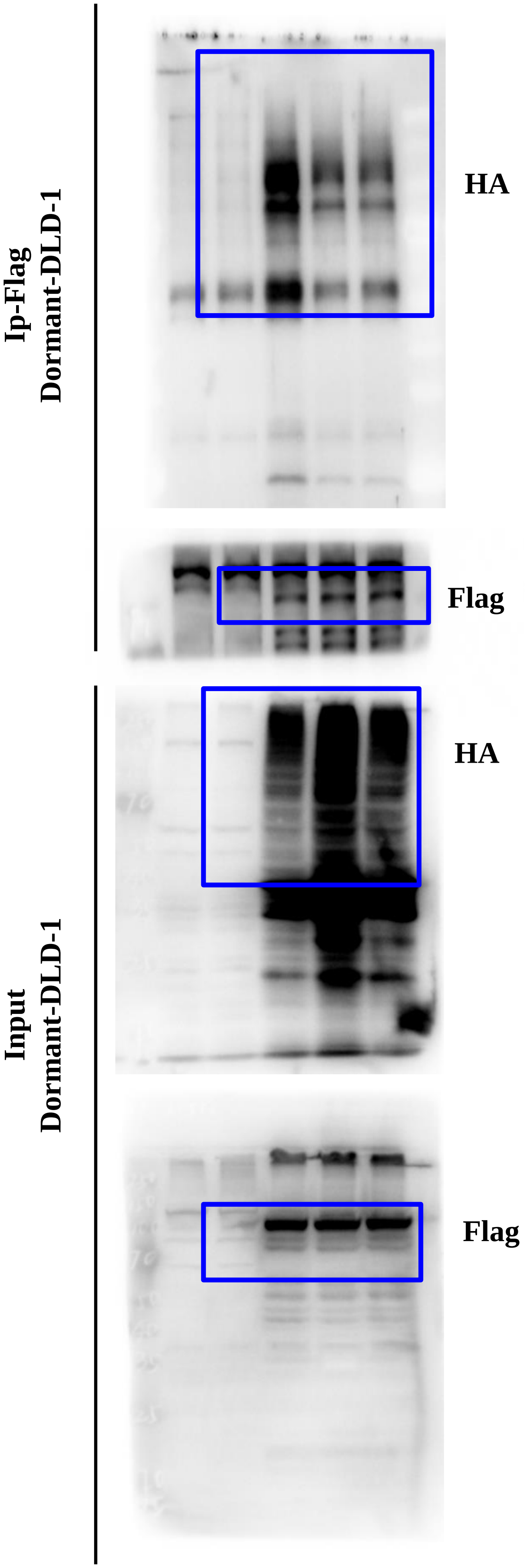

Fig. 7D

Western blots in the article

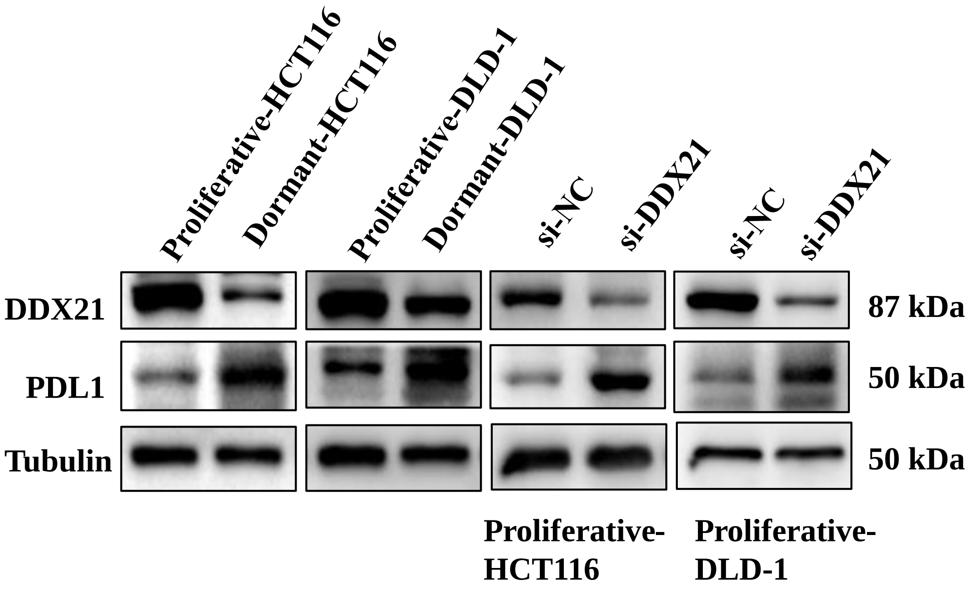

Original uncropped western

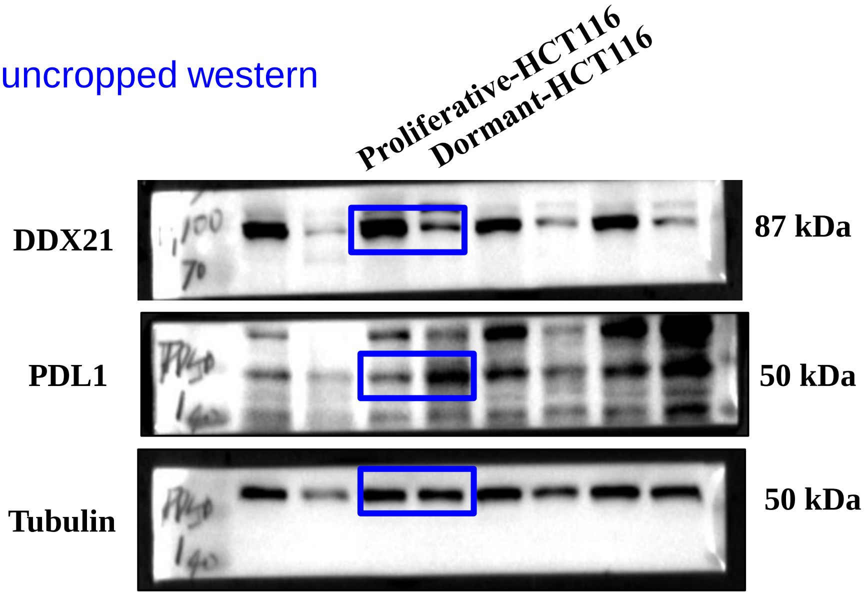

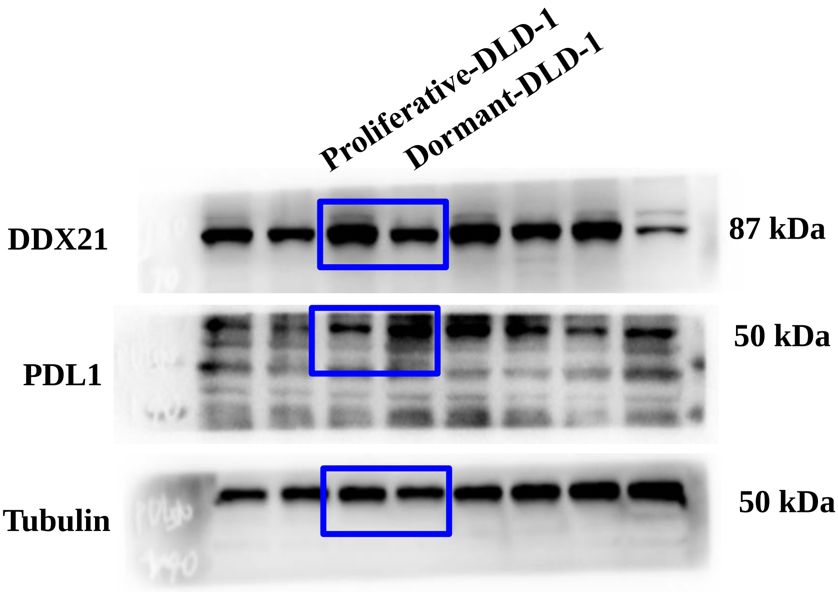

Western blots in the article

Fig. 7D

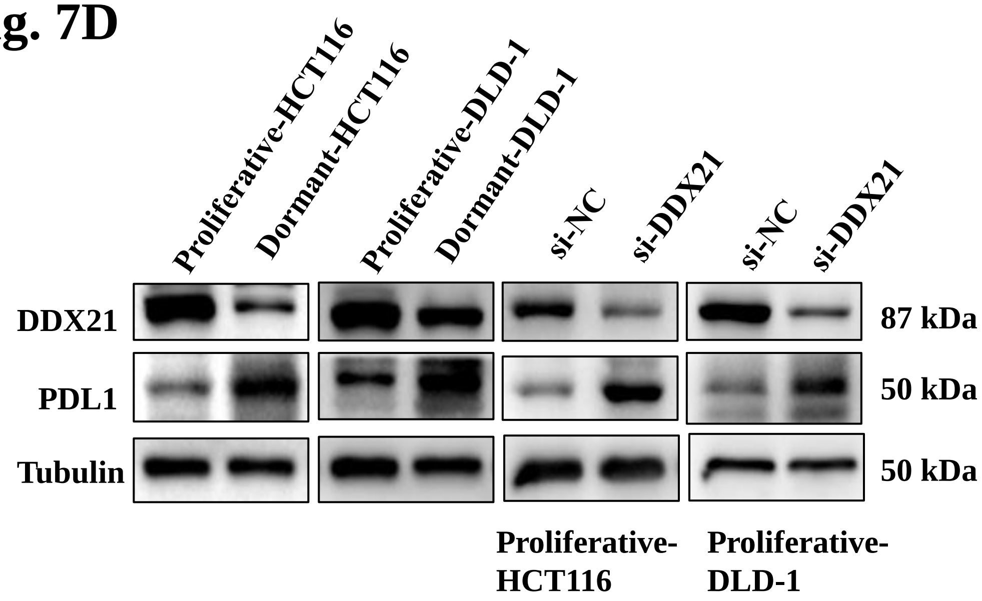

Original uncropped western

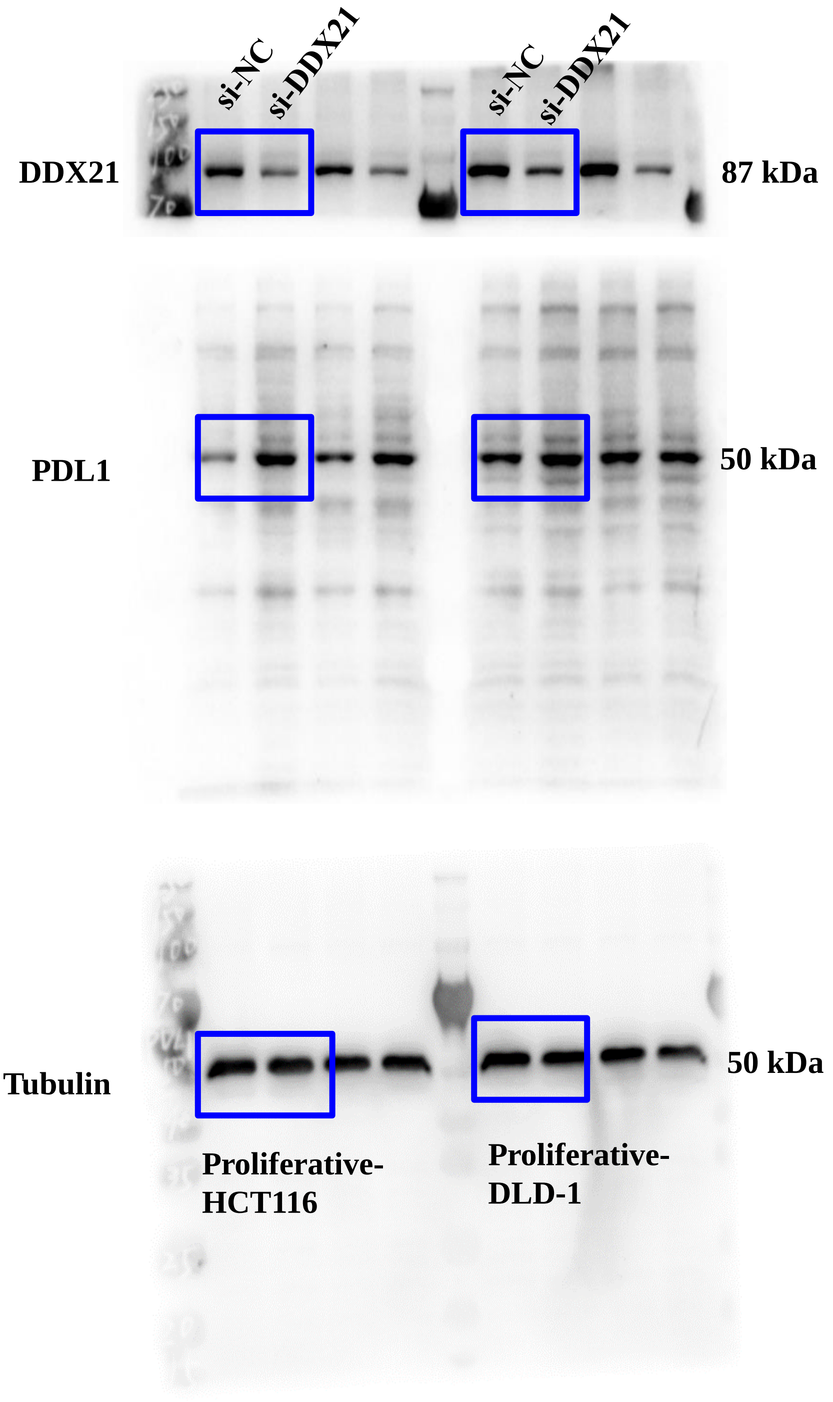

Fig. 7J

Western blots in the article

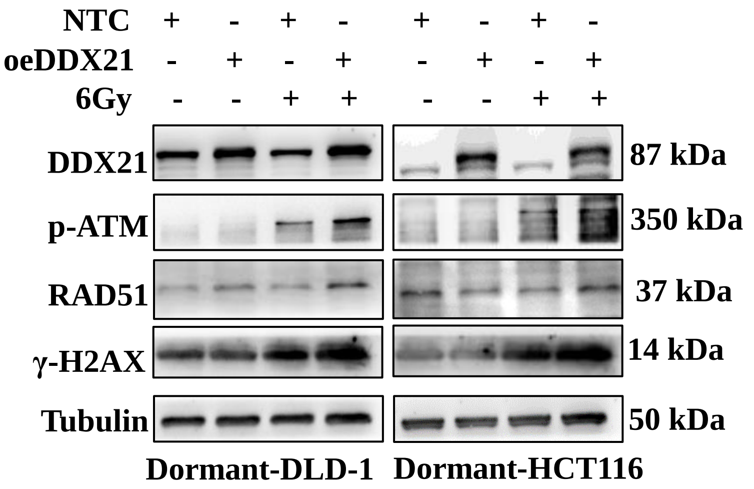

Original uncropped western

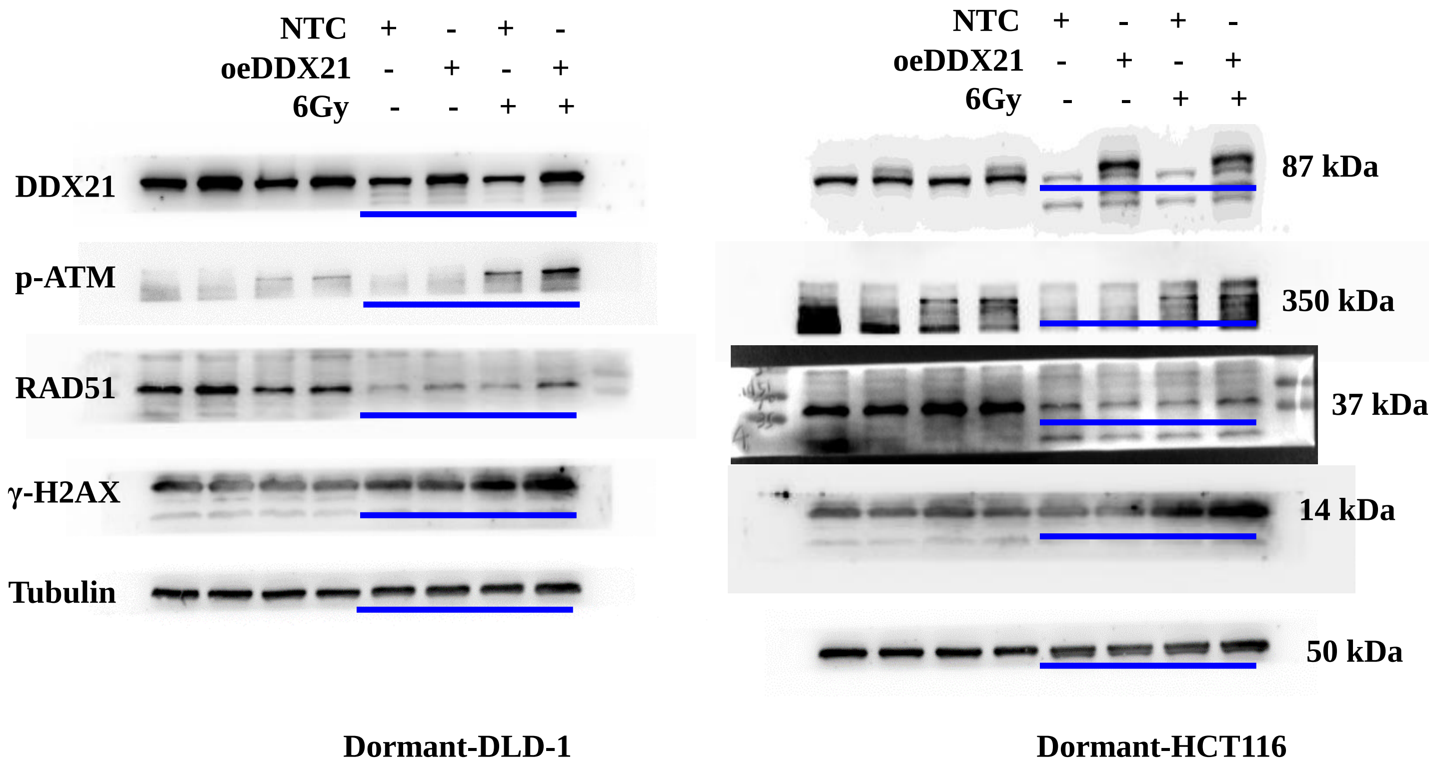

Fig. 7K

Western blots in the article

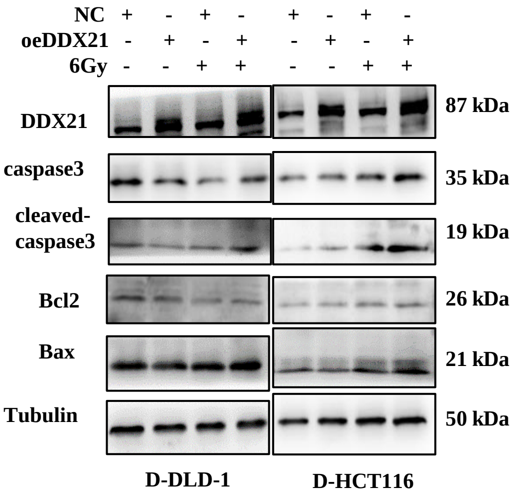

Original uncropped western

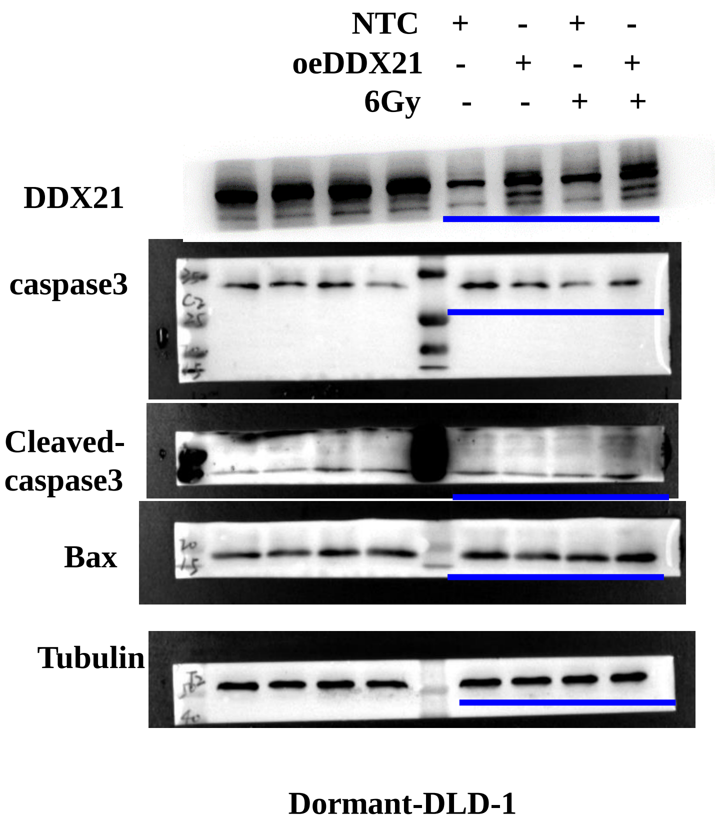

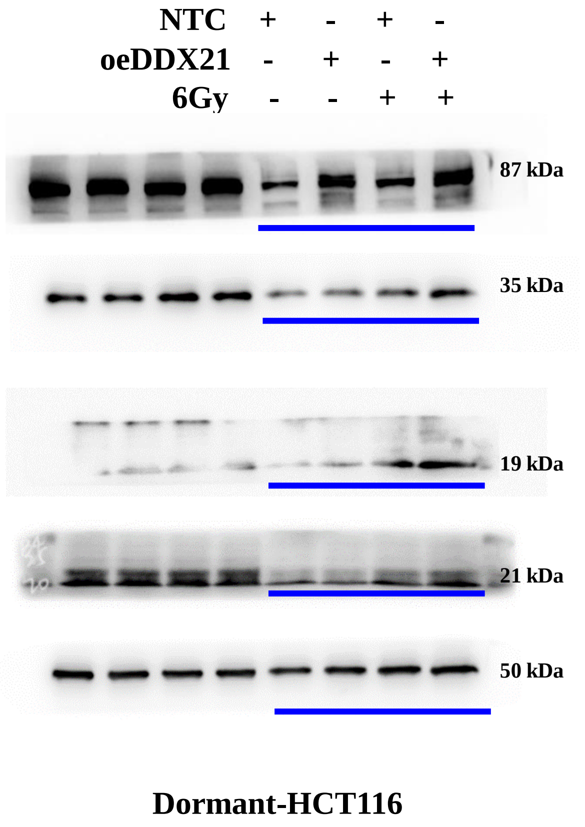

Fig. S3A

Western blots in the article

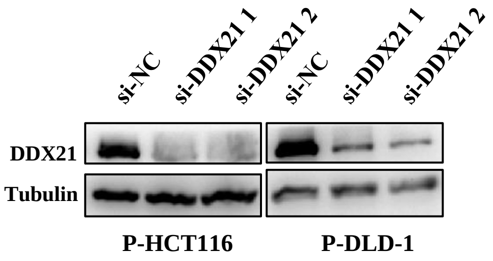

Original uncropped western

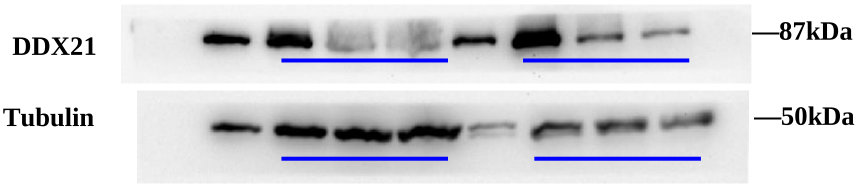

Fig. S3C

Western blots in the article

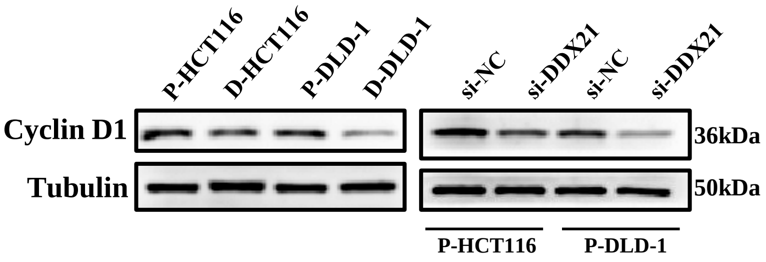

Original uncropped western

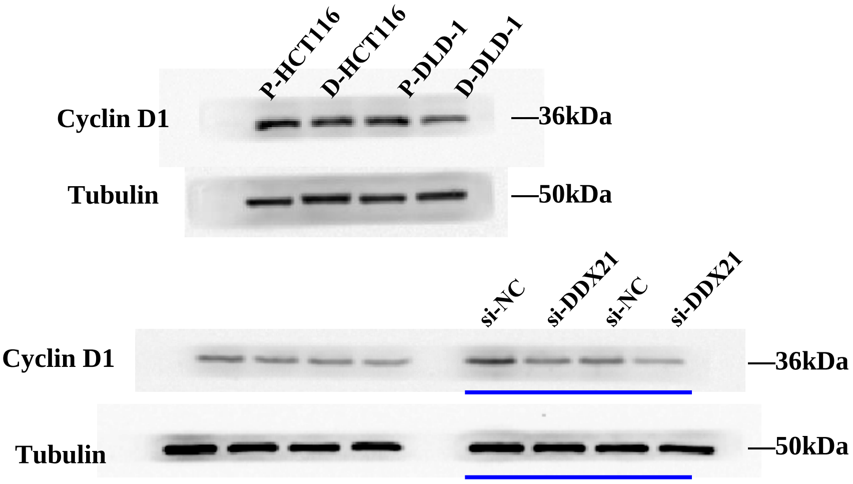

Fig. S3G

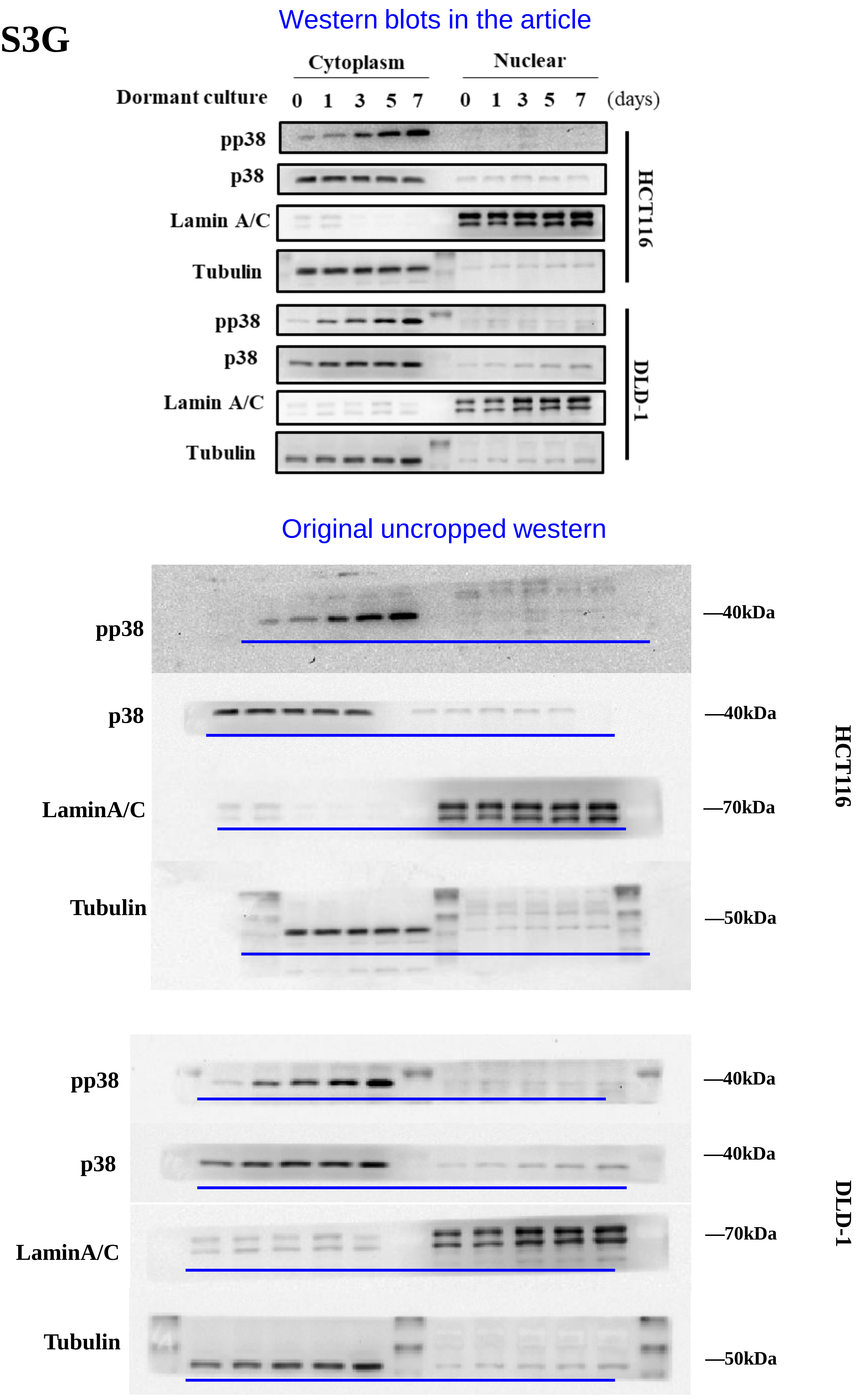

Western blots in the article
Cytoplasm
Nuclear
Dormant culture
0 1 3 5 7 0 1 3 5 7 (days)
pp38
p38
Lamin A/C
Tubulin
HCT116
pp38
p38
Lamin A/C
Tubulin
DLD-1
Original uncropped western
pp38
40kDa
p38
40kDa
LaminA/C
70kDa
Tubulin
50kDa
HCT116
pp38
40kDa
p38
40kDa
LaminA/C
70kDa
Tubulin
50kDa
DLD-1

Fig. S3H

Western blots in the article

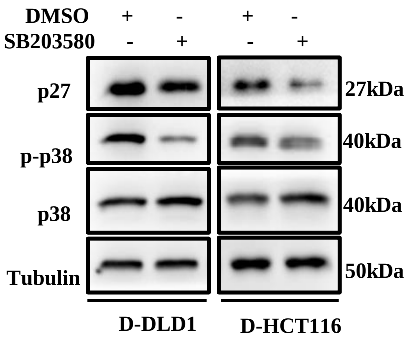

Original uncropped western

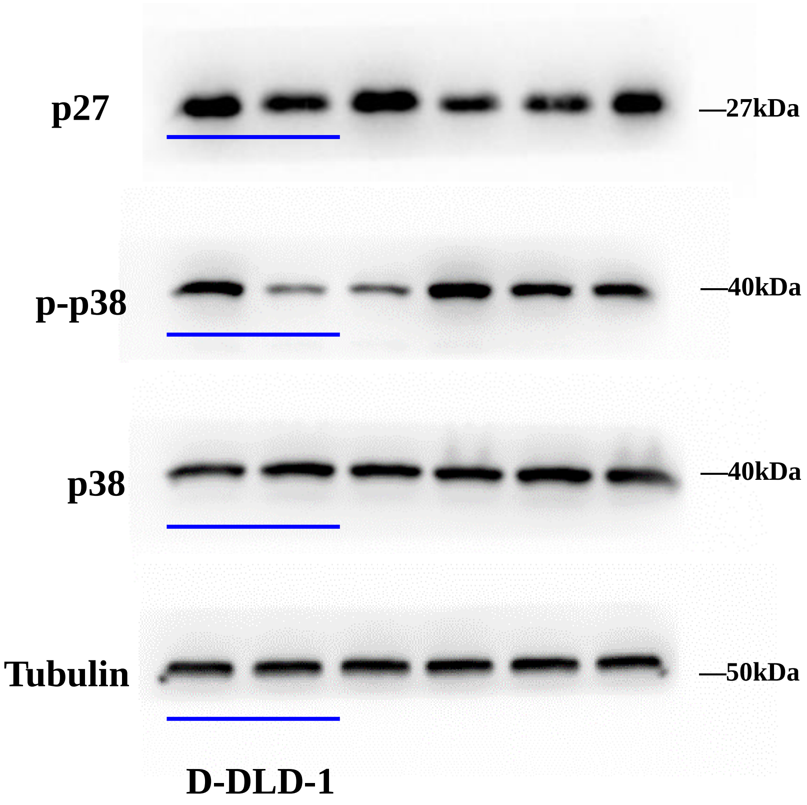

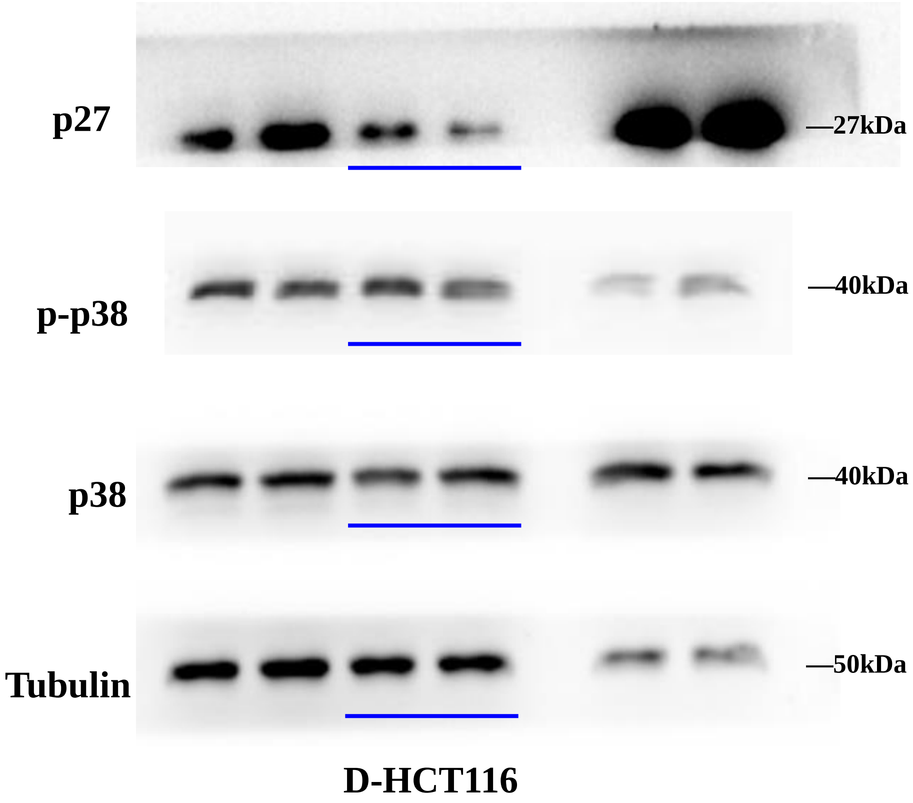

Fig. S4C

Original uncropped western

Western blots in the article

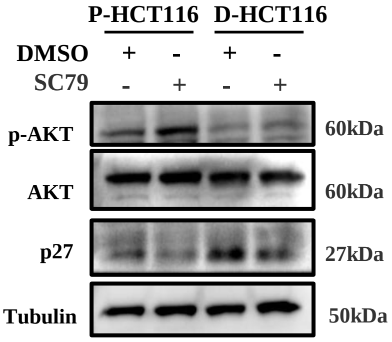

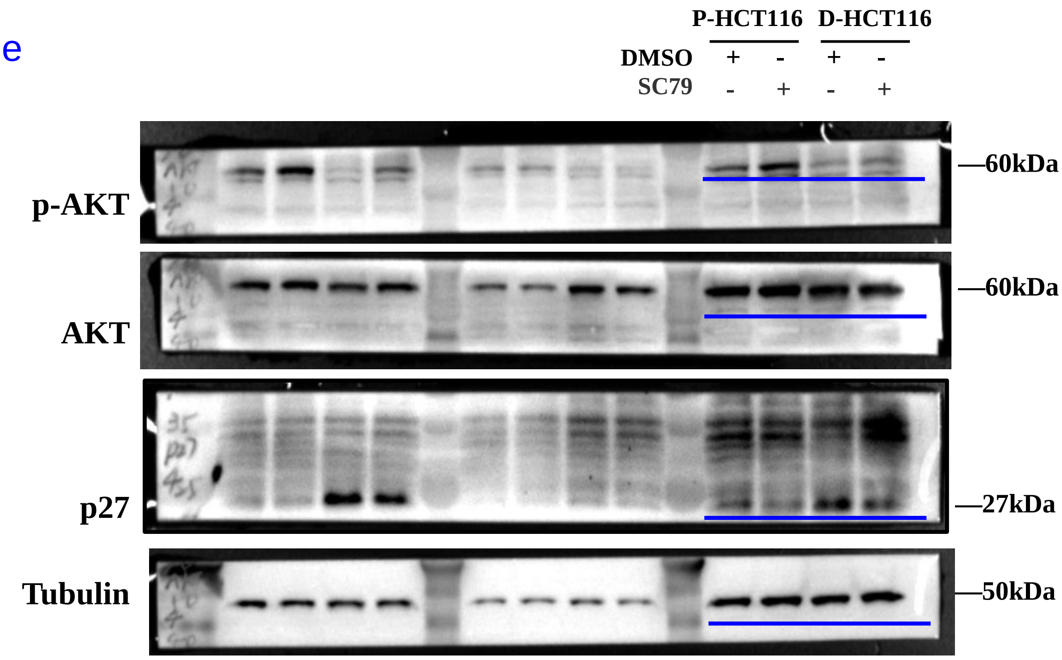

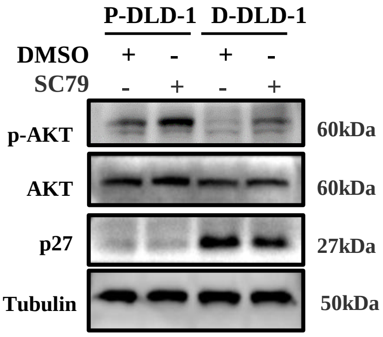

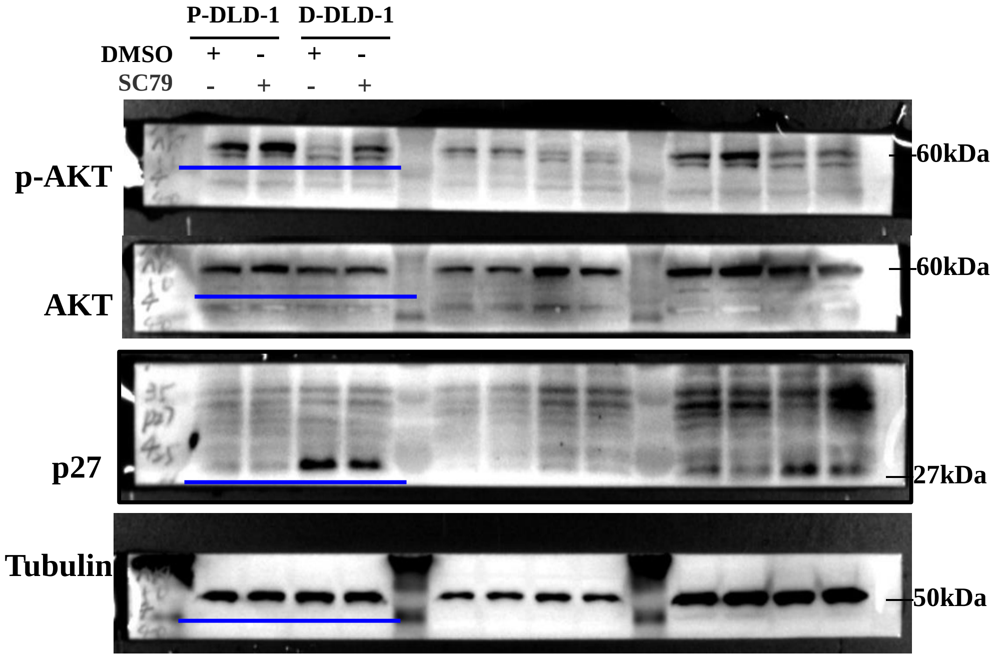

Fig. S4C

Western blots in the article

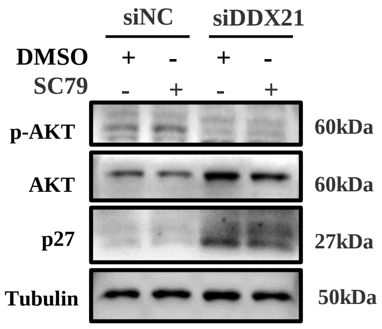

P-HCT116

Original uncropped western

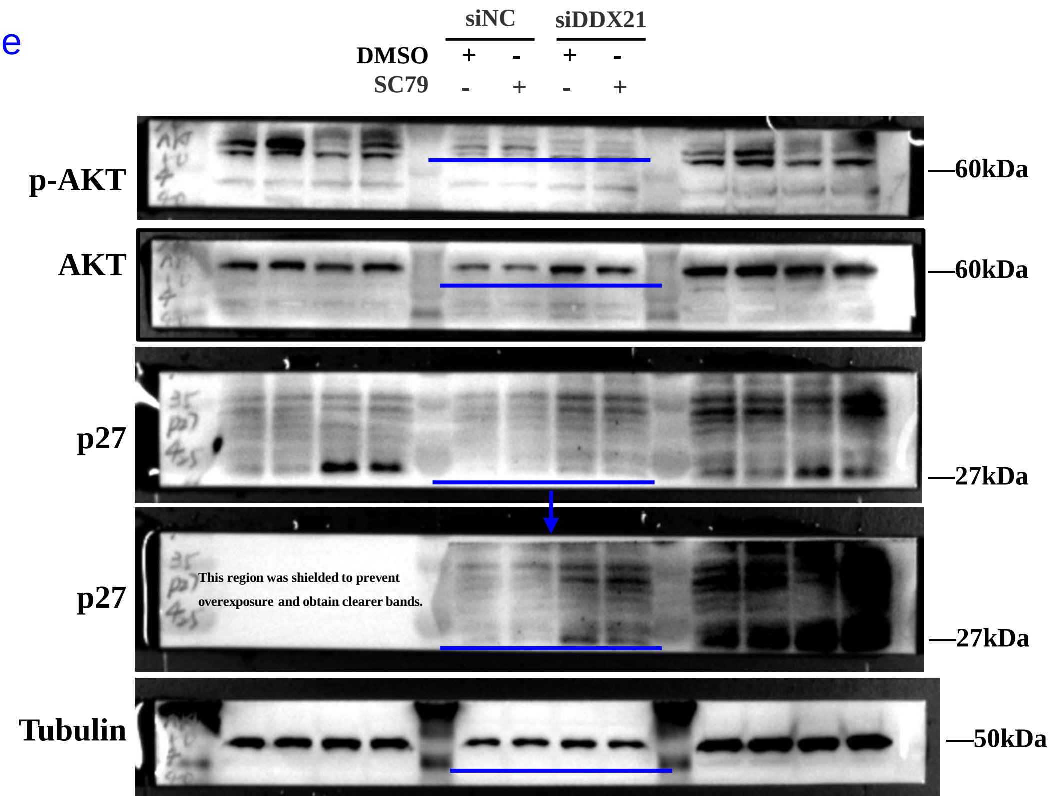

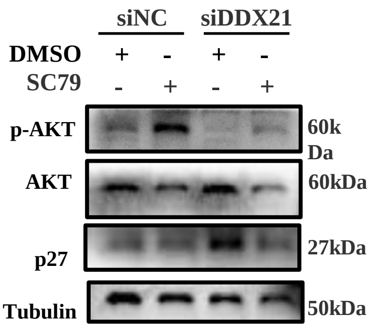

P-DLD-1

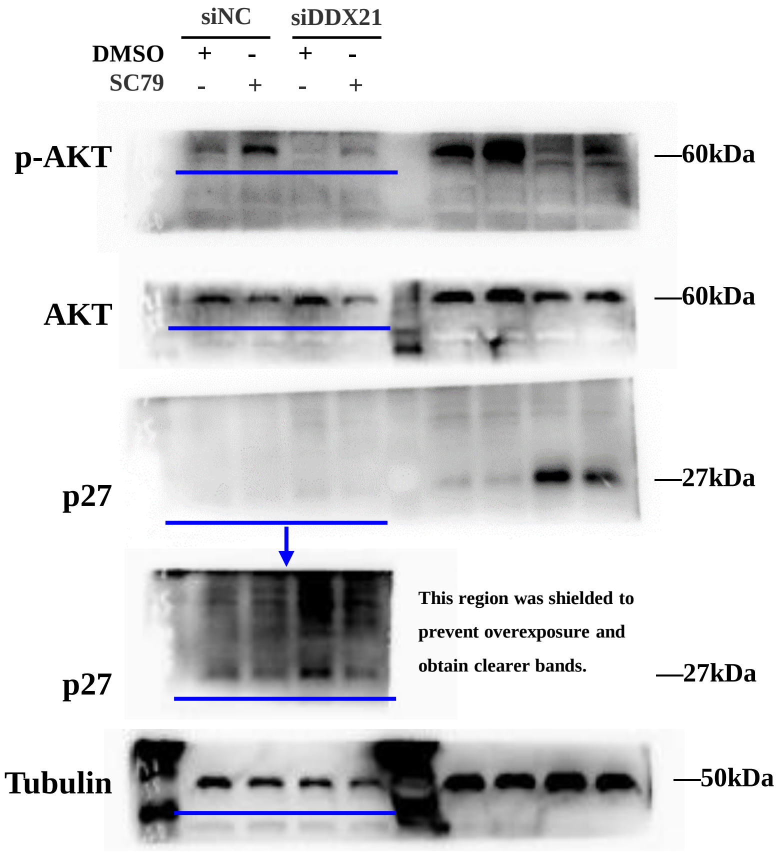

Fig. S4D

Original uncropped western

Western blots in the article

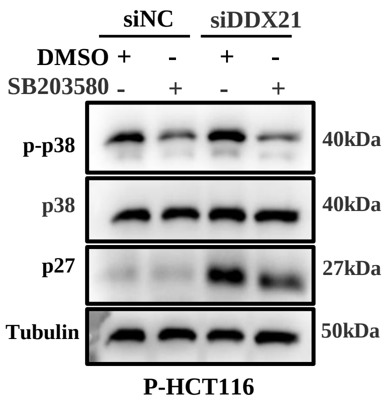

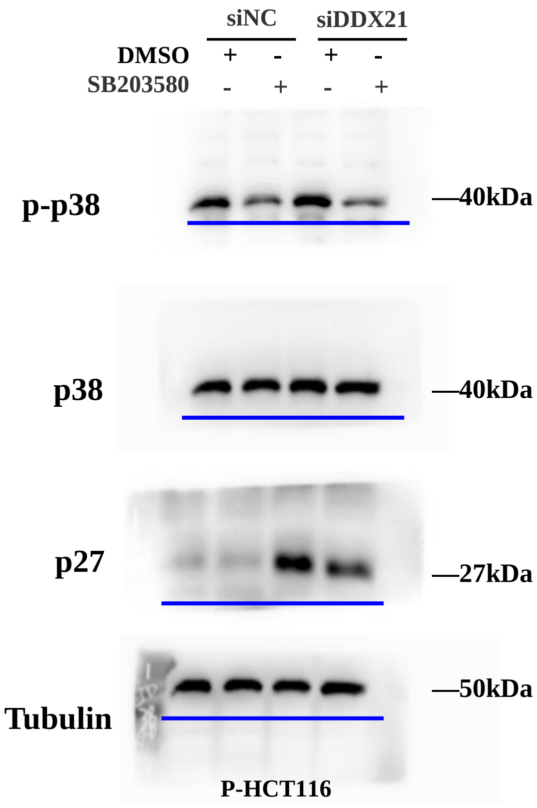

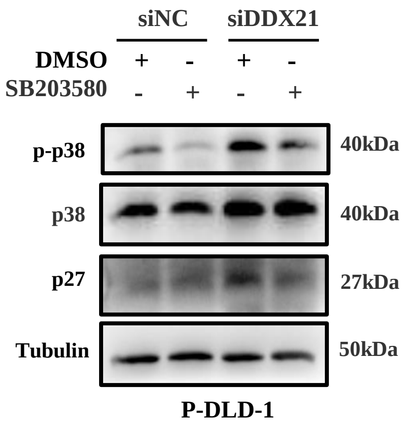

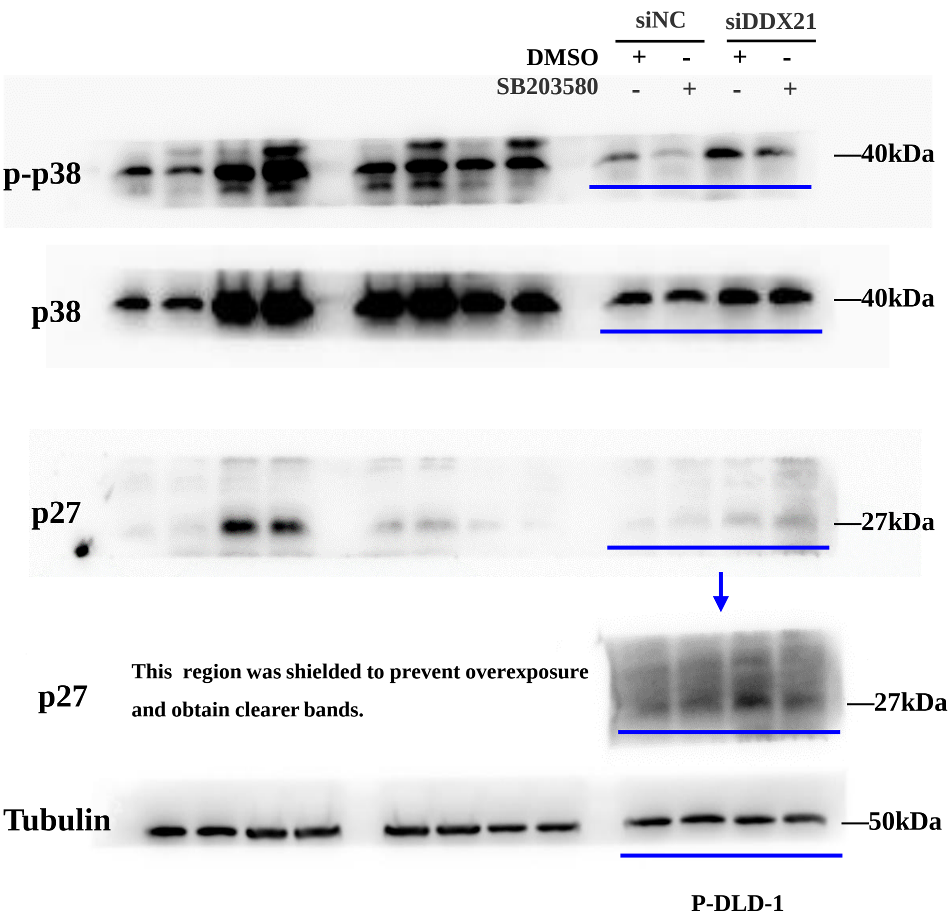

Fig. S4E

Original uncropped western

Western blots in the article

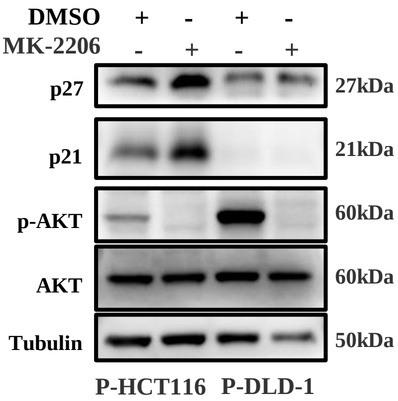

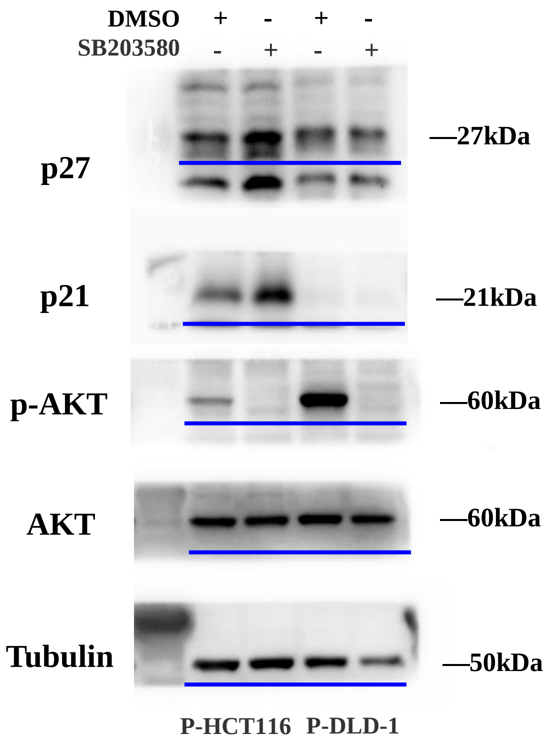

Fig. S4F

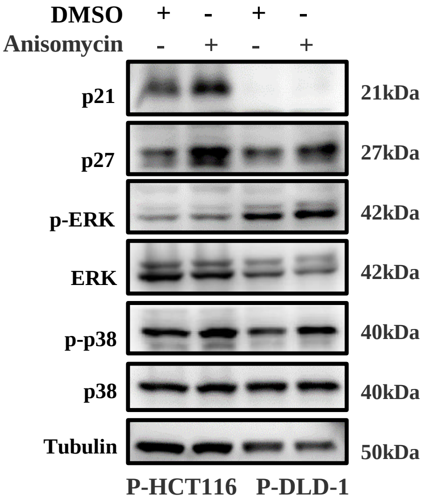

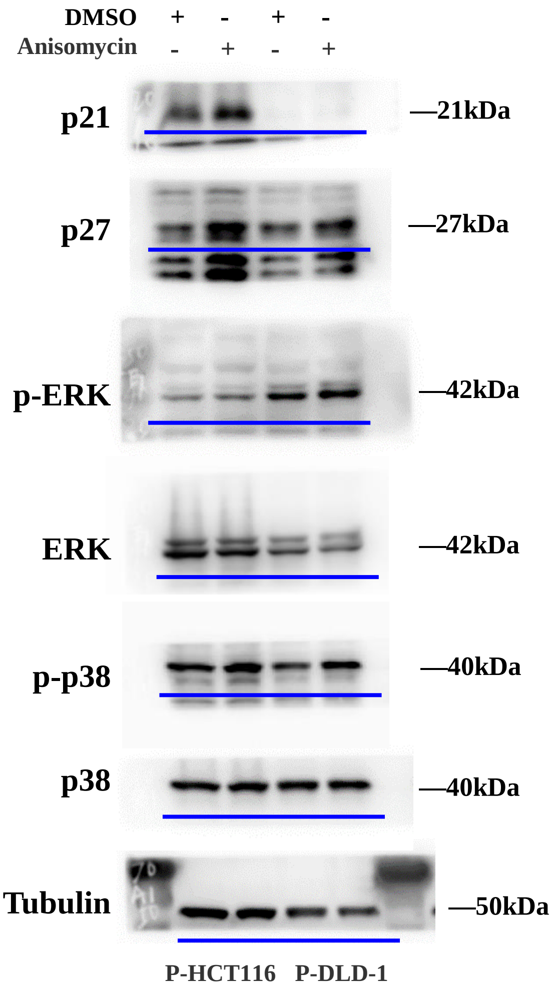

Fig. S6D

Western blots in the article

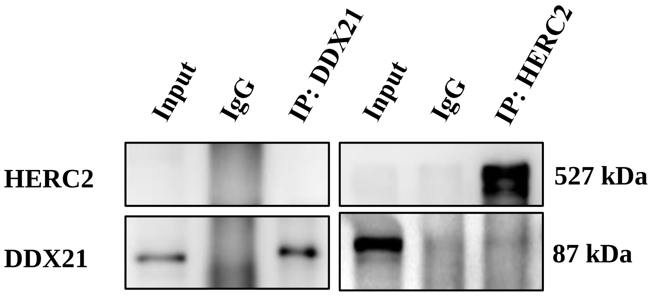

Proliferative-DLD-1

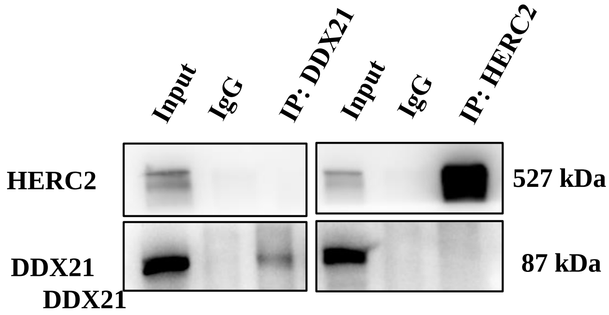

Proliferative-HCT116

Original uncropped western

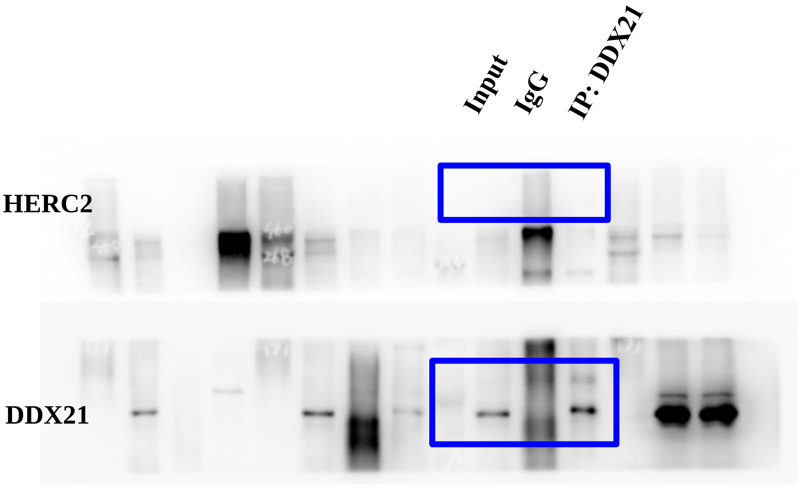

Proliferative-DLD-1

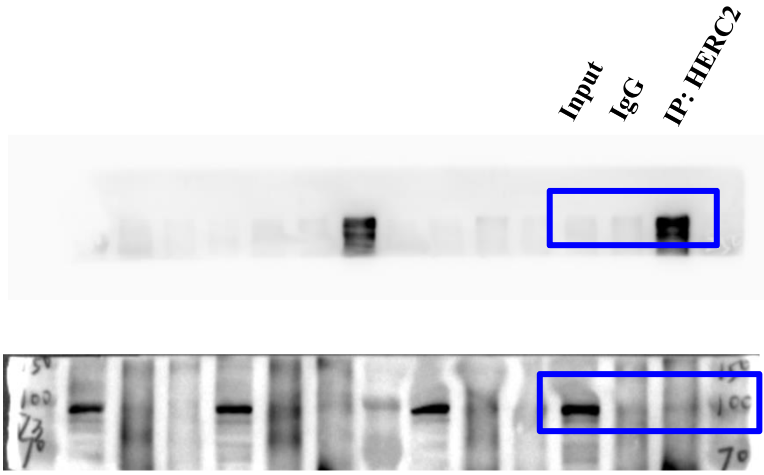

Proliferative-DLD-1

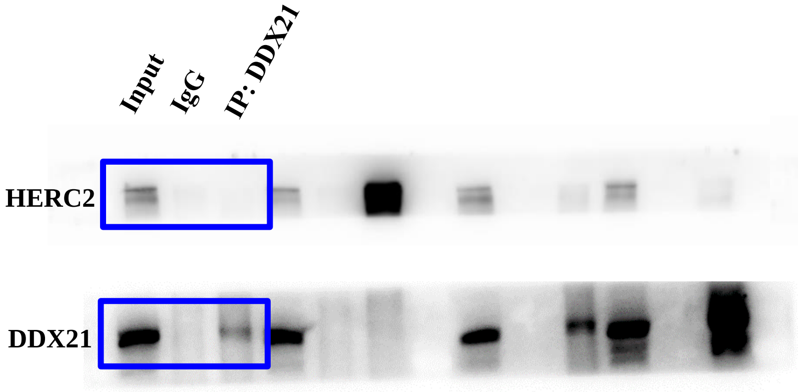

Proliferative-HCT116

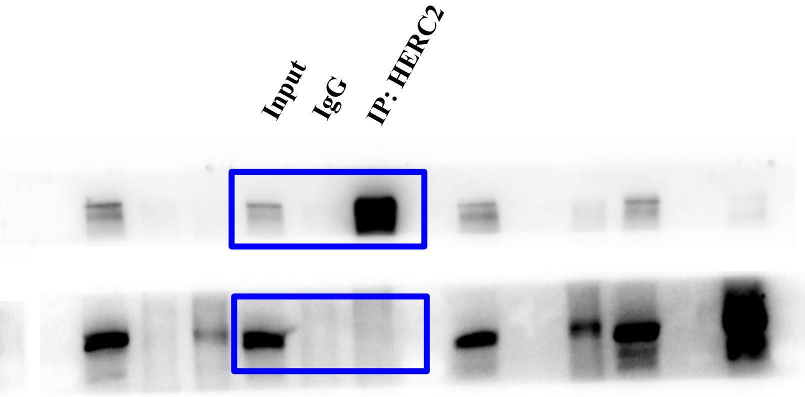

Proliferative-HCT116

Fig. S6E

Western blots in the article

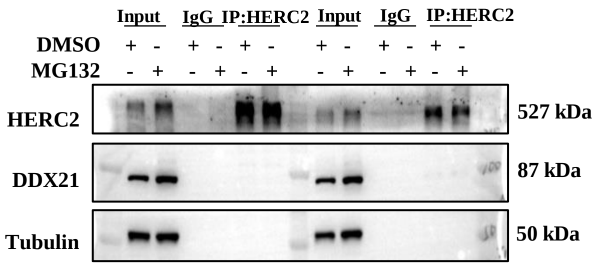

Original uncropped western

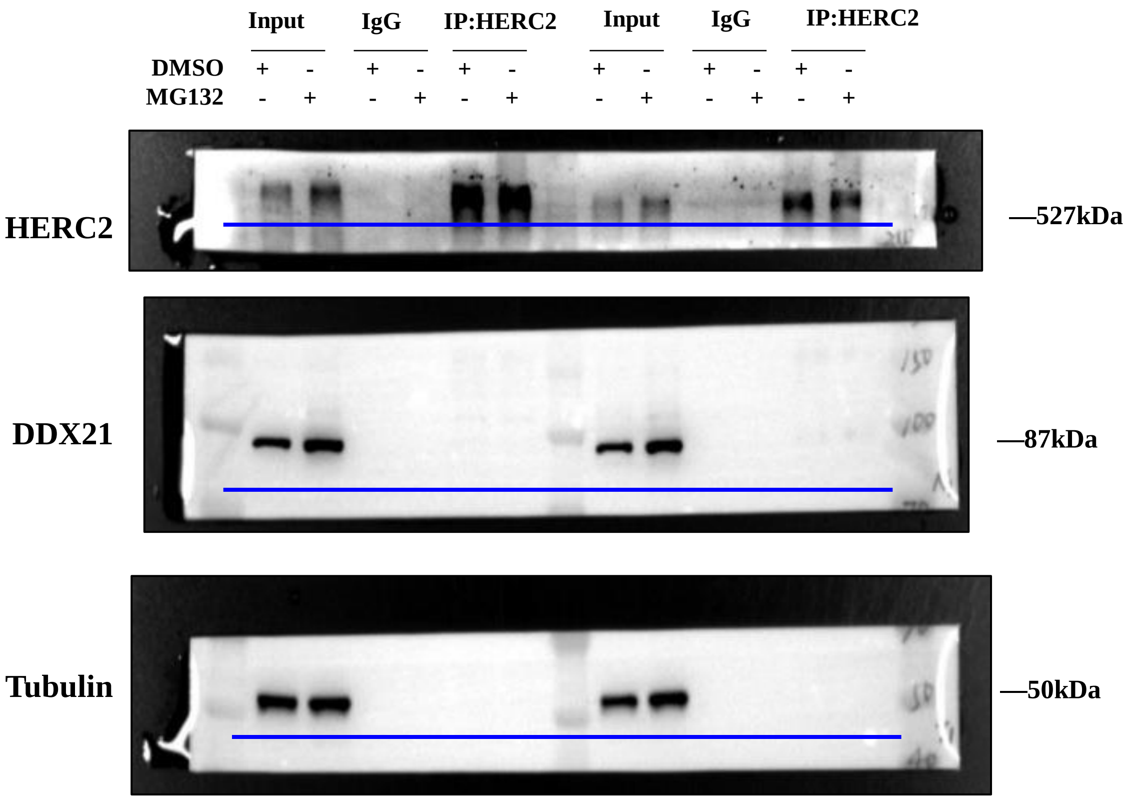

Fig. S6I

Western blots in the article

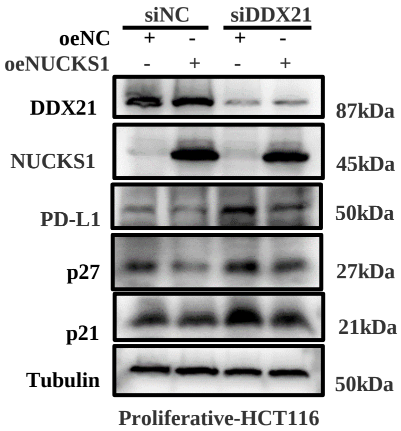

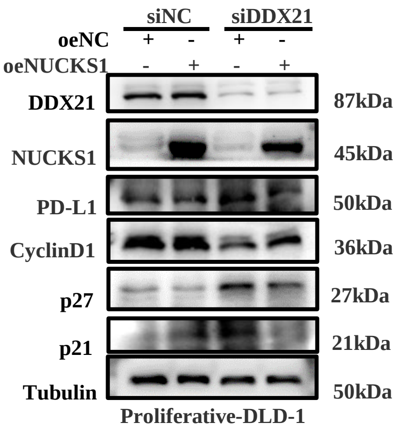

Original uncropped western

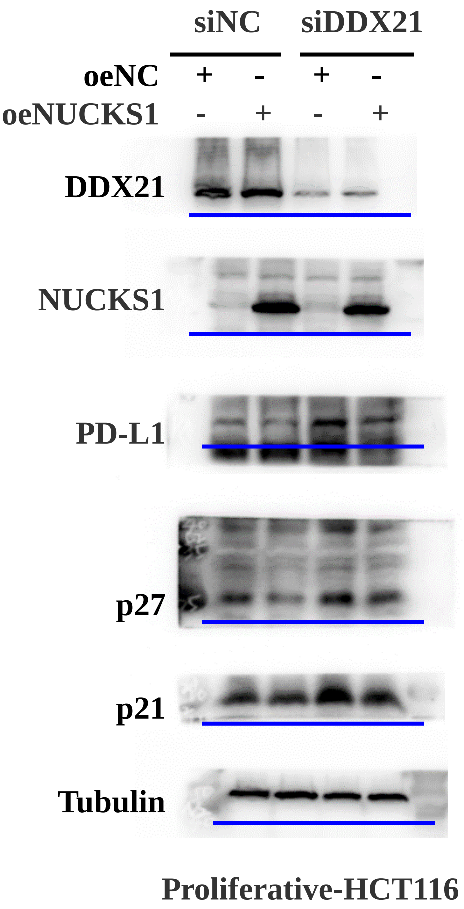

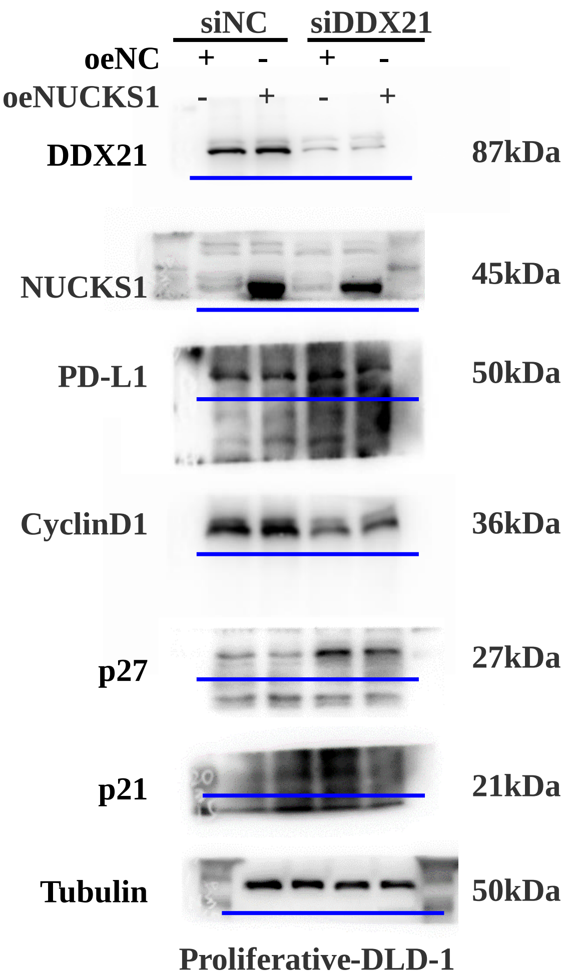

Fig. S6J

Western blots in the article

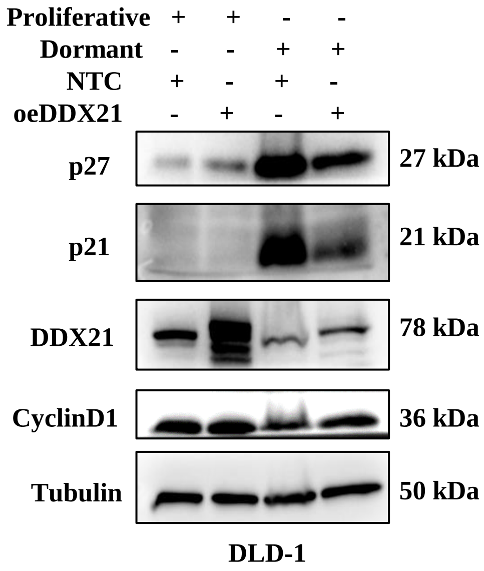

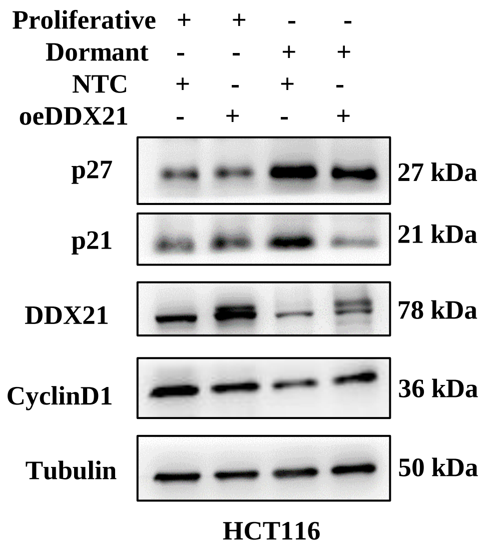

Original uncropped western

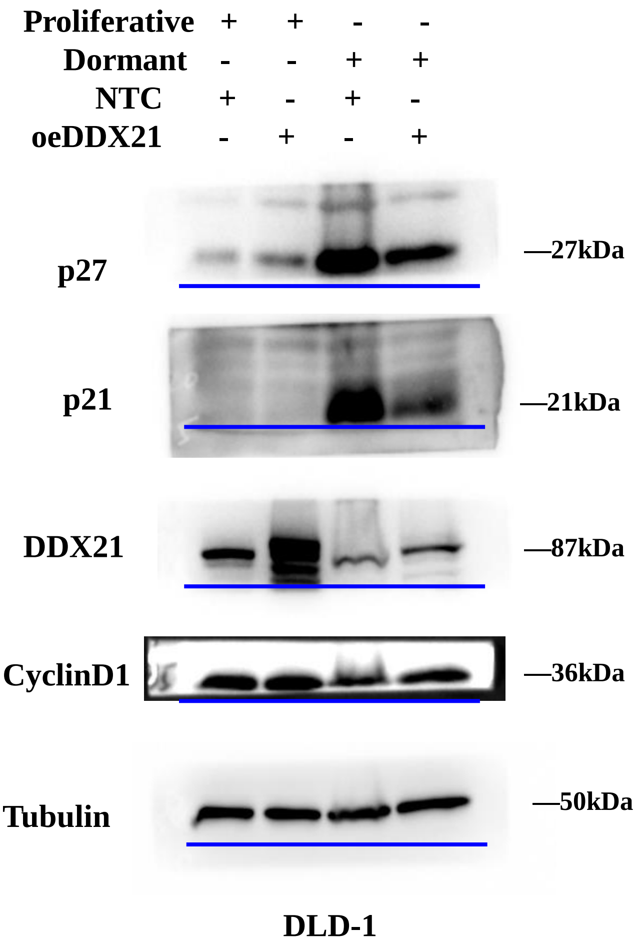

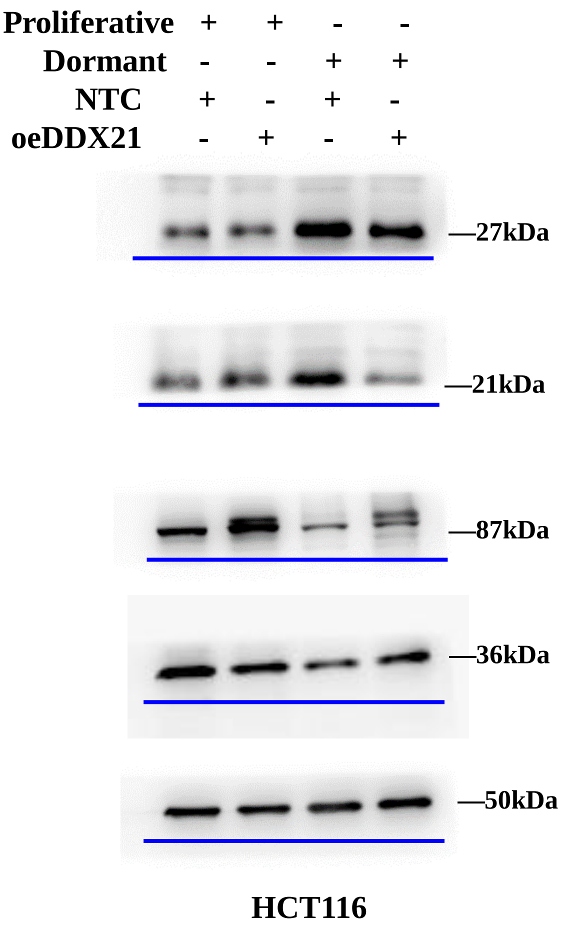

Supplement: Supplementary file 2 — Uncropped Western blots [file 41419_2026_8811_MOESM2_ESM.pdf]
